# Chloroplast Fibrillin‐Mediated α‐Tocopherol Biosynthesis Impaired by a Virus to Enhance Infection and to Improve Drought Tolerance

**DOI:** 10.1002/advs.202503696

**Published:** 2025-10-29

**Authors:** Sijia Liu, Xuedong Liu, Qin Yan, Xi Chen, Lianyi Zang, Jingang Hu, Xiaoping Zhu, Zaifeng Fan, Tao Zhou

**Affiliations:** ^1^ State Key Laboratory of Agricultural and Forestry Biosecurity Key Laboratory of Surveillance and Management for Plant Quarantine Pests Ministry of Agriculture and Rural Affairs, P. R. China Department of Plant Pathology China Agricultural University Beijing 100193 China; ^2^ College of Architectural Engineering Shenzhen Polytechnic University Shenzhen 518055 China; ^3^ Department of Plant Pathology College of Plant Protection Shandong Agricultural University Taian 271018 China; ^4^ Department of Tomato Breeding Zhengzhou Vegetable Research Institute Zhengzhou 450015 China; ^5^ National Citrus Engineering Research Center Citrus Research Institute Southwest University Chongqing 400712 China

**Keywords:** antioxidants, drought stress, fibrillin (FBN), retrograde signal, tomato chlorosis virus

## Abstract

Plastoglobules (PGs), the lipid droplets mainly within chloroplasts, are crucial for plant redox homeostasis and environmental adaptation. However, the regulation of PG under stress remains elusive. This study uncovers that PGs are targeted by the p22 protein encoded by an emerging virus‐tomato chlorosis virus (ToCV), leading to impaired α‐tocopherol biosynthesis, thereby facilitating viral infection and improving drought resistance. Specially, ToCV‐encoded p22 protein co‐opts the PG structural protein fibrillin (FBN) to access PG, where it impairs α‐tocopherol biosynthesis via disturbing the interaction between FBN1.1 and tocopherol cyclase (VTE1). Alpha‐tocopherol is required for both preventing lipid peroxidation in the thylakoid membrane and suppressing ToCV infection. Infection of ToCV or transgenic expression of p22 protein inhibits α‐tocopherol biosynthesis, resulting in chloroplast oxidative stress, which may contribute to the accumulation of 3′‐phosphoadenosine 5′‐phosphate (PAP), a retrograde signal from chloroplasts to the nucleus that triggers drought tolerance. Taken together, FBN1.1 modulates plant‐virus‐drought interaction via regulating the function of PG.

## Graphical abstract

ToCV‐encoded p22 targets chloroplast plastoglobules (PGs) via directly binding PG structural protein FBN1.1, reducing the size of PG and inhibiting α‐tocopherol biosynthesis via competing with tocopherol cyclase (VTE1). Consequently, the elevated chloroplast ROS not only creates a suitable cellular environment for efficient ToCV infection but also stimulates PAP accumulation, a retrograde signal from chloroplasts to the nucleus that triggers drought tolerance.

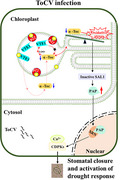

## 1 Introduction

Rapidly evolving climate conditions pose a serious threat to plant growth and crop yields, with rising atmospheric Carbon dioxide (CO_2_) levels and temperatures being the primary culprits, while water availability is concurrently diminishing. Drought has emerged as one of the most significant constraints to crop production globally.^[^ ^1^, ^2^, ^3^ ^]^ It is noteworthy that certain viral infections can confer drought tolerance to plants.^[^ ^2^, ^4^, ^5^, ^6^, ^7^ ^]^ The complex interplay between virus infections and drought stress signals in shaping plant fitness remains a central question of interest. Abscisic acid (ABA) is a key hormone that regulates water balance and stomatal function, thereby enhancing drought tolerance in plants.^[^ ^8^ ^]^ Infections of cucumber mosaic virus (CMV) and tomato yellow leaf curl virus (TYLCV) could induce drought tolerance through an ABA‐dependent or ABA‐independent manner.^[^ ^5^, ^6^ ^]^ Turnip mosaic virus confers drought tolerance associated with salicylic acid (SA).^[^ ^9^ ^]^ Additionally, viral small interfering RNA implicates in enhancing drought tolerance by boosting autophagy.^[^ ^7^ ^]^ A deeper understanding of the mechanisms underlying virus‐induced amelioration of drought stress could markedly contribute to improving plant adaptability to the challenges posed by climate change.

Reactive oxygen species (ROS) serve as crucial signaling molecules, enabling cells to rapidly respond to various stimuli.^[^ ^10^ ^]^ The chloroplast is a primary organelle for the production of ROS. Excessive ROS accumulation in the chloroplast is potentially damaging, whereas moderate levels can function as signaling molecules, triggering retrograde signaling that subsequently modulates plant stress responses by regulating nuclear gene expression.^[^ ^11^, ^12^ ^]^ It has been well characterized that 3′‐phosphoadenosine 5′‐phosphate (PAP) functions as a retrograde signal from chloroplasts to the nucleus for responses to drought and high light stresses.^[^ ^13^ ^]^ Under drought conditions, chloroplast oxidative stress inhibits the phosphatase activity of inositol polyphosphate1‐phosphatase SAL1, which acts a general sensor of ROS, leading to the subsequent accumulation of PAP.^[^ ^14^ ^]^ PAP inhibits 5′ to 3′ exoribonucleases, contributing to enhanced expression of drought‐responsive genes, particularly those encoding calcium‐dependent protein kinases (CDPKs), ultimately leading to improved plant drought tolerance.^[^ ^13^, ^15^ ^]^

To prevent oxidative damage, the chloroplast has evolved a sophisticated antioxidant system to balance ROS production and scavenging, with α‐tocopherol playing a crucial role in maintaining chloroplast redox homeostasis.^[^ ^16^ ^]^ As a lipophilic antioxidant compound in the vitamin E group, α‐tocopherol accounts for more than 90% of the foliar tocopherols in plants and is unique among the chloroplast network of antioxidants in that it can inhibit the propagation of lipid peroxidation.^[^ ^17^ ^]^ In chloroplasts, α‐tocopherol is mainly stored in plastoglobules (PGs) and can diffuse between PG and thylakoid via a continuous pathway formed between these two compartments.^[^ ^18^ ^]^ The level of α‐tocopherol is primarily regulated by tocopherol cyclase enzyme VTE1, which plays a pivotal role in both biosynthesis and recycling of α‐tocopherol by being localized in PG.^[^ ^18^, ^19^ ^]^

PGs are globular lipid droplets located in the curved regions of thylakoid membrane within chloroplasts, performing crucial roles in plastid metabolism, redox balance, photosynthetic regulation, and environmental adaptation of plants.^[^ ^20^ ^]^ The abundance and size of PG increase in conjunction with thylakoid disintegration, nitrogen deficiency, high‐light stress, and leaf senescence.^[^ ^21^, ^22^ ^]^ Structurally, PGs are enveloped by a layer of proteins comprising fibrillin (FBN), which form a protective coating around the microcompartments. Previous studies have established that FBNs exhibit responsiveness to pathogens and drought.^[^ ^23^, ^24^ ^]^ Therefore, it is worthwhile to delve into the interplay between FBN, drought, and viral infections.

It has been reported that viral infections actively disrupt the delicate balance of redox homeostasis within chloroplasts.^[^ ^25^ ^]^ During infection, viral proteins may enter into chloroplasts via diverse ways, where these proteins impair photosynthesis, interfere with the biosynthesis of disease resistance‐related hormones, and upset the redox balance. Consequently, the chloroplast‐mediated capacity to mount direct or indirect defense against diseases is compromised.^[^ ^26^ ^]^ Most recently, we found that the p22 protein encoded by tomato chlorosis virus (ToCV) might interact with several chloroplast proteins of tomato (*Solanum lycopersicum*).^[^ ^27^ ^]^ Moreover, infection of ToCV significantly downregulates photosynthesis‐related genes.^[^ ^28^ ^]^ Considering the crucial roles of p22 for the pathogenicity of ToCV,^[^ ^29^, ^30^ ^]^ we formulated a hypothesis that p22 might modulate chloroplast function in a way to facilitate the establishment of robust infections.

ToCV (genus *Crinivirus*, family *Closteroviridae*) is a globally pervasive pathogen capable of infecting more than 80 plant species across 25 distinct families.^[^ ^31^, ^32^ ^]^ On most host plants, it manifests as mild interveinal chlorosis or yellowing of older leaves, ultimately decreasing fruit production and quality.^[^ ^31^, ^32^ ^]^ First identified in Florida (USA), ToCV has subsequently spread predominantly to temperate and tropical regions, with a notable concentration in greenhouse and field environments where seasonal drought conditions or inadequate water management practices exacerbate its incidence.^[^ ^32^ ^]^ In this study, we uncovered that infection of ToCV enhances drought tolerance by activating the SAL1‐PAP retrograde signaling pathway. Specially, the ToCV‐encoded p22 protein co‐opts FBN to access PG, where it competes with VTE1 to disrupt the biosynthesis of α‐tocopherol. This impairment was associated with elevated chloroplast oxidative stress and subsequent accumulation of PAP, which might contribute to drought stress adaptation mechanisms. Furthermore, our findings indicate that α‐tocopherol exhibits antiviral activity. Collectively, these findings highlight chloroplast PG as an innovative viral target, with roles in manipulating chloroplast oxidative homeostasis. Notably, PG functions as a convergence point for both biotic‐ and abiotic‐stress signals, participating in regulating antiviral and drought resistance signaling pathways.

## 2 Results

### 2.1 The p22 Protein is Responsible for ToCV‐Induced Drought Tolerance via Stimulating SAL1‐PAP Retrograde Signaling in Plants

**Figure 1:**
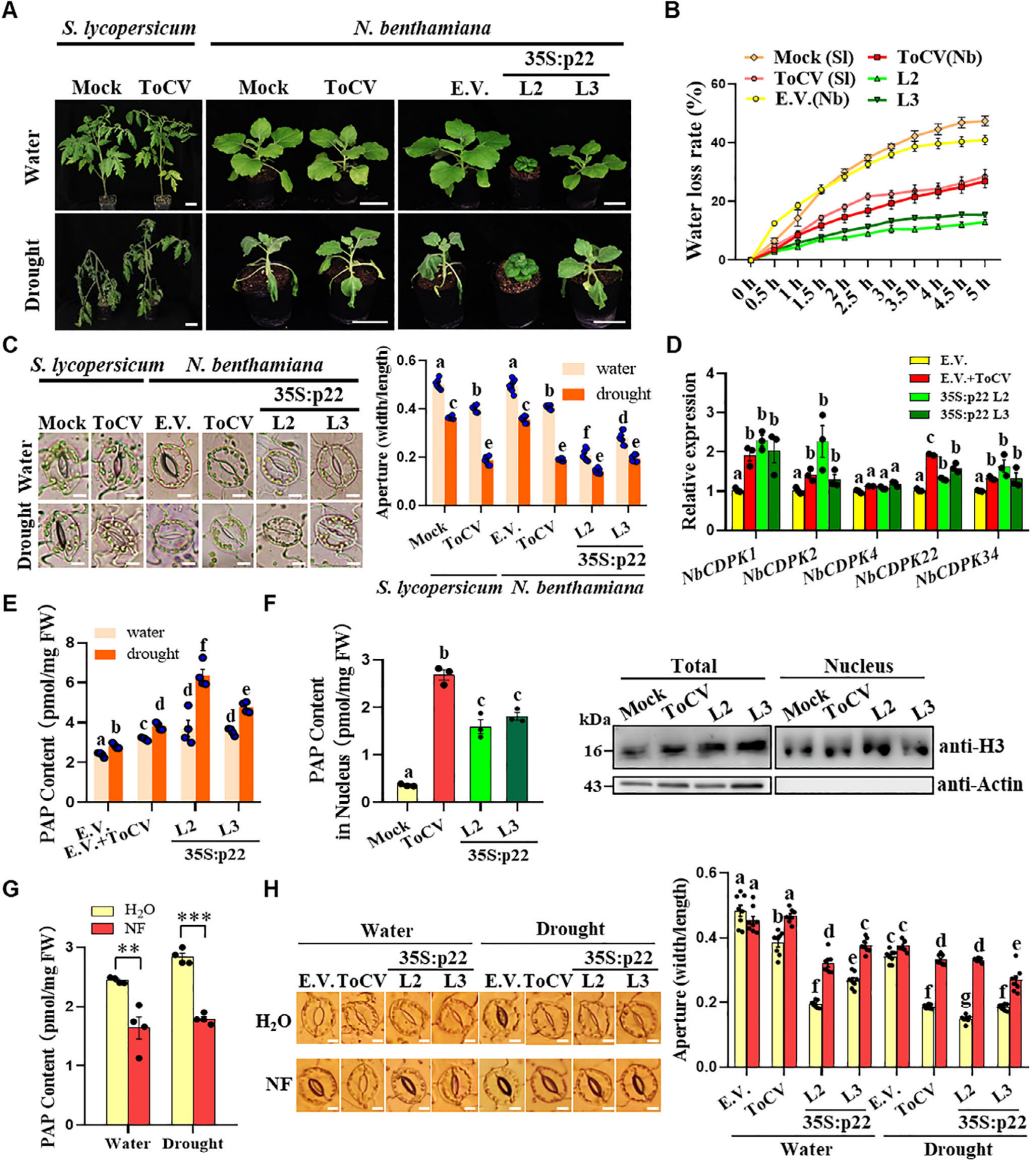
Infection of tomato chlorosis virus (ToCV) or transgenic expression of p22 protein increases drought tolerance through stimulating SAL1‐PAP retrograde signaling. A) Infection of ToCV or transgenic expression of p22 (35S:p22) increased drought tolerance of tomato (*Solanum lycopersicum*) and *Nicotiana benthamiana* plants. Bars = 5 cm. The plants were photographed at 10 days post‐drought treatment. B) Water loss rates were reduced in ToCV‐infected tomato and *N. benthamiana* plants or p22‐transgenic plants. C) Representative stomatal images and stomatal apertures of mock and ToCV‐inoculated *S. lycopersicum*, E.V. (transgenic empty vector control), ToCV‐infected E.V., and p22‐transgenic *N. benthamiana* plants under normal and drought conditions. Bars = 10 µm. The stomatal apertures were indicated by the ratio of width to length. The stomal images were photographed at 5 days post drought treatment. D) Expression levels of Ca^2+^ signaling‐related *CDPK* genes in E.V., ToCV‐infected E.V., and p22‐transgenic plants. The expression level of the *NbActin* gene was used as an internal control. E) PAP contents in E.V., ToCV‐infected E.V., and p22‐transgenic plants under normal and drought conditions. F) Measurement of nuclear PAP contents. Nucleus was isolated and confirmed by the extraction of nuclear protein and immunoblotting using anti‐H3 (nuclear marker) and anti‐Actin (cytoplasmic marker) antibodies. G) The PAP accumulation levels were significantly down‐regulated in NF‐sprayed plants under both normal water and drought conditions. H) Representative stomatal images and stomatal apertures of H_2_O‐ or NF‐sprayed E.V., ToCV‐infected E.V., and p22‐transgenic expression plants under normal water and drought conditions. Bars = 10 µm. The different letters above the bars indicate the statistically significant differences between the treatments, determined by a one‐way ANOVA test followed by Tukey's multiple test (*p* < 0.05). **, *p *< 0.01, ***, *p *< 0.001; determined using the two‐tailed Student's *t*‐test. Error bars were SEM. These experiments were performed two times, and six biological replicates were used per treatment.

During the course of plant cultivation, we observed that ToCV‐infected tomato seedlings exhibited a notably higher drought tolerance (**Figure** **1**A). Subsequently, we confirmed that *Nicotiana benthamiana* plants also displayed enhanced drought tolerance either when infected with ToCV or transgenic expression of p22 (Figure 1A). Consistently, the water loss rates were significantly reduced in ToCV‐infected *S. lycopersicum* and *N. benthamiana*, as well as in p22‐transgenic plants (Figure 1B). Moreover, the stomatal apertures decreased significantly following ToCV infection or with p22‐transgenic expression (Figure 1C).

ABA is the primary hormone responsible for regulating drought responses.^[^ ^33^ ^]^ However, ABA‐responsive genes were not substantially induced by ToCV infection or p22‐transgenic expression, either under normal water or drought conditions (Figure S1, Supporting Information). Considering that the up‐regulated expression of *CDPKs* also leads to stomatal closure and enhanced drought tolerance,^[^ ^13^ ^]^ we analyzed the expression levels of *NbCDPKs*. Notably, *NbCDPK1*, *NbCDPK2*, *NbCDPK22*, and *NbCDPK34*, were significant upregulated following ToCV infection or p22‐transgenic expression (Figure 1D). Furthermore, the accumulation levels of PAP, a marker for the activity of SAL1‐PAP retrograde signaling^[^ ^14^ ^]^ were markedly elevated in both total tissues and nuclear fractions in ToCV‐infected or p22‐transgenic plants than their controls (Figure 1E,F).

To further verify the activation of SAL1‐PAP retrograde signaling following ToCV infection or p22‐transgenic expression, we employed norflurazon (NF) treatment, which is known to suppress the expression of photosynthesis‐related nuclear genes via disrupting chloroplast retrograde signaling.^[^ ^34^ ^]^ By applying NF, we successfully inhibited the retrograde signaling, as evidenced by the significantly reduced expression levels of *NbLHCB2.1* and *NbCA1* (Figure S2, Supporting Information). Additionally, the PAP content was decreased in NF‐sprayed plants (+NF) compared with the control (+H_2_O) under both normal water and drought conditions (Figure 1G). Furthermore, the expression levels of *NbCDPK2*, *NbCDPK4*, and *NbCDPK32* decreased in ToCV‐infected or p22‐transgenic plants following NF treatment compared with control treatment (Figure S2, Supporting Information). Notably, the stomatal apertures were increased in NF‐treated plants compared with the control under both normal water and drought conditions (Figure 1H). These results indicate that SAL1‐PAP retrograde signaling is indeed activated following ToCV infection or with p22‐transgenic expression.

### 2.2 P22 Induces an Elevation in Chloroplast Lipid Oxidation during ToCV Infection

**Figure 2:**
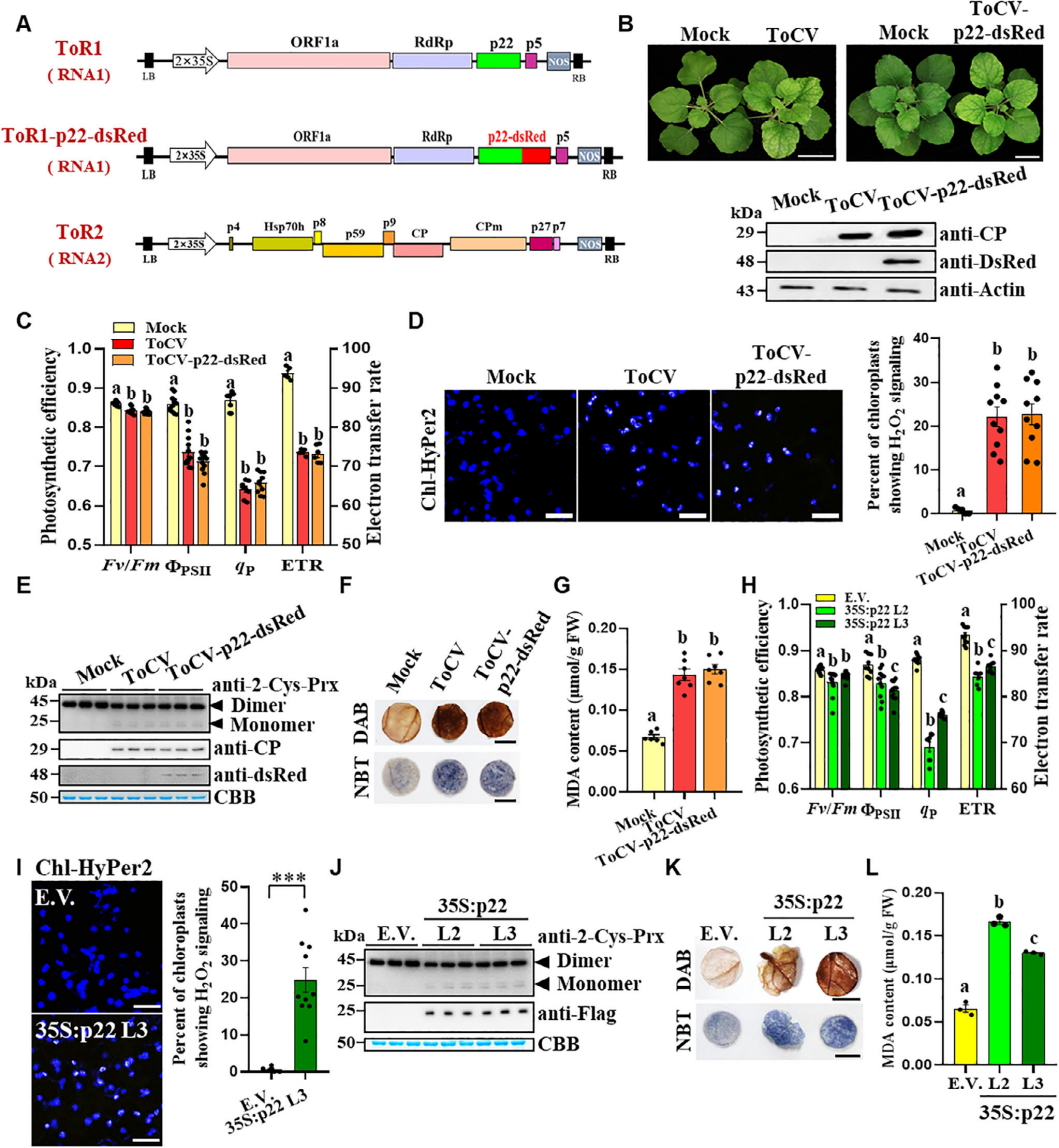
P22 of ToCV increases lipid oxidation of chloroplasts. A) Schematic illustrations of ToCV‐based infectious constructs. The ToR1 construct expresses a full‐length RNA1 of the ToCV genome. The ToR1‐p22‐dsRed construct carries a *dsRed* gene at the 3′ end of *p22*. The ToR2 construct expresses a full‐length RNA2 of the ToCV genome. B) ToCV and ToCV‐p22‐dsRed infection caused similar chlorosis symptoms. Plants were photographed at 21 dpi. Bars = 5 cm. Immunoblotting used anti‐CP and anti‐dsRed. C) *Fv*/*Fm*, Φ_PSII_, *q*_P_, and ETR in the upper leaves of the above plants at 21 dpi. D) Chl‐HyPer2 response to chloroplast H_2_O_2_ elicited by ToCV or ToCV‐p22‐dsRed infection. The blue color indicates the chloroplast. Bars = 25 µm. The right panel shows the quantification of ToCV‐induced changes in Chl‐HyPer2 fluorescence. The percentage of H_2_O_2_‐positive chloroplasts (white) among all chloroplasts (blue+white) per visual field is shown. E) The 2‐Cys Prx redox status in the above plants was determined using non‐reducing SDS‐PAGE. F) Histochemical staining of the above plant upper leaves using DAB for H_2_O_2_ production and NBT for O_2_^−^ production. Bars = 1 cm. G) MDA content in the above plants upper leaves. FW, fresh weight. H) *Fv*/*Fm*, Φ_PSII_, *q*_P_, and ETR in E.V. and p22‐transgenic plants. I) Chl‐HyPer2 response to chloroplast H_2_O_2_ in E.V. and p22‐transgenic plants. Bars = 25 µm. The right panel is the quantification of the p22‐dependent changes in Chl‐HyPer2 fluorescence. Asterisks indicate the statistically significant differences between the treatments (***, *p* < 0.001), determined using the two‐tailed Student's *t*‐test. J) 2‐Cys Prx redox status in E.V. and p22‐transgenic lines. K) Histochemical staining of E.V. and p22‐transgenic lines using DAB for H_2_O_2_ production and NBT for O_2_^−^ production. Bars = 1 cm. L) MDA content in E.V. and p22‐transgenic lines. Different letters above the bars in (C,D,G,H,L) indicate the statistically significant differences between treatments, determined using the one‐way ANOVA test followed by Tukey's multiple test (*p *< 0.05). Error bars were SEM. The Coomassie Brilliant Blue (CBB)‐stained rubisco large subunit gel in (E,J) was used to show sample loadings. These experiments were performed three times and had at least six biological replicates per treatment.

Given that SAL1 activity in plants is sensitive to the overall redox state of the chloroplast,^[^ ^14^ ^]^ we hypothesized that p22 might stimulate SAL1‐PAP‐mediated retrograde signaling by inducing chloroplast oxidative stress. In order to further explore the function of p22, we modified the genomic RNA1 of ToCV‐Beijing isolate (ToCV‐BJ)^[^ ^35^ ^]^ by fusing a discosoma red fluorescent protein (dsRed) to the C‐terminus of p22, resulting in ToR1‐p22‐dsRed (**Figure** **2**A). The resultant virus, designated as ToCV‐p22‐dsRed, containing ToR1‐p22‐dsRed and ToR2 (RNA2), was capable of successfully infecting *N. benthamiana*, causing chlorosis symptoms and expressing the coat protein (CP) that was comparable to that observed in wild‐type ToCV‐BJ infections (Figure 2B).

As expected, we observed that infection of wide‐type ToCV‐BJ or ToCV‐p22‐dsRed infection significantly impaired the efficiency of PSII (*Fv*/*Fm*), the quantum yield of PSII electron transport (Φ_PSII_), the photochemical quenching coefficient (*q*_P_), and the electron transfer rate (ETR) compared with mock‐inoculated plants (Figure 2C). To monitor the chloroplast redox states, we utilized a chloroplast fluorescent hydrogen peroxide (H_2_O_2_) sensor, that is, the Chl‐HyPer2 construct, as previously described.^[^ ^25^, ^36^ ^]^ Notably, ≈20% of chloroplasts exhibited detectable H_2_O_2_ signals in ToCV‐infected leaves, whereas no detectable H_2_O_2_ signals were observed in chloroplasts of mock‐, CMV‐, or tobacco rattle virus (TRV)‐infected plants (Figure 2D; Figure S3, Supporting Information). Consistently, analysis of the redox forms of the 2‐Cys Prx protein revealed an increase in 2‐Cys Prx monomers following ToCV infection, confirming the induction of chloroplast oxidative stress (Figure 2E). Moreover, the levels of total H_2_O_2_ and superoxide radicals (O_2_^−^) in ToCV‐infected leaves were significantly elevated compared with mock controls (Figure 2F). Additionally, ToCV infection significantly increased lipid oxidation, as evidenced by measurements of malondialdehyde (MDA), a marker of lipid oxidation (Figure 2G).

To further investigate the potential role of p22 in modulating the chloroplast redox state, we detected that transgenic expression of p22 (L2 and L3) resulted in a reduction of *Fv*/*Fm*, Φ_PSII_, *q_P_*, and ETR values (Figure 2H). This suppression was accompanied by increased chloroplast oxidative stress, as detected by both Chl‐HyPer2 imaging and 2‐Cys Prx monomers detection in L2 and L3 plants (Figure 2I,J). Furthermore, the levels of H_2_O_2_, O_2_^−^, and MDA were significantly elevated in L2 and L3 plants compared with the empty vector (E.V.) control plants (Figure 2K,L). Taken together, these results indicate that p22 plays a significant role in contributes to the chloroplast oxidative state during ToCV infection.

### 2.3 P22 Co‐Opts NbFBN1.1 to Access Chloroplast PG via Binding to the Mature Domain of NbFBN1.1

**Figure 3:**
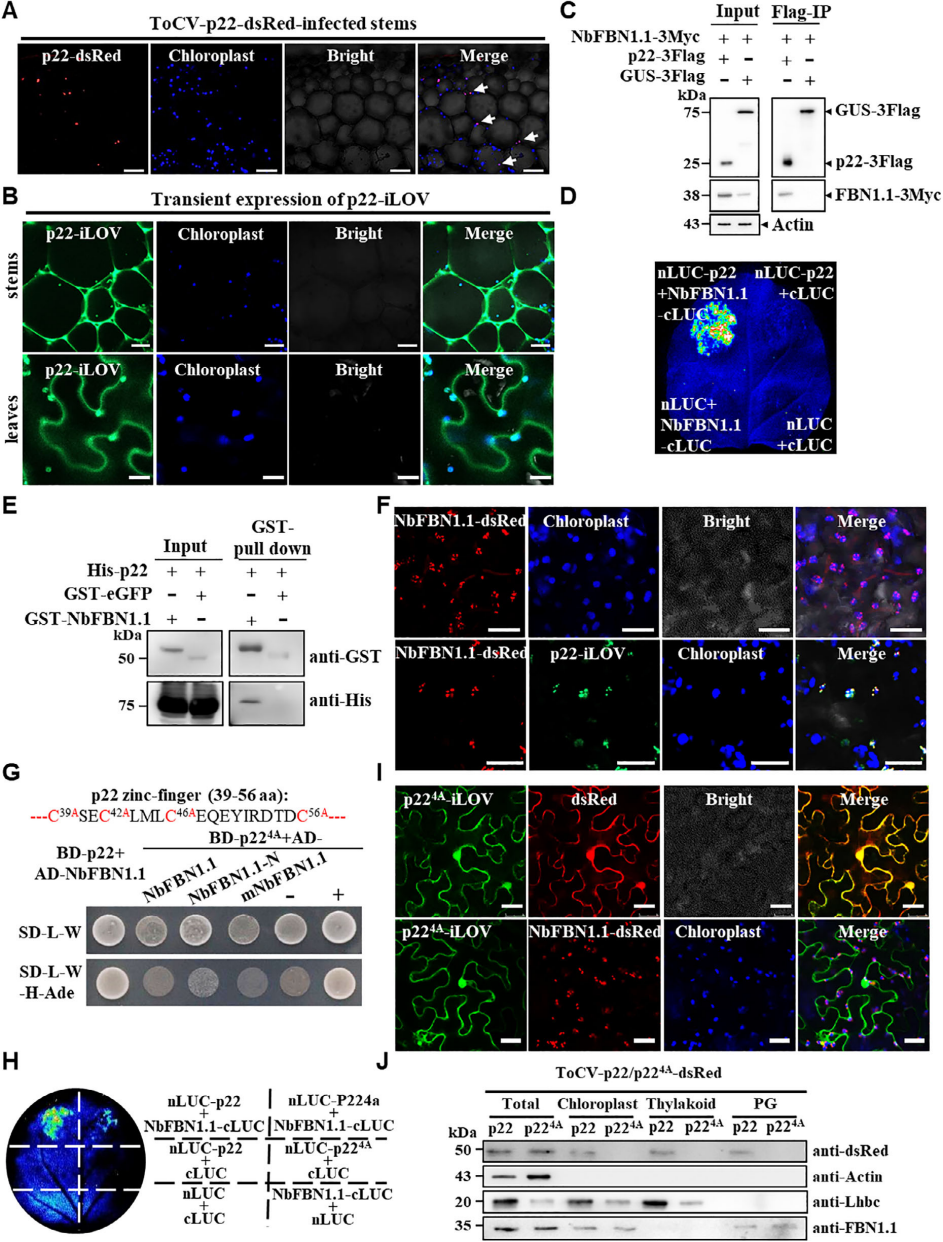
P22 of ToCV is capable of localizing within the chloroplast plastoglobules (PGs) via binding to the structural protein fibrillin (NbFBN1.1) of PG. A) p22 co‐localizes with chloroplasts in the parenchyma cells of the stem during infection of ToCV‐p22‐dsRed. White arrows indicate the p22 protein co‐localizing with chloroplasts. Bars = 50 µm. B) Subcellular localization of single transient expression of p22‐iLOV in the stem parenchyma cells (the upper panel) and the leaf epidermal cells (the lower panel) of *N. benthamiana*. Bars = 100 µm (upper), and 25 µm (lower). C) Co‐IP assays showed that p22 interacts with NbFBN1.1 in vivo. D) LCI assays showed a positive interaction between p22 and NbFBN1.1. E) GST pull‐down assays showed that p22 directly binds to NbFBN1.1 in vitro. F) Subcellular localization of single transient expression of NbFBN1.1‐dsRed, and co‐expression of p22‐iLOV and NbFBN1.1‐dsRed in the epidermal cells of *N. benthamiana*. Confocal images were taken at 3 days post‐infiltration. Bars = 25 µm. G) The amino acid sequence of the ToCV p22 zinc‐finger motif. The red marks indicate that the four cysteines (C) are mutated to alanine (A). Yeast two‐hybrid assays showed that p22^4A^ did not interact with NbFBN1.1 and mNbFBN1.1. H) LCI analysis showed that p22^4A^ impairs the interaction with NbFBN1.1. I) Confocal analysis of p22^4A^‐iLOV subcellular localization when co‐expressed with dsRed or NbFBN1.1‐dsRed in *N. benthamiana* epidermal cells. Bars = 50 µm. J) Immunoblotting assay determined the enrichment of p22 under ToCV‐p22‐dsRed or ToCV‐p22^4A^‐dsRed infection. Chloroplasts, thylakoids, and PGs were isolated and confirmed by extraction of protein and immunoblotting using anti‐Actin (cytoplasmic marker), anti‐Lhbc (thylakoid marker), and anti‐FBN1.1 (PG marker) antibodies.

To explore how p22 regulates chloroplast oxidative stress, we investigated the subcellular localization of p22 during ToCV infection. Since ToCV infection is phloem‐limited, we focused on cells of infected stem tissues. Strikingly, during infection of ToCV‐p22‐dsRed, the red fluorescence of p22‐dsRed fusion protein predominantly formed small punctate structures within chloroplasts of stem parenchyma cells (**Figure** **3**A; Figure S4, Supporting Information). Additionally, we employed two distinct methods for transient expressing p22 fused with an iLOV tag (p22‐iLov). One is a SWCNTs‐based pDNA delivery system in *N. benthamiana* stems,^[^ ^37^ ^]^ and another is agrobacterium‐mediated transient expression in *N. benthamiana* leaves. The results confirmed that p22‐iLOV localized to both the cytoplasm and chloroplasts of stem parenchyma cells, as well as leaf epidermal cells (Figure 3B).

Then, we unravel the way of p22 accessing chloroplasts. Absence of a chloroplast‐targeting signal peptide in p22 suggests that it employs an alternative, non‐canonical pathway for chloroplast entry. Our previous work preliminarily identified a tomato plastid lipid‐associated protein fibrillin (FBN, NM_001247254.1), which serves as a structural component of PG,^[^ ^20^ ^]^ interacting with p22 in a yeast library screening.^[^ ^27^ ^]^ Notably, unlike *N. benthamiana*, ToCV has limited success in infecting tomato plants via agro‐infiltration,^[^ ^38^, ^39^ ^]^ and both belong to the *Solanaceae* family; therefore, we decided to determine whether the FBN protein from *N. benthamiana* could interact with p22. Through bioinformatics analysis (http://solgenomics.net), we found *fibrillin family protein* (Niben101Scf09217g01001.1) has the highest homology with tomato FBN. In *Arabidopsis*, seven FBNs (FBN1a, −1b, −2, −4, −7a, −7b, and −8) were considered as PG core proteins.^[^ ^40^ ^]^ Furthermore, 13 putative PG‐localized NbFBNs homologues were identified in the *N. benthamiana* genome through bioinformatics analysis (http://solgenomics.net). Then, an evolutionary tree was constructed for the 13 NbFBNs and 7 FBNs of *Arabidopsis*, and NbFBNs were named based on their homology (Figure S5, Supporting Information). Among them, NbFBN1.1 (Niben101Scf09217g01001.1) has the highest homology with *Arabidopsis* FBN1a (AT4G04020.1) and FBN1b (AT4G22240.1). The results of quantitative reverse transcription‐PCR (RT‐qPCR) showed that the altered expression level of *NbFBN1.1* was the highest following ToCV infection (Figure S6, Supporting Information). Then, yeast two‐hybrid (Y2H) assays showed that NbFBN1.1 strongly interacted with p22, while, the other did not or only weakly interact with p22 (Figure S7, Supporting Information). Based on these results, we chose NbFBN1.1 for further analysis. Co‐immunoprecipitation (Co‐IP) assays demonstrated that NbFBN1.1 was specifically co‐immunoprecipitated with p22 in vivo, but not with the control protein GUS (Figure 3C). In the firefly luciferase complementation imaging (LCI) assay, robust luciferase activity was observed when nLUC‐p22 and NbFBN1.1‐cLUC were co‐expressed in *N. benthamiana* leaves (Figure 3D). Additionally, GST pull‐down assays further confirmed that His‐p22 could directly bind to GST‐NbFBN1.1, but not the control GST‐eGFP (Figure 3E). Consistent with the previous report,^[^ ^41^ ^]^ NbFBN1.1‐dsRed alone localized within the chloroplast (Figure 3F, the upper panel). Intriguingly, when co‐expressing p22‐iLOV and NbFBN1.1‐dsRed together, a well‐merged fluorescent signal was observed within chloroplasts (Figure 3F, the lower panel). Furthermore, the immunogold labeling experiment confirmed that p22‐dsRed and NbFBN1.1 were spatially proximal to each other and localized within chloroplast PG during infection of ToCV‐p22‐dsRed (Figure S8, Supporting Information).

To ascertain whether the binding of p22 to NbFBN1.1 dictates the localization of p22 within chloroplast PG, we identified the critical residues of p22 for interacting with NbFBN1.1. ToCV p22 harbors a zinc finger domain (amino acids 39–56), which encompasses four cysteines (C^39^, C^42^, C^46^, and C^56^).^[^ ^42^ ^]^ We mutated all four cysteines to alanine (A), resulting in the mutant named p22^4A^. The p22^4A^ mutant exhibited compromised interaction with NbFBN1.1 both in vitro and vivo (Figure 3G,H). Notably, when co‐expressed with NbFBN1.1‐dsRed, p22^4A^‐eGFP remained in the cytoplasm and nucleus, failing to access PG (Figure 3I). Furthermore, to determine the subcellular localization of p22 and its mutant p22^4A^ during ToCV infection, chloroplasts, thylakoids, and PGs were isolated from *N. benthamiana* plants infected by ToCV‐p22‐dsRed or ToCV‐p22^4A^‐dsRed. Immunoblotting analysis revealed enrichment of p22 in these compartments upon ToCV‐p22‐dsRed infection. In contrast, no accumulation of p22^4A^ was detected in chloroplasts, thylakoids, or PGs following ToCV‐p22^4A^‐dsRed infection (Figure 3J). Moreover, we also determined the enrichment of p22 in PGs of transgenic 35S:p22 L2 and L3 plants (Figure S9, Supporting Information).

The domain of NbFBN1.1 responsible for binding with p22 was mapped. The N‐terminal 55 amino acids were identified as the chloroplast signal peptide of NbFBN1.1 (Figure S10, Supporting Information). Subsequently, our analysis revealed that the mature domain of NbFBN1.1 (designated as mNbFBN1.1) is essential for binding with p22, as confirmed through Y2H, Co‐IP, and LCI assays (Figure S11A–D, Supporting Information). Consistently, the p22^4A^ mutant exhibited compromised interaction with mNbFBN1.1 (Figure 3H). When co‐expressing p22‐iLOV and mNbFBN1.1‐dsRed, merged fluorescence was exclusively observed in the cytoplasm (Figure S11E, Supporting Information). These results corroborate that the localization of p22 within PG is contingent on its binding with the mature domain of NbFBN1.1.

### 2.4 NbFBN1.1 Plays a Pivotal Role in Regulating Chloroplast Oxidative Stress

**Figure 4:**
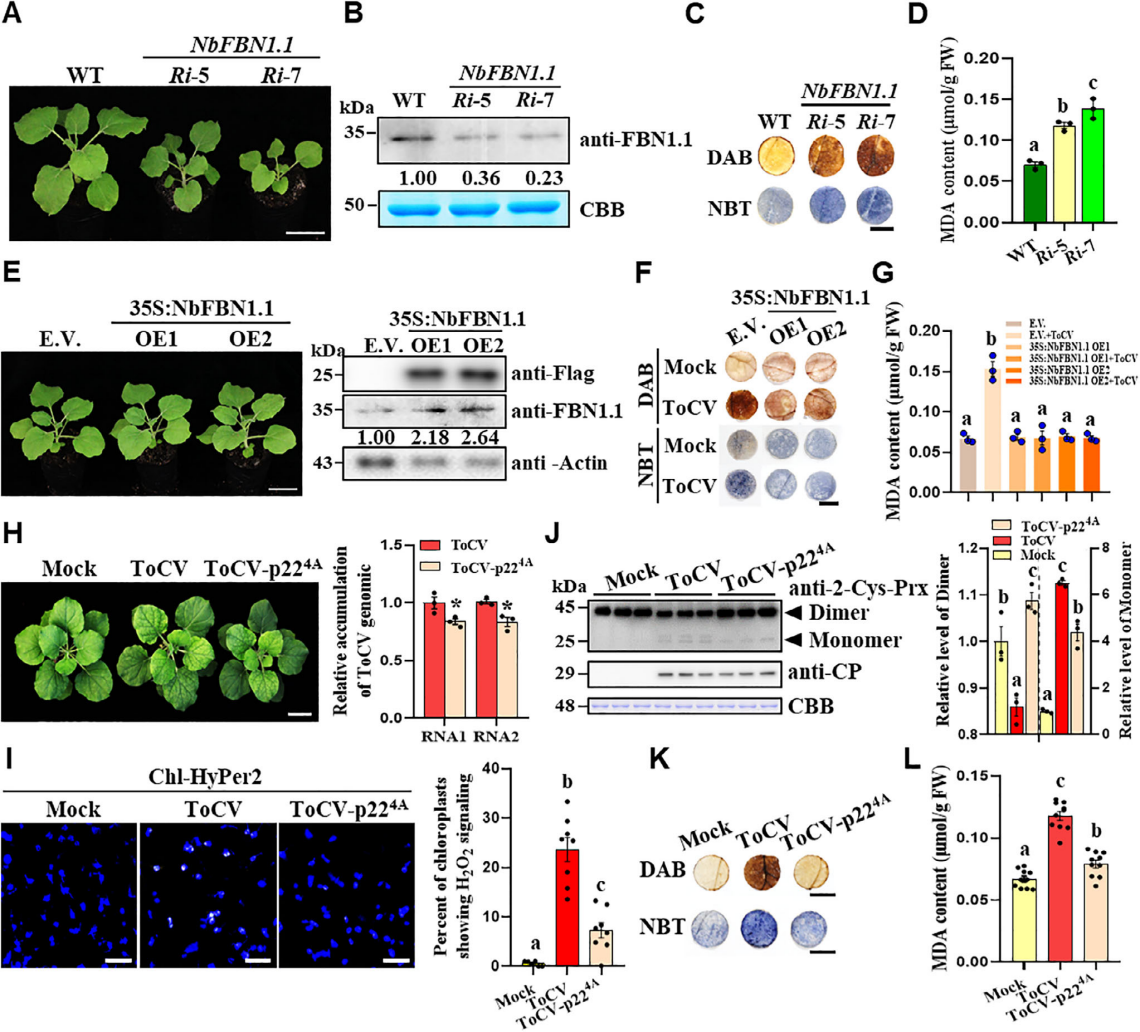
NbFBN1.1 plays a critical role in regulating oxidative stress in chloroplast PG. A) Two *NbFBN1.1*‐RNAi transgenic *N. benthamiana* lines (e.g., *Ri*‐5 and *Ri*‐7) are stunted with shortened stem internodes. Bar = 5 cm. B) Immunoblotting assay determined the accumulation levels of NbFBN1.1 in WT and *NbFBN1.1*‐RNAi plants. C) Histochemical staining of leaf discs from *NbFBN1.1*‐RNAi and WT plants using DAB for H_2_O_2_ production and NBT for O_2_^−^ production. Bar = 1 cm. D) MDA content in these upper leaf samples was analyzed. E) NbFBN1.1 over‐expression transgenic lines (e.g., OE1 and OE2) show similar growth phenotypes as the E.V. control plants. Bar = 5 cm. Expression of NbFBN1.1 is confirmed using anti‐Flag and anti‐NbFBN1.1 antibodies. The levels of actin in these samples were used to show sample loadings. F) Histochemical staining of leaf discs from the above plants using DAB for H_2_O_2_ production and NBT for O_2_^−^ production. Bar = 1 cm. G) The MDA contents in these upper leaf samples were analyzed. H) The symptoms and viral RNA levels in ToCV‐ and ToCV‐p22^4A^‐infected plants. Photographs were taken at 21 dpi. Bar = 5 cm. Asterisks indicate statistically significant differences between treatments. *, *p *< 0.05; determined using the two‐tailed Student's *t*‐test. I) Chl‐HyPer2 response to chloroplast H_2_O_2_ elicited by ToCV or ToCV‐p22^4A^ infection. Bars = 25 µm. The right panel shows the quantification of changes in Chl‐HyPer2 fluorescence. J) Immunoblotting results show the 2‐Cys Prx redox status in assayed leaves. The levels of 2‐Cys‐Prx Dimer were taken to be 1. K) Histochemical staining of leaf discs from the above plants using DAB for H_2_O_2_ production and NBT for O_2_^−^ production. Bars = 1 cm. L) Analysis of MDA content in the above plant leaves. The CBB‐stained rubisco large subunit gel in (B,J) was used to show sample loadings. Intensities of the protein bands in (B,E,J) were determined using the ImageJ software. Different letters above the bars in (D,G,I,J,L) indicate the statistically significant differences between treatments, determined using one‐way ANOVA followed by Tukey's multiple test (*p* < 0.05). Error bars were SEM. These experiments were performed three times with similar results.

To elucidate the role of NbFBN1.1 in modulating oxidative stress, we endeavored to knockout the expression of *NbFBN1.1* in *N. benthamiana* using CRISPR/Cas9‐based gene editing technology. However, these CRISPR/Cas9‐generated plants exhibited significant inhibition of seedling growth (Figure S12, Supporting Information). Alternatively, we generated two *NbFBN1.1*‐RNA interference (RNAi) lines. The *Ri*‐5 line plants displayed moderately stunted growth, whereas the *Ri*‐7 line plants exhibited severely stunted growth. Immunoblotting confirmed the substantial downregulation of NbFBN1.1 accumulation in both RNAi lines (**Figure** **4**A,B). Consistently, the *Ri*‐5 and *Ri*‐7 plants exhibited significantly elevated levels of H_2_O_2,_ O_2_^−^, and MDA compared with WT plants (Figure 4C,D). Meanwhile, significantly decreased levels of Φ_PSII_, *q_P_*, and ETR were detected in both RNAi lines compared with WT plants (Figure S13, Supporting Information).

To further understand the function of NbFBN1.1, we generated NbFBN1.1‐overexpressing *N. benthamiana* transgenic lines, OE1 and OE2 (Figure 4E). Comparing with E.V. control plants, we observed no significant alterations in the levels of H_2_O_2,_ O_2_^−^, and MDA in OE1 and OE2 lines (Figure 4F,G). Subsequently, we employed OE1 and OE2 lines to investigate the effect of ToCV on oxidative stress levels. The results showed that ToCV infection exerted a lesser impact on ROS levels, lipid oxidation, and photosynthesis in OE1 and OE2 plants compare with E.V. controls (Figure 4F,G; Figure S14, Supporting Information).

Intriguingly, the sizes of PG derived from OE1 and OE2 lines were significantly larger than those observed in E.V. plants (Figure S15, Supporting Information). Moreover, PG isolated from ToCV‐infected or p22‐transgenic expression plants was smaller than those of E.V. plants (Figure S16, Supporting Information). These results suggest a potential mechanism by which p22 may modulate the function of PG by modulating their size.

To ascertain whether the elevated chloroplast lipid oxidation caused by ToCV infection was a direct consequence of p22 binding to NbFBN1.1, we created a mutant virus, ToCV‐p22^4A^, which expresses a p22 mutant, p22^4A^, that compromised binding to NbFBN1.1. ToCV‐p22^4A^ was capable of infecting *N. benthamiana*, whereas it caused less severe chlorosis symptoms and accumulated lower levels of genomic RNAs compared with wild‐type ToCV (Figure 4H). Although the photosynthetic and oxidative parameters did not completely recover to healthy‐plant levels, notably, the ToCV‐p22^4A^‐infected plants exhibited significantly reduced chloroplast oxidative stress compared with ToCV‐infected plants (Figure 4I,J). Moreover, the levels of H_2_O_2_, O_2_^−^, and MDA were decreased in ToCV‐p22^4A^‐infected plants, while the values of *Fv*/*Fm*, Φ_PSII_, *q_P_*, and ETR were significantly higher than those in ToCV‐infected plants (Figure 4K,L; Figure S17, Supporting Information). Additionally, we confirmed that p22 did not diminish the expression levels of either NbFBN1.1 or mNbFBN1.1 (Figure S18, Supporting Information). Collectively, these results indicate that NbFBN1.1 plays a pivotal role in regulating oxidative stress, and ToCV promotes lipid oxidation by targeting to NbFBN1.1 via p22.

### 2.5 P22 Competes with NbVTE1 for Binding to NbFBN1.1, Interfering Biosynthesis of α‐Tocopherol

**Figure 5:**
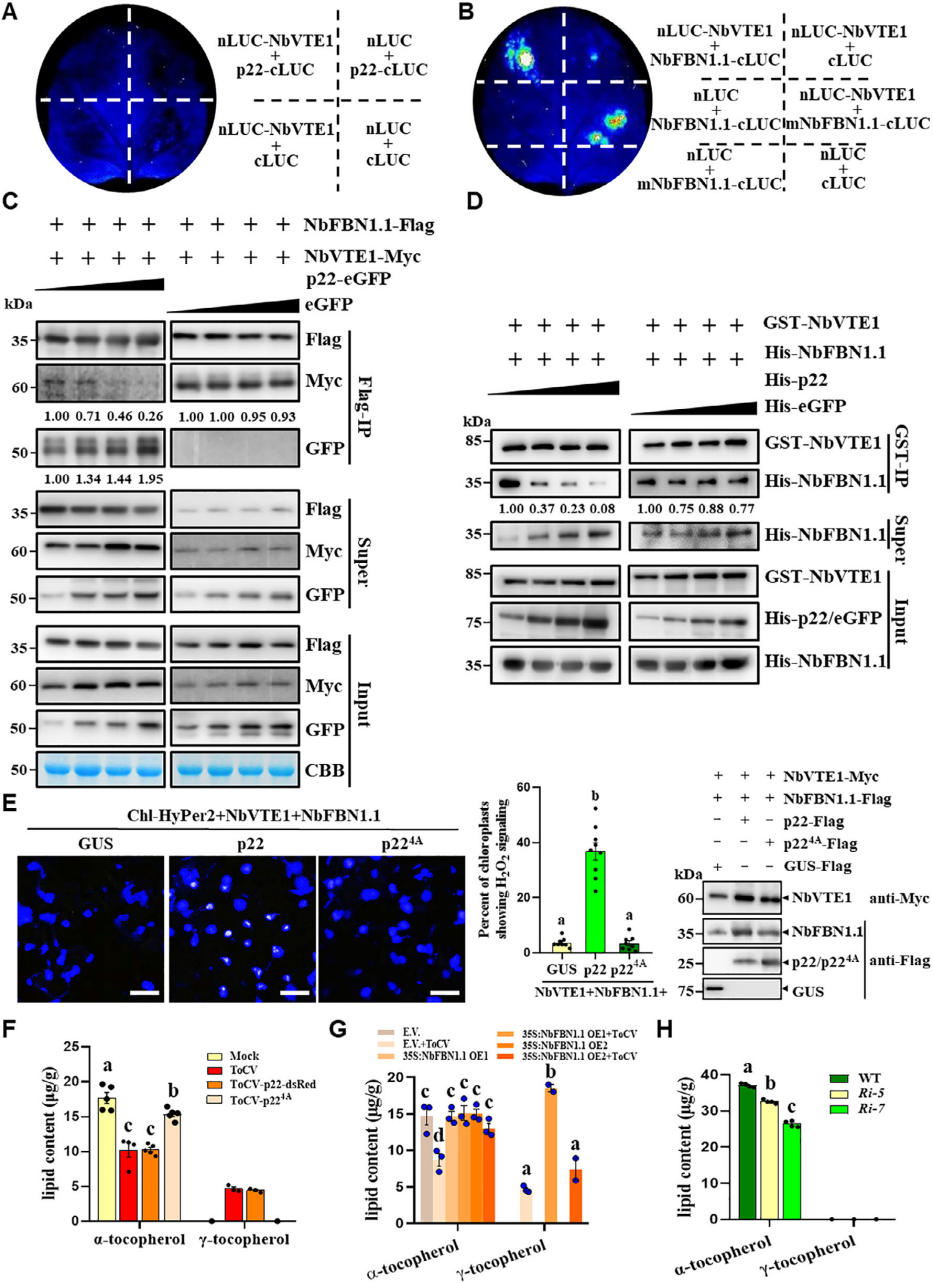
P22 competes with NbVTE1 for binding to NbFBN1.1 to suppress the biosynthesis of α‐tocopherol. A) The LCI assay showed that p22 does not interact with NbVTE1. B) The LCI assay showed that NbVTE1 interacts with the mature domain of NbFBN1.1 (mNbFBN1.1). C) The dose‐dependent effect of ToCV p22 on interference with the NbVTE1‐NbFBN1.1 interaction in the Co‐IP assay. The CBB‐stained gel was used to show sample loadings. D) GST pull‐down results indicated that ToCV p22 interferes with the interaction between NbVTE1 and NbFBN1.1 in a dose‐dependent manner. The intensities of the protein bands in (C,D) were determined using ImageJ software. E) Chl‐HyPer2 response to chloroplast H_2_O_2_ elicited by p22 or p22^4A^. GUS, p22 or p22^4A^ were coexpressed with Chl‐HyPer2, NbVTE1, and NbFBN1.1 in ToCV‐infected *N. benthamiana* leaves. Bars = 25 µm. The right panel shows the quantification of the ToCV‐dependent changes in Chl‐HyPer2 fluorescence. Immunoblotting results showed that the proteins were expressed successfully. F‐H) HPLC analysis of the accumulation level of α‐tocopherol and γ‐tocopherol in upper leaves from mock‐, ToCV‐, ToCV‐p22‐dsRed, or ToCV‐p22^4A^‐infected plants (F), mock‐ or ToCV‐infected E.V., NbFBN1.1‐OE1 and OE2 transgenic plants (G), and *NbFBN1.1* RNAi plants (H). Different letters above the bars in (E–H) indicate statistically significant differences between treatments, determined using one‐way ANOVA followed by Tukey's multiple test (*p* < 0.05); error bars are SEM. These experiments were repeated three times with similar results.

VTE1 is a committed enzyme responsible for the biosynthesis and recycling of α‐tocopherol within PG.^[^ ^19^ ^]^ To explore the potential mechanism by which p22 induces chloroplast lipid oxidation, we initially examined whether p22 or NbFBN1.1 could interact with NbVTE1. Through LCI and GST pull‐down assays, no interaction between p22 and NbVTE1 was observed (**Figure** **5**A; Figure S19, Supporting Information). Conversely, NbFBN1.1 could directly interact with NbVTE1, as demonstrated by LCI, Co‐IP, GST pull‐down, and Y2H assays (Figure 5B–D; Figure S20, Supporting Information). Moreover, mNbFBN1.1 was identified to be responsible for this interaction (Figure 5B). Additionally, confocal microscopic images exhibited colocalization of NbFBN1.1 and NbVTE1 within PG (Figure S21, Supporting Information), supporting that NbFBN1.1 positively regulates α‐tocopherol biosynthesis via binding with NbVTE1.

To investigate whether p22 competes with NbVTE1 to bind with NbFBN1.1, we conducted competitive Co‐IP and GST pull‐down assays. As the amount of p22 increased in the reactions, a notable decrease in the amount of NbVTE1 was pulled down by NbFBN1.1 (Figure 5C,D), indicating that p22 competes with NbVTE1 for binding to NbFBN1.1. To further explore whether this disruption of NbVTE1‐NbFBN1.1 binding by p22 affects chloroplast ROS scavenging by α‐tocopherol, we co‐expressed Chl‐HyPer2, NbVTE1, and NbFBN1.1 with p22, p22^4A^, or GUS (a control) in ToCV‐infected upper leaves. The results showed that when co‐expressing NbVTE1 and NbFBN1.1 with p22, ≈40% of chloroplasts showed detectable H_2_O_2_ signals, whereas co‐expressing with p22^4A^ or GUS only resulted in 3% to 5% of chloroplasts with detectable H_2_O_2_ signals (Figure 5E). In addition, the accumulation level of α‐tocopherol was significantly reduced by ≈40%, while the accumulation level of γ‐tocopherol (the precursor of α‐tocopherol) increased in ToCV‐ and ToCV‐p22‐dsRed‐infected plants compared with ToCV‐p22^4A^‐infected and mock plants (Figure 5F). Furthermore, we found no significant change in α‐tocopherol accumulation levels in NbFBN1.1‐overexpressing OE1 and OE2 lines following ToCV infection compared with mock control (Figure 5G). Consistently, the α‐tocopherol content was significantly reduced in *NbFBN1.1*‐RNAi lines (Figure 5H). These results confirm that NbFBN1.1 plays a crucial role in promoting α‐tocopherol biosynthesis. Taken together, p22 potentially inhibits α‐tocopherol‐mediated chloroplast ROS scavenging by disrupting NbVTE1‐NbFBN1.1 binding.

### 2.6 NbVTE1 Enhances Resistance to ToCV Infection by Facilitating the Accumulation of α‐Tocopherol

To determine the role of α‐tocopherol in ToCV infection, we silenced *NbVTE1* expression using a TRV‐based VIGS vector (TRV‐*NbVTE1*). The *NbVTE1*‐silenced plants displayed growth retardation and shorter stem internodes compared with the control (TRV‐*mCherry*) (Figure S22A,B, Supporting Information). Consistent with our expectations, silencing *NbVTE1* resulted in decreased levels of α‐tocopherol while increased levels of H_2_O_2_, O_2_^−^, and MDA compared with control (Figure S22C, Supporting Information). Notably, while the value of *Fv*/*Fm* remained unchanged in *NbVTE1*‐silenced plants, the values of Φ_PSII_, *q_P_* and ETR were significantly decreased (Figure S22D, Supporting Information).

**Figure 6:**
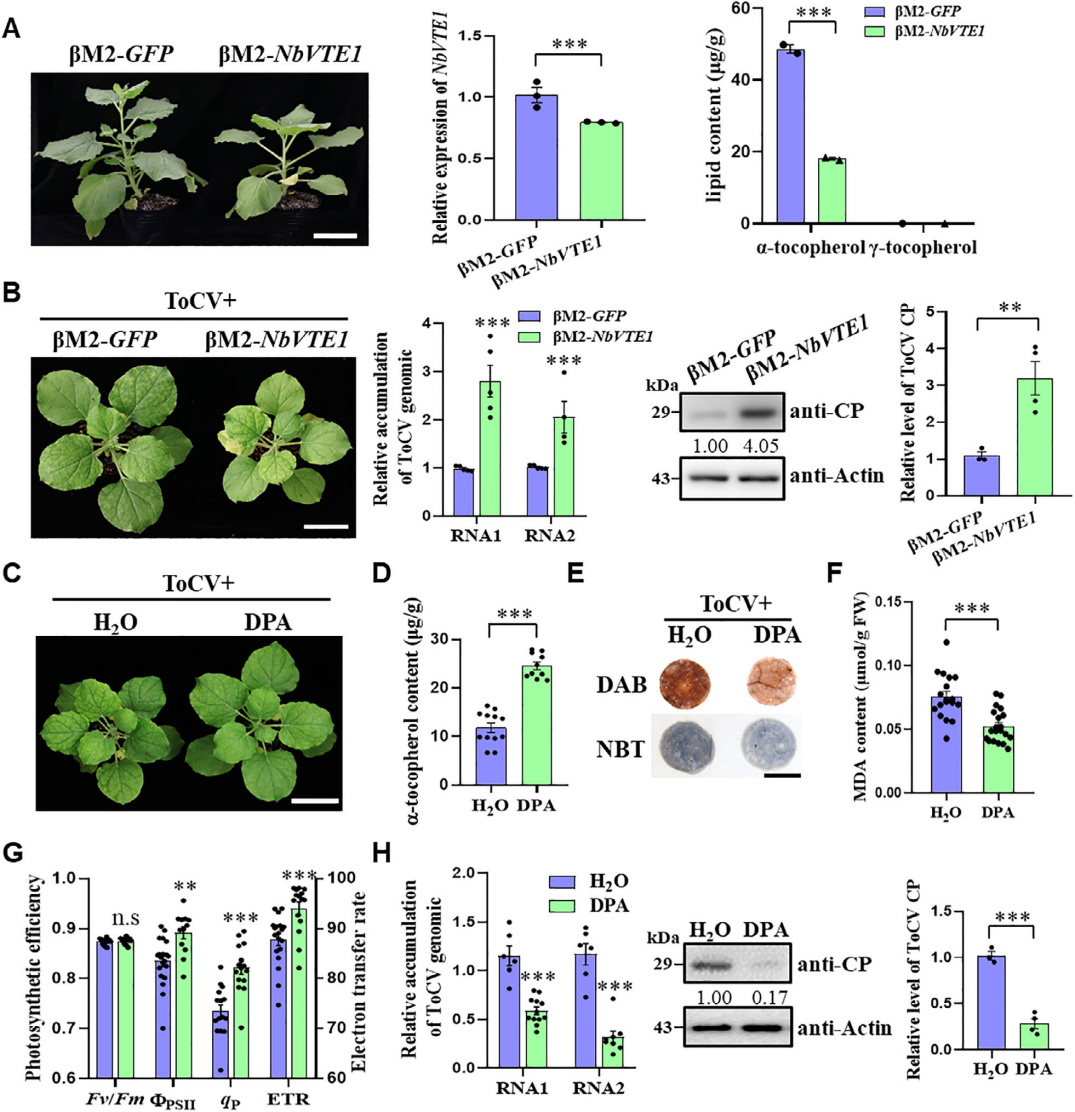
VTE1‐mediated α‐tocopherol biosynthesis confers resistance to ToCV infection. A) Silencing *NbVTE1* in *N. benthamiana* plants using a βM2‐based VIGS vector. The *NbVTE1*‐silencing plants showed a moderate stunted phenotype. Bar = 5 cm. RT‐qPCR confirmed that *NbVTE1* was downregulated in βM2‐*NbVTE1*‐inoculated plants compared to the control (βM2*‐GFP*). HPLC analysis showed that the content of α‐tocopherol in the *NbVTE1*‐silenced plants was significantly reduced. B) Silencing *NbVTE1* in *N. benthamiana* plants (βM2‐*NbVTE1*) enhanced ToCV infection‐induced chlorosis and viral accumulation compared to that in control plants (βM2‐*GFP*). The assayed plants were photographed at 21 dpi of ToCV. Bar = 5 cm. RT‐qPCR analysis showed the accumulation levels of ToCV RNA1 and RNA2 in *NbVTE1*‐silenced and control plants. Immunoblotting analysis showed ToCV CP levels in the *NbVTE1*‐silenced and control plants. The relative densities of each band were analyzed using ImageJ software, and the level of ToCV CP in βM2‐*GFP* plants was taken to be 1. C) Application of exogenous DPA suppressed ToCV infection. Following ToCV infection, DPA‐treated plants developed milder chlorosis compared with H_2_O‐treated plants. The assayed plants were photographed at 21 dpi of ToCV. Bar = 5 cm. D) HPLC analysis of the content of α‐tocopherol in the DPA‐ and H_2_O‐treated plants. E) Histochemical staining of leaf discs using DAB for H_2_O_2_ production and NBT for O_2_^−^ production. Bar = 1 cm. F) Analysis of MDA content following DPA or H_2_O treatment in ToCV‐infected plants. G) Comparison of the values of *Fv*/*Fm*, *Φ*_PSII_, *q*_P_, and ETR in the above plants. H) Relative accumulation levels of ToCV RNA1 and RNA2 and CP determined by RT‐qPCR or immunoblotting in DPA‐ and H_2_O‐treated plants. The relative densities of each band were analyzed using ImageJ software, and the level of ToCV CP in H_2_O‐treated control plants was taken to be 1. Asterisks above the bars in (A,B,D,F–H) indicate statistically significant differences between the treatments (n.s, not significant; **, *p *< 0.01; ***, *p *< 0.001), determined using the two‐tailed Student's *t‐*test, error bars were SEM. These experiments were performed three times, and at least six biological replicates were used per treatment.

Given the severe growth‐deficient phenotypes observed in TRV‐*NbVTE1* plants, we opted to utilize a cotton leaf curl Multan betasatellite‐based VIGS vector (βM2)^[^ ^43^ ^]^ to silence *NbVTE1*. The βM2‐*NbVTE1* plants also exhibited growth defects and decreased α‐tocopherol content compared with control plants (βM2‐*GFP*) (**Figure** **6**A). Following ToCV infection, the βM2‐*NbVTE1* plants showed more pronounced chlorosis and significantly increased levels of ToCV genomic RNAs and CP than that of the control (Figure 6B).

Diphenylamine (DPA) is known to maintain α‐tocopherol content in plants by scavenging O_2_^−^.^[^ ^44^ ^]^ To further investigate the role of α‐tocopherol in ToCV infection, we sprayed *N. benthamiana* plants with DPA or H_2_O (control) and then inoculated them with ToCV. The DPA‐sprayed plants showed milder chlorosis compared with the control (Figure 6C). Notably, the α‐tocopherol content in DPA‐sprayed plants was twice as high as that in the control (Figure 6D). Consistently, the levels of H_2_O_2_, O_2_^−^, and MDA were decreased, while the values of *Φ*_PSII_, *q_P_*, and ETR were significantly higher in DPA‐sprayed plants following ToCV infection (Figure 6E–G). Additionally, the accumulation levels of ToCV genomic RNAs and CP were significantly decreased in DPA‐sprayed plants compared with the control (Figure 6H). These results demonstrate that α‐tocopherol confers resistance to ToCV infection.

### 2.7 FBN1.1 Plays a Dual Role in Modulating Both Antiviral Resistance and Drought Resistance

**Figure 7:**
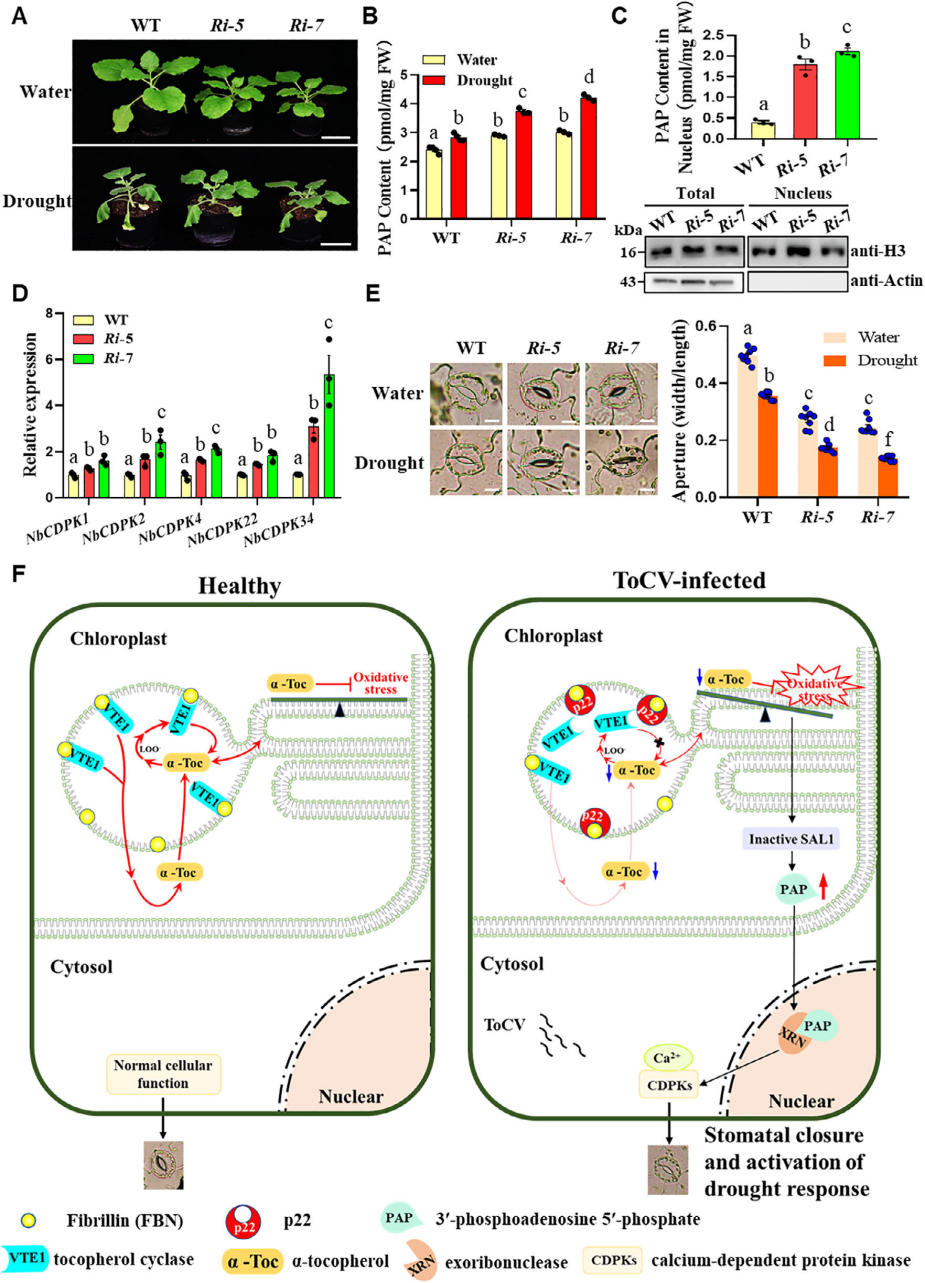
Knockdown expression of *NbFBN1.1* stimulates SAL1‐PAP retrograde signal and improves plant drought tolerance. A) Phenotypes of WT and *NbFBN1.1* RNAi plants under water and drought conditions. Bars = 5 cm. B) PAP levels in wild type and two lines of *NbFBN1.1* RNAi (*Ri‐5*, *Ri‐7*) plant leaves. C) The PAP accumulation levels in the nucleus were significantly up‐regulated in *NbFBN1.1* RNAi plants. Nuclear protein was extracted and analyzed by immunoblotting using anti‐H3 (nuclear marker) and anti‐Actin (cytoplasmic marker) antibodies. D) The transcriptional levels of *NbCDPKs* in WT and *NbFBN1.1* RNAi plants under drought conditions. E) Representative stomatal images and stomatal apertures of WT, and *NbFBN1.1* RNAi (*Ri‐5*, *Ri‐7*) plants under normal and drought conditions. Bars = 10 µm. Different letters above the bars in (B–E) indicate the statistically significant differences between the treatments, determined by a one‐way ANOVA test followed by Tukey's multiple test (*p* < 0.05). Error bars were SEM. These experiments were performed two times and had six biological replicates per treatment. F) A proposed working model for the function of ToCV‐encoded p22 in interfering with the biosynthesis of α‐tocopherol and enhancing drought tolerance. In healthy plant cells, NbVTE1 binds with PG structural protein fibrillin (NbFBN1.1), which is crucial for both α‐tocopherol (α‐Toc) biosynthesis and recycling pathways to maintain normal photosynthesis and to balance the accumulation of ROS. In addition, the stomata remain open. Following ToCV infection, however, p22 translocates into chloroplast PG via binding with NbFBN1.1 and competes with NbVTE1 for NbFBN1.1. The biosynthesis of α‐Toc is interrupted, and the content of α‐Toc decreases, which benefits ToCV infection. The lower content of α‐Toc causes oxidative stress in the chloroplast, which stimulates SAL1‐PAP‐mediated retrograde signaling to activate the expression of drought‐responsive genes, including calcium‐dependent protein kinases (*CDPKs*), leading to stomatal closure and enhanced drought tolerance.

The above results indicate that ToCV infection or its encoded p22 protein enhances drought tolerance via stimulating SAL1‐PAP retrograde signaling. This process involves modulating the chloroplast ROS state through disrupting VTE1‐FBN1.1 binding. To further elucidate the role of NbFBN1.1 in SAL1‐PAP retrograde signaling, we conducted experiments on *NbFBN1.1* RNAi plants. These plants exhibited enhanced drought tolerance, accompanied by elevated levels of PAP in both total leaf tissue and nuclear fraction, as well as increased expression levels of *NbCDPKs* compared with WT plants (**Figure** **7**A–D). Simultaneously, the stomatal apertures were reduced in *NbFBN1.1* RNAi plants (Figure 7E). Moreover, the *NbVTE1*‐silenced plants also showed enhanced drought resistance, and the level of PAP was significantly increased, as well as increased expression levels of *NbCDPKs* compared with the control (βM2‐*GFP*) (Figure S23, Supporting Information).

It is known that PGs increased in size and number under the majority of abiotic stresses, such as light stress.^[^ ^45^, ^46^ ^]^ Moreover, PAP accumulation could be induced by high‐light (HL) stress.^[^ ^13^, ^14^, ^47^ ^]^ Therefore, we compared the drought tolerance from mock‐ or ToCV‐infected wild‐type *N. benthamiana* and transgenic 35S:p22 plants under HL stress. Mock‐, ToCV‐infected, and transgenic 35S:p22 *N. benthamiana* plants were exposed to 1200 µmol m^−2^ s^−1^ light for three days, and then were subjected to drought treatment. The results showed that the HL‐treated wild‐type *N. benthamiana* showed accelerated wilting (Figure S24A, Supporting Information). However, HL pretreatment slightly induced PAP accumulation in wild‐type plants (Figure S24B, Supporting Information). In contrast, ToCV‐infected and 35S:p22 lines maintained elevated PAP under HL, and their drought tolerance remained high (Figures S24,S25, Supporting Information).

Next, we examined the role of FBN1.1 in ToCV infection. Plants of transgenic overexpression of NbFBN1.1, from lines of NbFBN1.1‐OE1 and ‐OE2, exhibited much less chlorosis in systemically infected leaves following ToCV infection, accompanied by substantial decreased levels of ToCV CP and genomic RNAs compared with E.V. plants (Figure S26A, Supporting Information). In contrast, plants of knock‐down *NbFBN1.1* from lines *Ri*‐5 and *Ri*‐7 displayed more severe chlorosis symptoms than WT plants following ToCV infection (Figure S26B, Supporting Information). Consistently, the accumulation levels of ToCV genomic RNAs and CP were significantly increased relative to WT plants (Figure S26B, Supporting Information). Furthermore, we utilized the βM2‐based VIGS vector to knock down *NbFBN1.1* expression. As expected, *NbFBN1.1*‐silenced plants (βM2‐*NbFBN1.1*) also developed more pronounced chlorosis and significantly elevated accumulation levels of ToCV genomic RNAs and CP compared with control (βM2‐*GFP*) (Figure S27, Supporting Information). Collectively, our results revealed that a low level of NbFBN1.1 leads to suppressed α‐tocopherol biosynthesis, which enhances drought tolerance but paradoxically favors ToCV infection.

## 3 Discussion

In this study, we uncovered that infection of ToCV disrupts the biosynthesis of α‐tocopherol in PG, leading to oxidative stress in chloroplasts (Figures 3, 5). The accumulation of ROS was detected by SAL1, which then activated SAL1‐PAP‐dependent chloroplast retrograde signaling. This activation led to an increase in the expression of *CDPKs*, promoting stomatal closure and enhancing the host's drought tolerance (Figure 1). Molecular and genetic evidence indicate that ToCV employs its encoded p22 protein to exploit FBN1.1 to access chloroplast PG, where it disrupts the FBN1.1‐VTE1 binding and thereby may interfere with the normal function of VTE1, and lead to the inhibition of α‐tocopherol biosynthesis (Figures 3, 5). Furthermore, we confirmed that α‐tocopherol exhibits antiviral function, potentially by mitigating oxidative stress in chloroplasts (Figure 6). Altogether, these findings offer a foundational theoretical framework for understanding how virus infections confer cross‐tolerance to abiotic stresses in the host via stimulating chloroplast retrograde signaling.

Since the discovery that virus‐infected plants can exhibit enhanced drought resilience,^[^ ^2^, ^5^, ^7^, ^48^ ^]^ extensive research has been dedicated to unraveling the underlying signaling pathways. So far, ABA‐dependent or ABA‐independent pathways, autophagy, and SA signaling have been implicated in virus‐induced drought tolerance.^[^ ^7^, ^48^ ^]^ In this study, we uncovered that ToCV infection triggers chloroplast ROS accumulation, stimulating SAL1‐PAP‐dependent chloroplast retrograde signaling, leading to improved drought tolerance. Moreover, our findings are corroborated by the elevated PAP content following ToCV infection or p22‐transgenic expression. Additionally, spraying an inhibitor of chloroplast retrograde signaling, NF, resulted in decreased expression levels of *CDPKs* in ToCV‐infected and p22‐transgenic plants. These results provide solid evidence for understanding how ToCV infection confers cross‐tolerance to abiotic stresses in hosts by stimulating chloroplast retrograde signaling. Given that SAL1 functions as a universal sensor of ROS,^[^ ^14^ ^]^ it would be intriguing to explore whether other viruses that induce ROS accumulation in chloroplasts can also activate the SAL1‐PAP pathway to bolster host drought tolerance. Research have been reported that HL‐stress could induce the accumulation of PAP, which activated the metabolite signal moves between the chloroplast, cytosol, and nucleus.^[^ ^13^, ^14^, ^47^ ^]^ However, HL‐treated wild‐type *N. benthamiana* showed accelerated wilting (Figure S24, Supporting Information). Besides, ToCV‐infected and 35S:p22 plants maintained elevated PAP under HL, and their drought tolerance remained high (Figures S24,S25, Supporting Information). These might due to the combination of high‐light and drought stresses altering the normal homeostasis of cells^[^ ^49^, ^50^ ^]^ and under these dual conditions, endogenous PAP accumulation is insufficient to compensate for severe stress. However, the interactions of plants and viruses improved their responses to abiotic stresses.

Chloroplasts, being pivotal organelles in plant defense, are frequently targeted by viral proteins through diverse strategies.^[^ ^26^ ^]^ For example, cucumber necrosis virus‐encoded CP and alternanthera mosaic virus‐encoded TGB3 can transport into chloroplasts via their unique chloroplast transit peptides, thereby facilitating viral infection.^[^ ^51^, ^52^ ^]^ Moreover, viral proteins have demonstrated to co‐opt host chloroplast‐targeting proteins to reach chloroplasts. TYLCV‐encoded C4 redistributes to chloroplasts subsequently through interacting with the COAT PROTEIN COMPLEX I, and inhibits the synthesis of host defense signaling molecules.^[^ ^53^ ^]^ Similarly, pepper mild mottle virus‐encoded CP targets chloroplasts by interacting with chloroplast outer membrane protein 24 (OMP24), disrupting the defense mechanism mediated by OMP24 dimerization.^[^ ^54^ ^]^ Tobacco vein banding mosaic virus‐encoded NIb engages with ribosomal protein large subunit 1 in chloroplasts, reducing Beclin1‐mediated degradation of NIb and thereby enhancing viral infection.^[^ ^55^ ^]^ In this study, ToCV‐encoded p22 targets PG through directly binding to FBN1.1, promoting ToCV infection. Our findings underscore chloroplast PG as a pivotal target during viral infections.

PGs serve as dynamic micro‐compartments in chloroplasts, essential for chloroplast biogenesis, developmental transitions, and environmental responses.^[^ ^20^ ^]^ Changes in PG size reflect alterations in the biosynthesis and export of major PG metabolites, such as tocopherol. These metabolic shifts further contribute to reversible modifications in PG size and properties.^[^ ^20^ ^]^ Previously, an increased PG size has been reported following infection of sugarcane yellow leaf virus.^[^ ^56^ ^]^ In contrast, our study observed a decreased PG size upon ToCV infection or p22‐transgenic expression. We found that p22 modulates PG function by directly binding to the C‐terminal mature domain of FBN1.1. Since the C‐terminal mature domain of FBN1.1 is indispensable for maintaining the structure and stability of PG,^[^ ^57^ ^]^ this interaction might impair PG structure, resulting to reductions in both PG size and α‐tocopherol biosynthesis. The diminished PG size may also hinder the exchange of α‐tocopherol between thylakoid and PG, resulting in lipid oxidation and decreased photosynthetic efficiency. These discoveries underscore the importance of PG as a viral target to manipulate the chloroplast's oxidative microenvironment, which is required for creating a favorable cellular environment conducive to successful infection.

As the primary structural protein of PG, FBN1.1 plays a fundamental role in regulating plant development. In Arabidopsis, the homozygous FBN5 is essential for maintaining a normal growth rate, and the *fbn5‐1* mutations result in seedling lethality.^[^ ^58^ ^]^ However, we did not find homologous genes of FBN5 in the *N. benthamiana* genome, but considering that knockout of *NbFBN1.1* also led to significant inhibition of seedling growth (Figure S12, Supporting Information) and the *NbFBN1.1‐*RNAi plants exhibited a stunted phenotype (Figure 6A–D). Therefore, we speculated that NbFBN1.1 may have multiple functions, reinforcing the necessity of NbFBN1.1 for plant development. Overexpression of NbFBN1.1 could stabilize chloroplast oxidation homeostasis during ToCV infection (Figure 4E–G). Since the expression level of NbFBN1.1 positively regulates both PG size and α‐tocopherol content, NbFBN1.1 actively responds to both chloroplast oxidative stress and pathogen infection via modulating α‐tocopherol content. Furthermore, NbFBN1.1 directly binds to NbVTE1 in PG. The *NbFBN1.1*‐RNAi and *NbVTE1*‐silenced plants have lower α‐tocopherol levels and exhibit stunted phenotypes (Figure 5B–H). Based on these results, we propose that NbFBN1.1 is involved in regulating α‐tocopherol biosynthesis by anchoring NbVTE1 on PG, thereby stabilizing the enzyme activity of NbVTE1. Further investigation is needed to determine how NbFBN1.1 modulates the enzyme activity of NbVTE1 in vivo.

FBNs have been reported to suppress infections of fungi and bacteria.^[^ ^57^ ^]^ For example, overexpressing FBN (CHRC) in tomato enhances resistance to *Botrytis cinerea*.^[^ ^24^ ^]^ Arabidopsis *fib4* deletion mutants and *fib4* knockout mutant apple trees were more sensitive to *Pseudomonas syringae* and *Erwinia amylovora* infections, respectively.^[^ ^59^ ^]^ Until now, the underlying mechanisms by which FBNs play a positive role in biotic stress resistance remain unknown. In this study, with *NbFBN1.1* overexpression and RNAi lines, we verified that NbFBN1.1 plays an essential role in suppressing ToCV infection (Figures S26,S27, Supporting Information). Nevertheless, ToCV‐encoded p22 accesses PG via binding to NbFBN1.1, where it interferes with the NbFBN1.1‐NbVTE1 binding, leading to decreased α‐tocopherol content. In contrast, p22^4A^, which neither interacts with FBN1.1 nor targets PG, does not interfere with the NbFBN1.1‐NbVTE1 binding (Figure 5C,D). Additionally, infection of ToCV‐p22^4A^ mutant attenuated the reduction in α‐tocopherol content compared with wild‐type ToCV (Figure 5F). These findings suggest that FBN1‐mediated suppression of ToCV infection depends on α‐tocopherol content. To counteract this defense, ToCV employs its encoded p22 protein to competitively bind to NbFBN1.1 and disrupt the NbFBN1.1‐NbVTE1 binding, leading to reduced PG size and decreased α‐tocopherol biosynthesis.

The α‐tocopherol has been reported as a crucial protector for maintaining both chloroplast redox balance and thylakoid membrane integrity during plant growth and environmental stresses.^[^ ^17^, ^44^, ^60^ ^]^ Previous studies have shown that infections with either *P. syringae* or *B. cinerea* induced strong accumulation of γ‐tocopherol, which is the precursor of α‐tocopherol.^[^ ^61^, ^62^ ^]^ Similarly, ToCV infection also caused increases in γ‐tocopherol levels. However, unlike *P. syringae* or *B. cinerea* infections, which had no impact on α‐tocopherol accumulation, ToCV infection significantly decreased the α‐tocopherol content. This negative correlation was further confirmed through the silencing of *NbVTE1* and infection with ToCV‐p22^4A^. Moreover, DPA treatment to maintain α‐tocopherol levels significantly hindered ToCV replication. These results underscore the pivotal role of α‐tocopherol in defense against ToCV. While it remains unclear whether α‐tocopherol directly engages in antiviral defense or triggers plant defense pathways similar to plant hormones,^[^ ^20^, ^63^ ^]^ our research broadens the understanding of α‐tocopherol's function in plant antiviral defenses.

Recent reports have highlighted the dual functions of ROS in either resistance or facilitating the infection and replication of plant viruses. Consequently, viruses may manipulate ROS accumulation to create a conducive environment for multiplication.^[^ ^25^, ^64^, ^65^ ^]^ For example, red clover necrotic mosaic virus‐encoded p27 induces intracellular ROS accumulation by hijacking RBOHB, a crucial factor for robust viral RNA replication.^[^ ^64^ ^]^ Here, we discovered that ToCV‐encoded p22 suppresses the accumulation of α‐tocopherol, leading to chloroplast oxidative stress and ultimately facilitating viral infection. Therefore, whether chloroplast ROS accumulation serves as a defensive or protective factor depends on the intricate equilibrium between ROS generator/scavenger systems and its specific interaction with viruses. Further research is imperative to elucidate how the chloroplast oxidation microenvironment, induced by decreased α‐tocopherol levels, benefits ToCV replication.

In summary, this study uncovered that a prevalent virus worldwide, ToCV, modulates PG in chloroplasts to impair α‐tocopherol biosynthesis via targeting FBN1.1, leading to lipid oxidation, which is sensed by SAL1, triggering the SAL1‐PAP retrograde signaling pathway, thereby facilitating infection and improving host drought tolerance. A working model is proposed (Figure 7F). These findings significantly contribute to our understanding of the pivotal role of PG and their metabolites (α‐tocopherol) in the intricate interplay between hosts and pathogens, as well as in host cross‐tolerance mechanisms.

## 4 Experimental Section

### Plant Growth and Virus Inoculation

Tomato and *N. benthamiana* plants were grown in pots containing peat and vermiculite (3:1, w/w) inside a growth room maintained at 24 °C/22 °C (day/night), 16 h/8 h (light/dark) photoperiod, and 60% relative humidity. The two lines of p22‐transgenic *N. benthamiana* were previously described.^[^ ^27^ ^]^

Four‐week‐old *N. benthamiana* plants were used for transient gene expression assays. Five‐leaf‐stage *N. benthamiana* plants were used for ToCV‐BJ inoculation through agro‐infiltration as described.^[^ ^39^ ^]^

### Plasmid Construction

The ToCV derivatives used in this study were generated from the ToCV‐BJ infectious clone.^[^ ^39^ ^]^ The coding sequence of *dsRed* was amplified and introduced into the C‐terminus of the p22 open reading frame (ORF) in ToCV‐BJ RNA1 to generate ToR1‐p22‐dsRed. The four amino acid mutations within p22 (p22^4A^) were designed in the primer and then cloned into ToCV‐BJ RNA1 to replace p22 to produce ToR1‐p22^4A^.

To overexpress a 3 × Flag‐tagged NbFBN1.1 in *N. benthamiana*, the coding sequence of *NbFBN1.1* was cloned into the pCambia1305 vector^[^ ^66^ ^]^ to produce p1305‐NbFBN1.1. To produce CRISPR/Cas9‐based *NbFBN1.1* knockout *N. benthamiana* plants, two 19 bp guide RNAs (gRNAs) specific for *NbFBN1.1* were designed in the following online servers: CRISPR direct (http://crispr.dbcls.jp/) and CRISPR‐P 2.0 (http://crispr.hzau.edu.cn/CRISPR2/). These two gRNAs were cloned into the pHSE401 vector as described^[^ ^67^ ^]^ to produce pHSE401‐NbFBN1.1. To produce *NbFBN1.1* RNAi transgenic *N. benthamiana* plants, a fragment representing a partial sequence of *NbFBN1.1* (nucleotide position 156–455) was selected using the online server SGN‐VIGS (https://vigs.solgenomics.net/), amplified, and inserted, as an inverted repeat, into the RNAi vector pFGC5941 to produce pFGC5941‐NbFBN1.1.

For the CLCuMuB‐ or TRV‐based VIGS vector, the fragments representing specific partial sequences of *NbFBN1.1* (nt 156–455) and *NbVTE1* (nt 833–1031) were predicted using the online server SGN‐VIGS (https://vigs.solgenomics.net/) and individually amplified and inserted into the βM2 or TRV RNA2 vector to produce βM2‐*NbFBN1.1*, βM2‐*NbVTE1*, and TRV‐*NbVTE1*, respectively.

For transient expression and subcellular localization assays, the coding sequences of target fragments were individually amplified and cloned into a pGD‐based vector.^[^ ^68^ ^]^ For the LCI assay, the coding sequences of target fragments were individually PCR amplified and cloned into the p35S:nLUC or p35S:cLUC vector.^[^ ^69^ ^]^ For the GST pull‐down assay, full‐length target fragments were individually cloned into the pCold or pGEX vector.

The primers used in this study are listed in Table S1 (Supporting Information). All recombinant plasmids were sequenced from both directions prior to use.

### Generation of NbFBN1.1 Overexpression (OE), Knockout (KO), and RNAi Transgenic *N. benthamiana* Plants

*N. benthamiana NbFBN1.1* OE, KO, and RNAi transgenic lines were produced through agrobacterium‐mediated transformation using plasmids p1305‐NbFBN1.1, pHSE401‐NbFBN1.1, or pFGC5941‐NbFBN1.1, respectively. *N. benthamiana* leaf disc transformation was performed as described.^[^ ^27^ ^]^ The resulting *NbFBN1.1* OE (OE1 and OE2) and RNAi (*Ri*‐5 and *Ri*‐7) transgenic lines were further identified through RT‐qPCR and Western blotting analyses.

### Drought Stress Treatment and Stomatal Aperture Measurement

To evaluate drought stress tolerance, 4‐week‐old *N. benthamiana* plants were bottom‐watered for 2 days to saturate the soil and then moved to dry pallets in which water was withheld.^[^ ^2^ ^]^ At 5 days post drought treatment, leaves were harvested for stomatal aperture and gene expression measurement. Plants normally treated with water were used as controls.

The stomatal aperture was measured using ImageJ software.^[^ ^70^ ^]^ Two independent experiments were performed, and at least 60 stomata were measured in one experiment.

### Water Loss Analysis

The water loss rate was measured according to Xu et al.^[^ ^2^ ^]^ Briefly, all the leaves from plants were individually detached and laid with the adaxial side up in a petri dish. Then, the weight of the leaves was measured every half hour until 5 h after detachment. The water loss ratio for each plant was calculated by dividing the weight loss at each time point by its total water weight. Two independent experiments were performed, and at least 6 individual plants were measured in one experiment.

### Nuclear Fractionation

*N. benthamiana* leaf tissues (15 g per sample) were collected from mock, ToCV‐inoculated, or transgenic plants at 21 dpi, frozen in liquid nitrogen, and then homogenized for nucleus fractionation according to a previous report.^[^ ^71^ ^]^ Actin and histone H3 were used as cytoplasmic and nuclear markers, respectively.

### NF Treatment

NF (AccuStandard) was first dissolved in 100% methanol to 20 mm and then diluted to 100 µm in ddH_2_O with 0.1% Triton X‐100 immediately before use. The 100 µm NF solution or ddH_2_O with 0.1% Triton X‐100 (mock) was sprayed onto *N. benthamiana* plant leaves and irrigated the root. Twelve hours later, the plants were treated under drought conditions. The assayed plants were sampled at 5 days post‐drought treatment for further analysis.

### Measurement of Chlorophyll Fluorescence

In vivo chlorophyll fluorescence measurements were performed using fully expanded upper leaves from mock‐, ToCV‐ and ToCV‐dsRed‐infected *N. benthamiana* plants. The LI‐6400XT photosynthesis system (Li‐Cor Biosciences, Lincoln, NE) was equipped with a leaf chamber fluorometer (Li‐Cor Part No. 6400–40, enclosed leaf area: 2 cm^2^) as instructed by the manufacturer. The measurements were performed at ≈22 °C (leaf temperature), and the irradiance was a mixture of red (90%) and blue (10%) lights from LEDs.

### Analysis of ROS Accumulation and MDA Accumulation

The chloroplast ROS assays were performed as described previously.^[^ ^36^ ^]^ Chl‐HyPer2 was expressed in ToCV‐infected upper leaves or p22‐transgenic plant leaves and observed at 4 dpi. Ratiometric images (F488/405 nm) of fluorescence excitation at 488 and 405 nm show the oxidized state of chloroplast‐targeted HyPer2. The percentage of ROS‐positive chloroplasts was calculated as described.^[^ ^25^ ^]^

The redox forms of the 2‐Cys Prx protein were determined using non‐reducing SDS‒PAGE as described previously.^[^ ^25^ ^]^ The samples were collected from ToCV‐infected upper leaves at 21 dpi or p22‐transgenic plant leaves.

Accumulations of H_2_O_2_ and O_2_^−^ in assayed *N. benthamiana* leaves were analyzed through staining of leaf discs in 1 mg mL^−1^ diaminobenzidine (DAB) or 0.5 mg mL^−1^ nitro blue tetrazolium (NBT) solution (Sigma‐Aldrich, St. Louis, MO, USA) for 10 h followed by discoloration in ethanol for 10 min at 100 °C.

The MDA content was measured and used as an indicator of lipid peroxidation as described.^[^ ^62^ ^]^ Briefly, fresh leaf tissues were collected and homogenized in phosphate buffer (pH 7.8, 0.1 g mL^−1^), followed by 20 min of centrifugation at 4 °C and 10 000 *g*. The resulting supernatants were incubated with 0.6% (m/v) thiobarbituric acid for 10 min at 100 °C. The absorbance of each supernatant was recorded at 450, 532, and 600 nm.

### HPLC Analysis of Tocopherols and PAP

The α‐ and γ‐tocopherols were extracted as described.^[^ ^72^ ^]^ Quantification of tocopherol was performed using an HPLC‐DAD (diode array detector). The tocopherols were separated using a ZORBAX Eclipse Plus C18 column (4.6 mm × 100 mm, 3.5 µm particle size, PN: 959961‐902, Agilent, California, USA) at 30 °C and at a flow rate of 1 mL min^−1^. Sample injections (20 µL per injection) were isocratic elution for 7 min with a mobile phase of water–methanol (1:49, v/v). For quantification, the UV trace at 230 nm was used. Absorbance response factors for *λ*_max_ were calculated from calibration curves generated using the standards of tocopherol (α‐ and γ‐tocopherol, Sigma) compounds.

PAP was extracted and quantified as described.^[^ ^73^ ^]^ For HPLC‐DAD analysis, PAP was separated using a ZORBAX Eclipse Plus C18 column, and the UV trace at 254 nm was used for quantification. Absorbance response factors for *λ*_max_ were calculated from calibration curves generated using the standards of PAP (Sigma).

### Total RNA Isolation and RT‐qPCR

Total RNA isolation, first‐strand cDNA synthesis, and RT‐qPCR were performed as described.^[^ ^27^ ^]^ Primers for RT‐qPCR were designed using NCBI Primer‐BLAST software and are listed in Table S1 (Supporting Information). The expression level of *NbActin* was used as an internal control for RT‐qPCR analysis.

### Transient Gene Expression

For transient gene expression in lower epidermal cells of *N. benthamiana* leaves, *agrobacterium tumefaciens* GV3101 strains carrying specific constructs were grown in LB media overnight, centrifuged, and resuspended in infiltration buffer (10 mm MgCl_2_, 10 mm MES, pH 5.6, and 200 µm acetosyringone) until OD_600_ = 0.5. Equal volumes of cultures were mixed for infiltration. Cultures were then spot‐infiltrated into *N. benthamiana* leaves.

For transient gene expression in *N. benthamiana* stem parenchyma cells, the method according to Liu et al.^[^ ^37^ ^]^ was followed. In brief, the PEI‐ssDNA‐SWCNTs solution containing 200 ng PEI‐ssDNA‐SWCNTs was added into a 10 µg plasmid DNA dropwise, mixed by pipetting 10 times, and incubated at room temperature for 30 min for the formation of the pDNA‐PEI‐ssDNA‐SWCNTs complex. After incubation, an appropriate amount of Silweet‐77 was added to make the final concentration 0.05%. The tender stems (cut into 1–2 cm rectangles) were added to the above solution and incubated at room temperature for 10 min. Then the vacuum pump was used (1 min, 90–120 kpa) to help the liquid penetrate into the plant tissue, which was later transferred and placed on 1/2 Murashige and Skoog (MS)‐Gelrite plates containing 1% (w/v) sucrose for 48–72 h.

### Confocal Microscopy

For the subcellular localization assay, the agrobacterium‐infiltrated leaf samples were examined under a LEICA TCS SP8 confocal microscope (Leica Microsystems Inc., Buffalo Grove, NY, USA). The eGFP was excited at 488 nm, and the emission was captured at 510–550 nm. The dsRed was excited at 552 nm, and the emission was captured at 562–632 nm. The chlorophyll autofluorescence was excited at 638 nm, and the emission was captured at 660–730 nm.

For the fluorescence observation of the stems from ToCV‐p22‐dsRed‐infected *N. benthamiana*, the *Z‐*axis scanning method was used for fluorescence imaging. After slicing, the target field of view was moved to the center of the field of view. Then, the *Z*‐axis was adjusted to determine the height of the *Z*‐axis for shooting, and set the *Z‐*axis step to take a photo every 2 µm. After scanning, the scanned images were overlaid to obtain the target image.

### In Vitro Pull‐Down Assay

For the in vitro pull‐down assay, the purified GST‐tag and His‐tag fusion proteins were incubated with Glutathione Sepharose 4B (GE, USA) beads in 500 µL incubation buffer (50 mm Tris‐HCl, pH 8.0, 300 mm NaCl, 0.5% Triton X‐100) for 3 h at 4 °C. The beads were collected and washed three times for 10 min per wash with incubation buffer. The washed beads were boiled in 2 × SDS loading buffer, and proteins were further analyzed through Western blotting with anti‐GST or anti‐His antibodies (CWBIO, Beijing, China).

For a dose‐dependent experiment, equal amounts of His‐NbFBN1.1 and GST‐NbVTE1 were mixed with different amounts of His‐p22 followed by incubation. The mixed protein samples were immunoprecipitated with anti‐GST agarose beads followed by Western blotting assays using an anti‐GST or anti‐His antibody.

### LCI Assay

The *A. tumefaciens* GV3101 strains containing the desired vectors were infiltrated into different regions of *N. benthamiana* leaves. At 3 dpi, the infiltrated leaves were sprayed with 0.2 mm luciferin (Promega, Madison, WI, USA) solution and photographed with a low‐light cooled CCD imaging apparatus (Lumazone PyLoN 2048B, New Jersey, USA) 20 min after harvest.

### Co‐IP Assay

The Co‐IP assay was performed as described.^[^ ^27^ ^]^ The proteins were immunoprecipitated using Flag‐Trap beads (Sigma‒Aldrich) and detected through immunoblotting assays with anti‐Flag and anti‐Myc antibodies (Sigma‒Aldrich).

For a dose‐dependent experiment, NbVTE1‐Myc and NbFBN1.1‐Flag were coexpressed with increasing amounts of p22‐eGFP in *N. benthamiana* leaves. The expressed proteins were immunoprecipitated with anti‐Flag agarose beads. The immunoprecipitated proteins (Flag‐IP), supernatant (Super), and crude extract (Input) were then analyzed through Western blotting assays using an anti‐Flag, anti‐Myc, or anti‐GFP antibody.

### Y2H Assay

For the Y2H assay, the Matchmaker Gold Y2H System was used according to the manufacturer's instructions (Clontech, CA, USA). Yeast was grown on yeast peptone dextrose adenine (YPDA) agar medium for four days, cotransformed with specific combinations of bait and prey constructs, and then grown on SD/‐Leu/‐Trp (SD‐L‐W) or SD/‐Leu/‐Trp/‐His/‐Ade (SD‐L‐W‐H‐Ade) medium for further selections.

### Virus‐Induced Gene Silencing (VIGS)

The TRV‐ or CLCuMuB‐based VIGS assays were performed as previously reported.^[^ ^74^ ^]^

### Chloroplasts, PGs, and Thylakoids Extraction

Intact *N. benthamiana* leaf chloroplasts were prepared as described.^[^ ^75^ ^]^ Briefly, *N. benthamiana* leaf tissues were homogenized in grinding buffer (25 mm HEPES‐KOH, pH 7.7, 330 mm sorbitol, 2 mm EDTA, 1 mm MgCl_2_, 1 mm MnCl_2_) followed by 2 min of centrifugation at 4 °C and 1500 *g*. The resulting crude chloroplast samples were individually transferred onto the PBF Percoll gradient and centrifuged for 10 min at 4 °C and 1500 *g* (horizontal rotation) to collect intact chloroplasts.

For chloroplast quantification, 5 µL of the isolated chloroplast sample was diluted to 1 mL in 80% acetone. After whirlpool shaking, the samples were centrifuged for 2 min at 18 000 *g*. The supernatants were collected and recorded at A_652_. The absorbance of 80% acetone was used as the blank control. The chlorophyll concentration (mg mL^−1^) was calculated using the formula (A_652_/36) × 200.

For PG isolation, equivalent chloroplasts were resuspended in 5 mL R buffer (50 mm HEPES‐KOH, pH 8, 5 mm MgCl_2_) with 0.5 m sucrose. The resuspended samples were ultrasonicated for 10 min at a duty cycle of 15% and then centrifuged for 25 min at 4 °C and 150 000 *g*. The obtained crude PG floating pads were collected and resuspended in 1 mL of R buffer (containing 0.5 m sucrose). The 0.3 mL R buffer (containing 0.2 m sucrose) was first covered and then covered with 0.3 mL R buffer. After 25 min of centrifugation at 4 °C and 380 000 *g*, purified PG were collected for Nile red staining.

For thylakoid extraction, *N. benthamiana* leaf tissues were homogenized in shock buffer (50 mm HEPES‐KOH, pH7.5, 5 mm sorbitol, 5 mm MgCl_2_) followed by 4 min of centrifugation at 4 °C and 5000 *g*. The resulting crude chloroplast samples were resuspended in 1 mL shock buffer and centrifuged for 4 min at 4 °C and 5000 *g* to obtain the thylakoid. Then, the thylakoid was resuspended in 1 mL storage buffer (50 mm HEPES‐KOH, pH7.5, 100 mm sorbitol, 10 mm MgCl_2_) followed by 4 min of centrifugation at 4 °C and 5000 *g*. Then, the thylakoid was resuspended in 0.5 mL storage buffer for further analysis.

### DPA Treatment

DPA (Sigma‒Aldrich) was first dissolved in 100% ethanol to 500 mm and then diluted to 5 µm in sterile distilled water (ddH_2_O) with 0.1% Triton X‐100 immediately before use. The 5 µm DPA solution or ddH_2_O with 0.1% Triton X‐100 (mock) was sprayed onto *N. benthamiana* plant leaves. Twelve hours later, the sprayed plants were inoculated with ToCV. The ToCV‐infected upper leaves were collected at 21 dpi for further analysis.

### Statistical Analysis

The RT‐qPCR data were analyzed using the 2^−ΔΔCT^ method.^[^ ^76^ ^]^ The acquired data were visualized using GraphPad Prism 8.0 software (San Diego, CA, USA), and statistical differences between treatments were determined using a two‐tailed Student's *t*‐test or one‐way ANOVA followed by Tukey's multiple comparisons test. All experiments were performed with at least three biological replicates per treatment with similar results.

## Conflict of Interest

The authors declare no conflict of interest.

## Author Contributions

S.L. and T.Z. designed the experiments. S.L., X.L., Q.Y., and L.Z. performed most experiments. S.L., X.L., Q.Y., X.C., L.Z., J.H., X.Z., Z.F., and T.Z. analyzed data. S.L. and T.Z. wrote the manuscript. All authors agreed with the results and discussions presented in the manuscript.

## Supporting information



Supporting Information

Supporting Information

Supplemental Table 1

## Data Availability

All data needed to evaluate the conclusions in the paper are present in the paper and/or the Supporting Information. Sequences of tomato plastid lipid‐associated protein fibrillin (NM_001247254.1), *NbActin* (JQ256516), ToCV‐BJ RNA1 and RNA2 complete sequences (KC887998, KC887999) were retrieved from the GenBank (https://www.ncbi.nlm.nih.gov/nucleotide). Sequences of *NbFBN1.1* (Niben101Scf09217g01001.1), *NbFBN2.1* (Niben101Scf04980g00021.1), *NbFBN2.2* (Niben101Scf04812g00002.1), *NbFBN2.3* (Niben101Scf06814g01001.1), *NbFBN2.4* (Niben101Scf01305g04002.1), *NbFBN4.1* (Niben101Scf02525g04006.1), *NbFBN4.2* (Niben101Scf00367g02013.1), *NbFBN4.3* (Niben101Scf04177g02015.1), *NbFBN7.1* (Niben101Scf00096g03007.1), *NbFBN7.2* (Niben101Scf02793g27005.1), *NbFBN7.3* (Niben101Scf06661g00007.1), *NbFBN8.1* (Niben101Scf10086g01005.1), *NbFBN8.2* (Niben101Scf04177g02016.1), and *NbVTE1* (Niben101Scf04177g02003.1) were retrieved from the *N. benthamiana* draft genome sequence v1.0.1 (https://solgenomics.net/organism/Nicotiana_benthamiana/genome). Sequence of *FBN1a* (AT4G04020.1), *FBN1b* (AT4G22240.1), *FBN2* (AT2G35490.1), *FBN4* (AT3G23400.1), *FBN7a* (AT3G58010.1), *FBN7b* (AT2G42130.4), *FBN8* (AT2G46910.1) were retrieved from the Arabidopsis information resource (https://www.arabidopsis.org/).
