# Supplementary material for: Chloroplast Fibrillin‐Mediated α‐Tocopherol Biosynthesis Impaired by a Virus to Enhance Infection and to Improve Drought Tolerance
Source: Adv Sci (Weinh). 2025 Oct 29;13(3):e03696. doi: 10.1002/advs.202503696 (PMC12806216; doi:10.1002/advs.202503696)
Supplement: Supplementary file 1 — Supporting Information [file ADVS-13-e03696-s002.pdf]

Supporting Information

Chloroplast fibrillin-mediated α-tocopherol biosynthesis impaired by a virus to enhance infection and to improve drought tolerance

*Sijia Liu[1,2], Xuedong Liu[1,5], Qin Yan[1], Xi Chen[1], Lianyi Zang[1], Jingang Hu[4], Xiaoping Zhu[3], Zaifeng Fan[1], and Tao Zhou[1]**

This PDF file includes:
Figs. S1 to S27

Figure S1

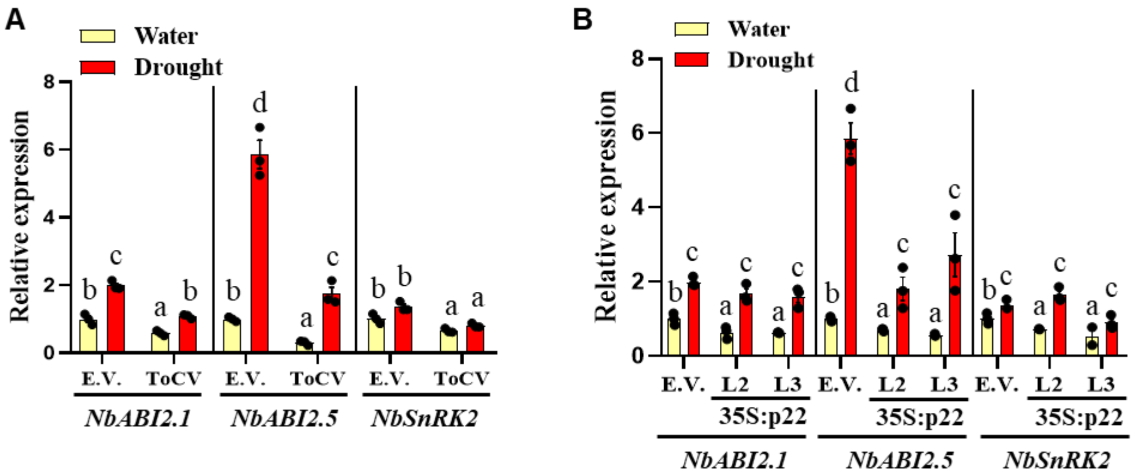

Figure S1. ToCV and its encoded p22 protein confer drought tolerance in *N. benthamiana* through an ABA-independent pathway.

Expression levels of ABA signaling response genes in E.V., ToCV-infected E.V. plants (A) and 35S:p22 transgenic plants (B) under water or drought conditions. Different letters above the bars indicate the statistically significant differences between the treatments, determined by a one-way ANOVA test followed by the Tukey's multiple test ($p < 0.05$). Error bars were SEM. These experiments were performed three times and had six biological replicates per treatment.

Figure S2

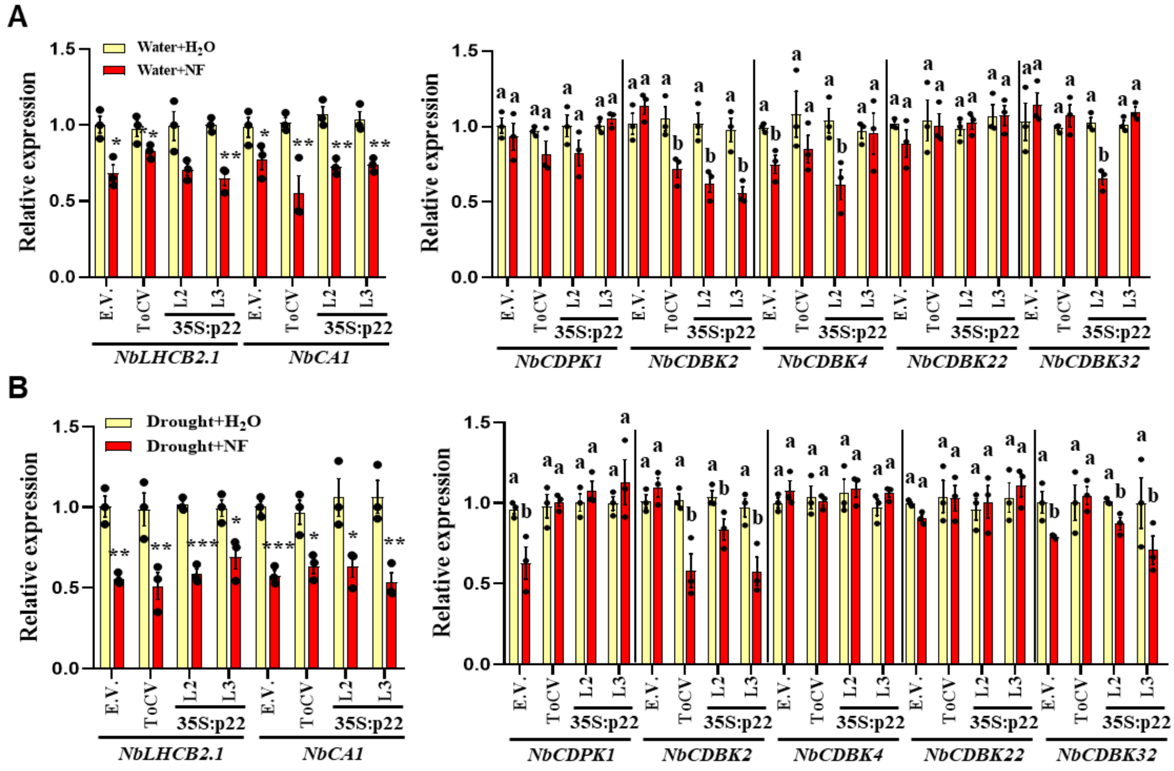

Figure S2. Application of exogenous NF suppresses both the SAL1-PAP retrograde signal and stomatal closure that confers by infection of ToCV or transgenic expression of p22 under normal water and drought conditions.

NF treatment disrupts retrograde signaling under normal water (A) and drought (B) condition, which is represented by the significantly decreased expression levels of the marker genes *NbLHCB2.1* and *NbCA1*. H_2O treatment was as control. The RT-qPCR assay shows the relative expression of *NbCDPKs* in H_2O- or NF-sprayed plants. Asterisks indicate the statistically significant differences between treatments. *, $p < 0.05$, **, $p < 0.01$, ***, $p < 0.001$; determined using the two-tailed Student's *t*-test. Different letters above the bars indicate the statistically significant differences between the treatments, determined by a one-way ANOVA test followed by the Tukey's multiple test ($p < 0.05$). Error bars were SEM. These experiments were performed two times and had eight biological replicates per treatment.

Figure S3

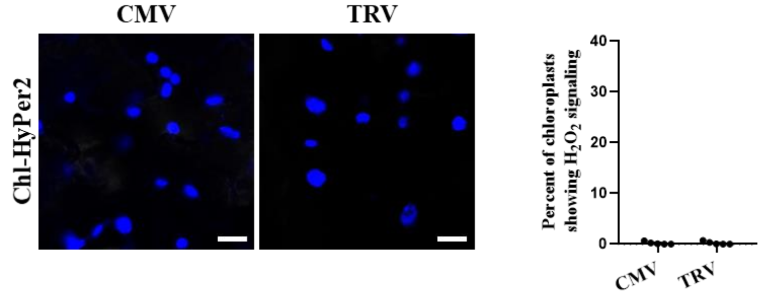

Figure S3. No effect on oxidate homeostasis in chloroplasts of *N. benthamiana* leaves observed following infection by cucumber mosaic virus (CMV) or tobacco rattle virus (TRV).

Chl-HyPer2 response to chloroplast hydrogen peroxide (H_2O_2) elicited by CMV or TRV infection in epidermal cells of *N. benthamiana* upper leaves. Ratiometric images (F488/405 nm) of fluorescence excitation at 488 and 405 nm show the oxidized state of the chloroplast-targeted HyPer2. The blue color indicates the chloroplast. Bars = 20 μm. The right panel was the quantification of the CMV-and TRV-induced changes in Chl-HyPer2 fluorescence. The percentage of H_2O_2-positive chloroplasts (white) among all chloroplasts (blue and white) per visual field is shown.

Figure S4

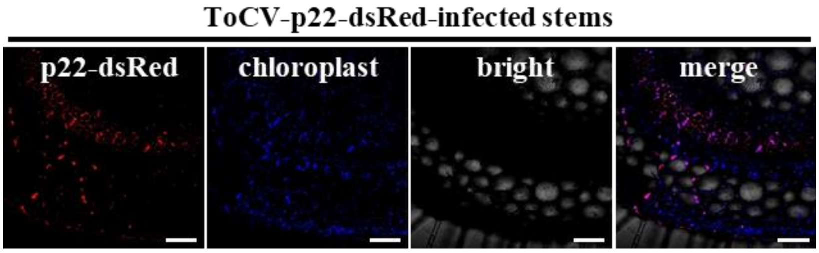

Figure S4. ToCV-encoded p22 co-localizes with chloroplasts during infection of ToCV-p22-dsRed.

The stems samples from ToCV-p22-dsRed infected *N. benthamiana* were collected at 21 dpi. Bars = 200 μm.

Figure S5

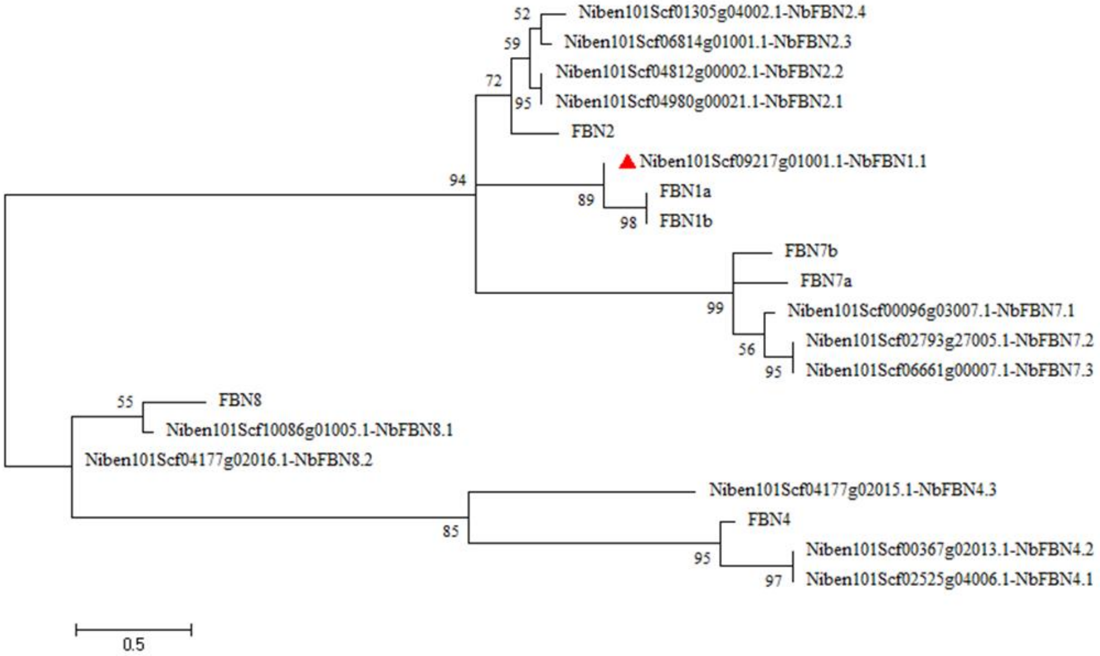

Figure S5. Phylogenetic analysis of FBNs in *Arabidopsis* and *N. benthamiana*.
Maximum likelihood analysis with 1000 replicates was performed using the Mega 6 software. The red triangle marks indicated the gene detected in the following experiments.

Figure S6

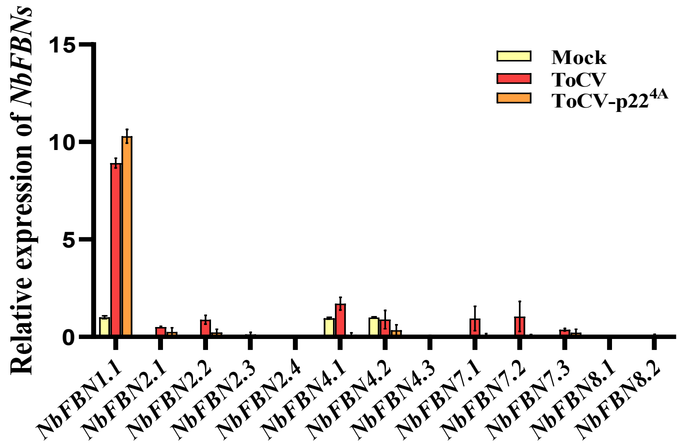

Figure S6. The relative expression of *NbFBNs* under ToCV and ToCV-p22^{4A} infection.

Figure S7

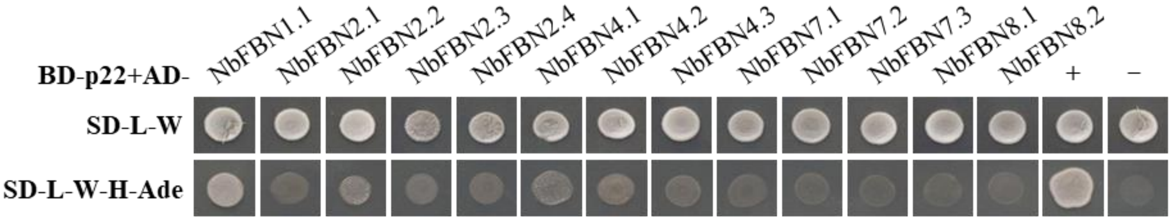

Figure S7. Y2H assay showed the interaction between p22 and NbFBNs.

SD-L-W represents SD-Leu-Trp. SD-L-W-H-Ade represents SD-Leu-Trp-His-Ade.

Figure S8

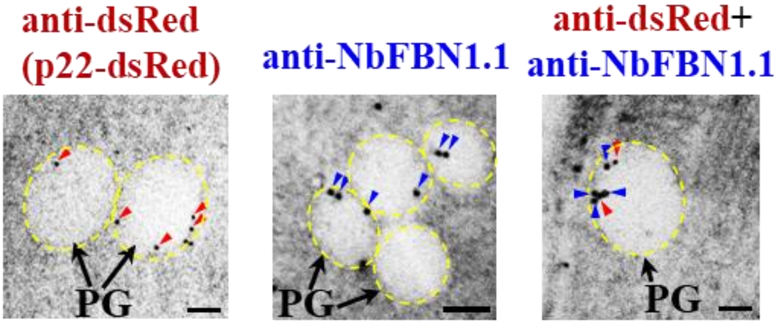

Figure S8. The p22-dsRed co-localizes with NbFBN1.1 in chloroplast PG during infection of ToCV-p22-dsRed.

The left panel: The dsRed-specific IgG as the primary antibody to indicate p22-dsRed in the ToCV-p22-dsRed-infected *N. benthamiana* cells, followed by treatment with 5 nm gold particle-conjugated IgG as the secondary antibody. The middle panel: The NbFBN1.1-specific IgG as the primary antibody to indicate NbFBN1.1 in the ToCV-p22-dsRed-infected *N. benthamiana* cells, followed by treatment with 10 nm gold particle-conjugated IgG as the secondary antibody. The right panel: The double immunogold labeling result confirmed that p22-dsRed (5 nm gold particles) and NbFBN1.1 (10 nm gold particles) were spatially proximal to each other, localizing within the PG of chloroplasts during the infection process. PG were labeled by yellow-dashed line circle, and were indicated by black arrows. Blue and red arrow heads indicate immunogold-labeled NbFBN1.1 and p22-dsRed, respectively. Bars = 85 nm.

Figure S9

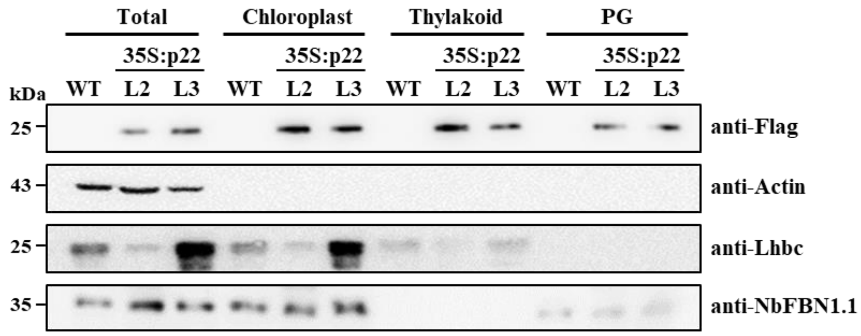

Figure S9. Subcellular fractions confirmed the accumulation of p22.

Chloroplasts, thylakoids, and PGs were isolated from 35S:p22 L2 and L3 plants. The anti-Flag monoclony antibody was used to determine the expression of p22-Flag. Antibodies specific for Actin, Lhbc, and NbFBN1.1 were used as cytoplasmic, thylakoid and PG markers, respectively.

Figure S10

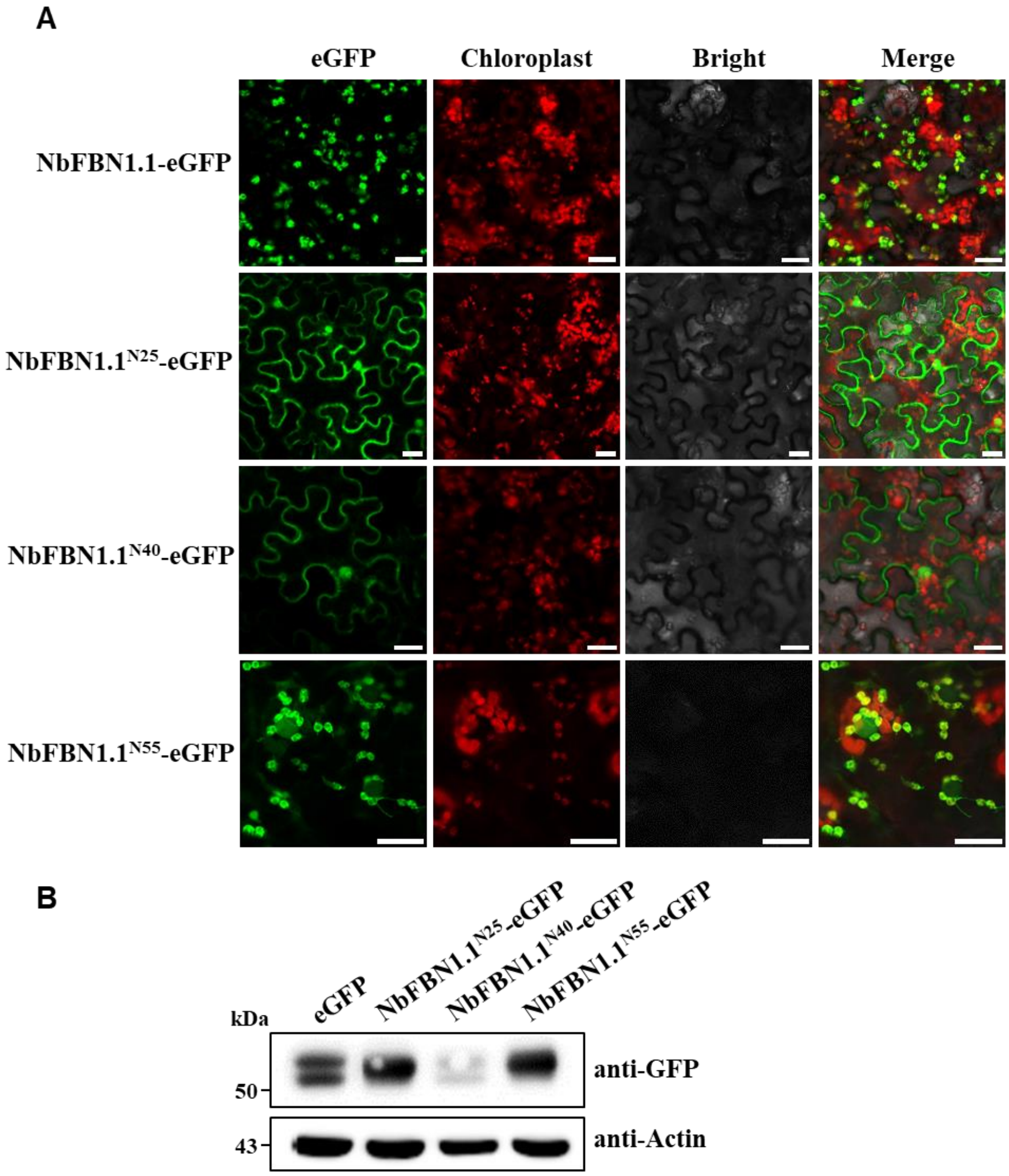

Figure S10. The N-terminal 55 amino acids (aa) of NbFBN1.1 are required for chloroplast targeting.

(A) NbFBN1.1-eGFP, NbFBN1.1^{N25}-eGFP (the N-terminal 25 aa of NbFBN1.1), Nb FBN1.1^{N40}-eGFP (the N-terminal 40 aa), and NbFBN1.1^{N55}-eGFP (the N-terminal 55 aa) are transiently expressed in *N. benthamiana* leaf cells. Confocal images were taken at 3 dpi. Bars = 25 μm. (B) Immunoblotting result shows that these proteins were indeed expressed in the leaves. The expression levels of actin were used to show sample loadings. This experiment was performed three times and had three biological replicates (n=3) per treatment.

Figure S11

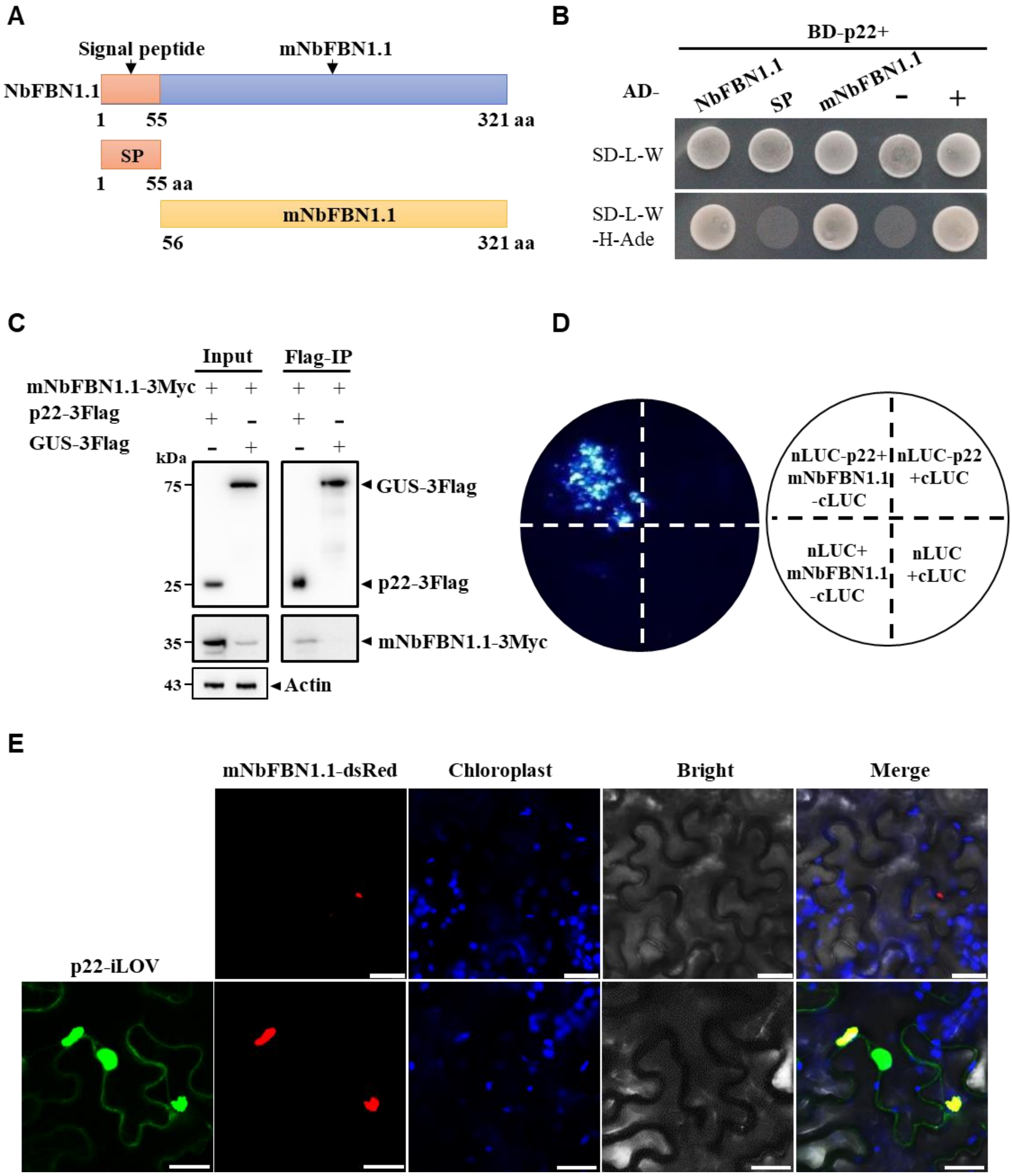

Figure S11. ToCV p22 interacts with the mature domain of NbFBN1.1 (mNbFBN1.1). (A) Schematic representations of full length and two different domains of NbFBN1.1. Signal peptide (SP) is the N-terminal 55 amino acids. The mature domain of NbFBN1.1 (mNbFBN1.1) contains the C-terminal 56-321 aa. Amino acid positions of the domains are indicated. (B) The yeast two-hybrid (Y2H) assay shows that p22 interacts with mNbFBN1.1. (C) The Co-IP assay result shows that p22 interacts with mNbFBN1.1. (D) The LCI assay result also shows that p22 interacts with mNbFBN1.1. (E) Subcellular localization result showed that mNbFBN1.1-dsRed

alone localizes in cytoplasm, and the p22-iLOV and mNbFBN1.1-dsRed co-localized in cytoplasm. Bars = 25 μm.

Figure S12

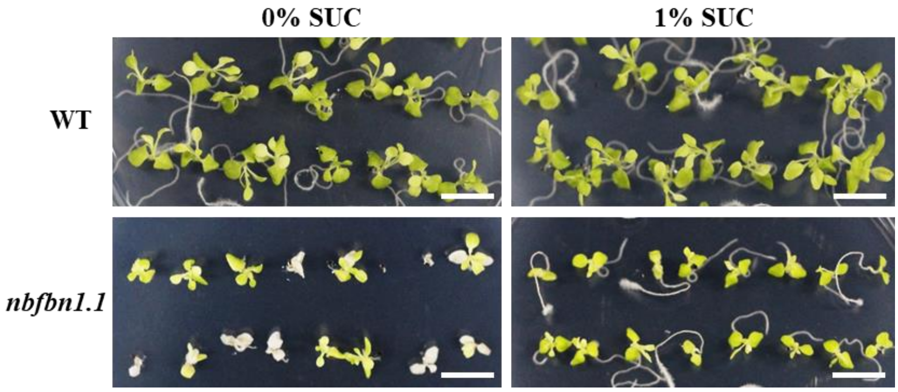

Figure S12. CRISPR/Cas9-generated *NbFBN1.1* knockout (*nbfbn1.1*) *N. benthamiana* lines are seedling-lethal.

Homozygous *nbfbn1.1* mutant seeds were germinated on the agar plates with or without 1% sucrose (SUC). The mutant seedlings stop to grow and become chlorosis on the agar plate without sucrose (left panel). The mutant seedlings grow on the agar plate with 1% sucrose (right panel). Bars = 1 cm.

Figure S13

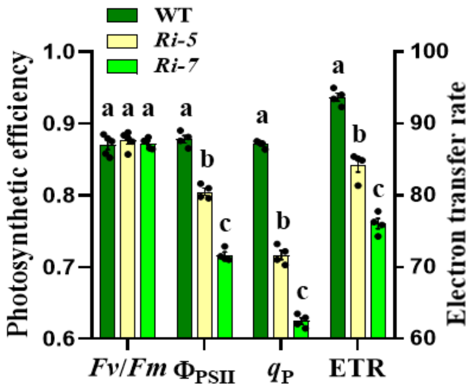

Figure S13. Silencing *NbFBN1.1* reduces photosynthesis.

Analysis of *Fv*/*Fm*, Φ_{PSII}, q_P, and ETR values in the upper leaves from the *NbFBN1.1* RNA interference (RNAi) transgenic *N. benthamiana* lines (e.g., *Ri*-5 and *Ri*-7). Different letters above the bars indicate the statistically significant differences between treatments, determined using one-way ANOVA followed by Tukey's multiple test ($p < 0.05$); error bars are SEM. Each experiment was performed three times, and at least six biological replicates were used per treatment.

Figure S14

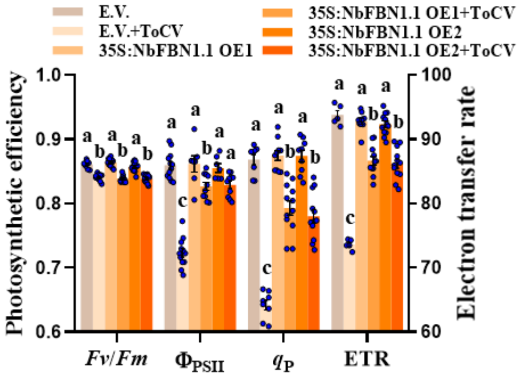

Figure S14. NbFBN1.1 plays an essential role in alleviating damage to photosynthesis. Analysis of *Fv*/*Fm*, Φ_{PSII}, q_P, and ETR values in the upper leaves from the mock- and ToCV-infected E.V. and 35S:NbFBN1.1 plants. Different letters above the bars indicate the statistically significant differences between treatments, determined using one-way ANOVA followed by Tukey's multiple test ($p < 0.05$); error bars are SEM. Each experiment was performed three times, and at least six biological replicates were used per treatment.

Figure S15

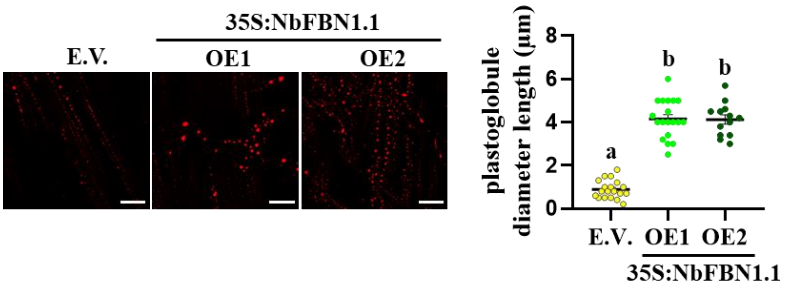

Figure S15. Stable overexpression of NbFBN1.1 in *N. benthamiana* increases the size of PG.

PG were isolated from the leaves of the E.V., and two NbFBN1.1 overexpression-transgenic lines (OE1, and OE2) followed by Nile Red staining (left panel). The size of PG in the leaves from the EV, OE1 and OE2 plants are analyzed and shown in the right panel. Different letters above the bars indicate the statistically significant differences between the treatments, determined using the one-way ANOVA test followed by the Tukey's multiple test ($p < 0.05$), error bars were SEM. Bars = 25 μm. These experiments were performed two times and had three biological replicates (n=3) per treatment.

Figure S16

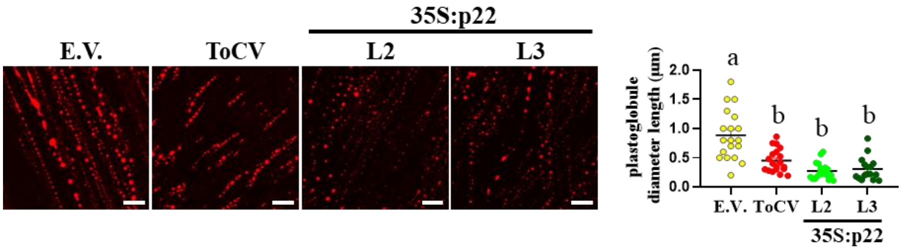

Figure S16. ToCV p22 modulates plastoglobules (PGs) size.

PG were isolated from leaves from mock- and ToCV-inoculated E.V. or two p22-transgenic lines followed by Nile Red staining. The right panel shows the statistical analysis of PG diameters. Different letters above the bars indicate statistically significant differences between the treatments, determined using one-way ANOVA followed by Tukey's multiple test ($p < 0.05$). Error bars were SEM. Bars = 15 µm. These experiments were performed three times and had three biological replicates (n=3) per treatment.

Figure S17

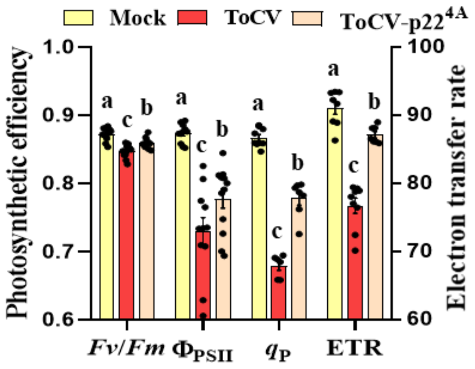

Figure S17. The p22-NbFBN1.1 interaction is essential for suppressing photosynthesis. The values of *Fv*/*Fm*, Φ_{PSII}, q_P, and ETR in the mock, ToCV-, and ToCV-$p22^{4A}$-infected plant leaves. Different letters above the bars indicate statistically significant differences between the treatments, determined using one-way ANOVA followed by Tukey's multiple test ($p < 0.05$). Error bars were SEM. These experiments were performed three times and had three biological replicates (n = 3) per treatment.

Figure S18

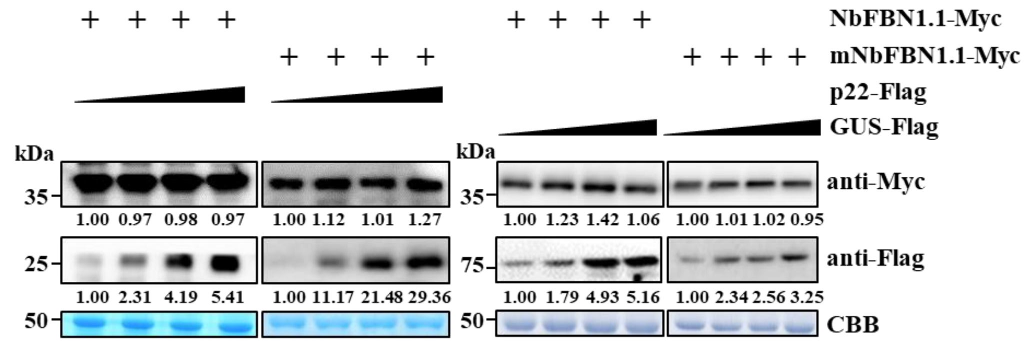

Figure S18. ToCV p22 does not influence the accumulation levels of NbFBN1.1.

The dosage-dependent effect of p22 on the protein accumulation levels of NbFBN1.1 and mNbFBN1.1 in the co-expression assay. The NbFBN1.1-Myc or mNbFBN1.1-Myc was co-expressed with increasing amounts of p22-Flag in *N. benthamiana* leaves. The GUS-Flag was used as the control. For immunoblotting assays, anti-Flag and anti-Myc antibodys were used. The CBB-stained gel is used to show sample loadings.

Figure S19

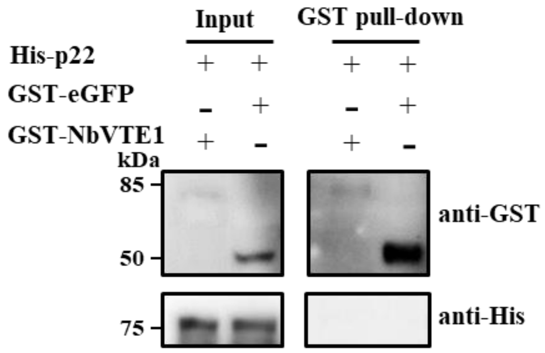

Figure S19. The GST-pull down assay determined the interaction between p22 and NbVTE1 *in vivo*.

Figure S20

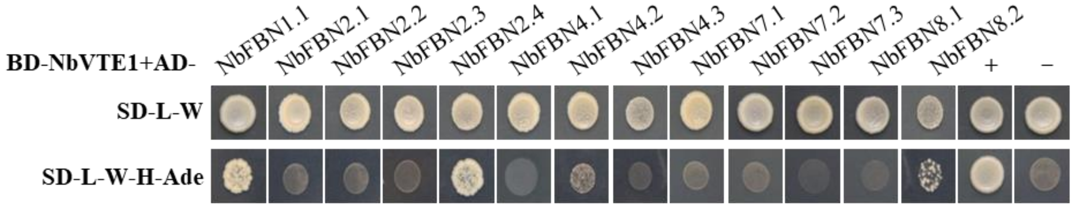

Figure S20. Y2H assay showed the interaction between NbVTE1 and NbFBNs.

SD-L-W represents SD-Leu-Trp. SD-L-W-H-Ade represents SD-Leu-Trp-His-Ade.

Figure S21

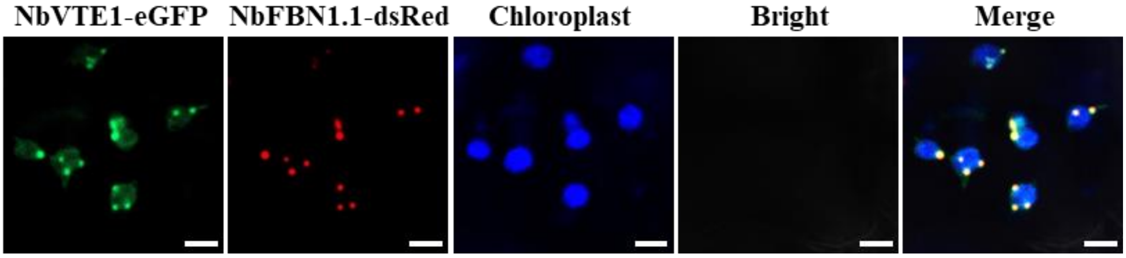

Figure S21. NbVTE1 co-localizes with NbFBN1.1 in PG.

Confocal images were captured at 3 dpi. Bars = 10 μm. The merged image indicates that NbVTE1-eGFP and NbFBN1.1-dsRed co-localizes in the PG of the chloroplast. This experiment was performed three times and had three biological replicates.

Figure S22

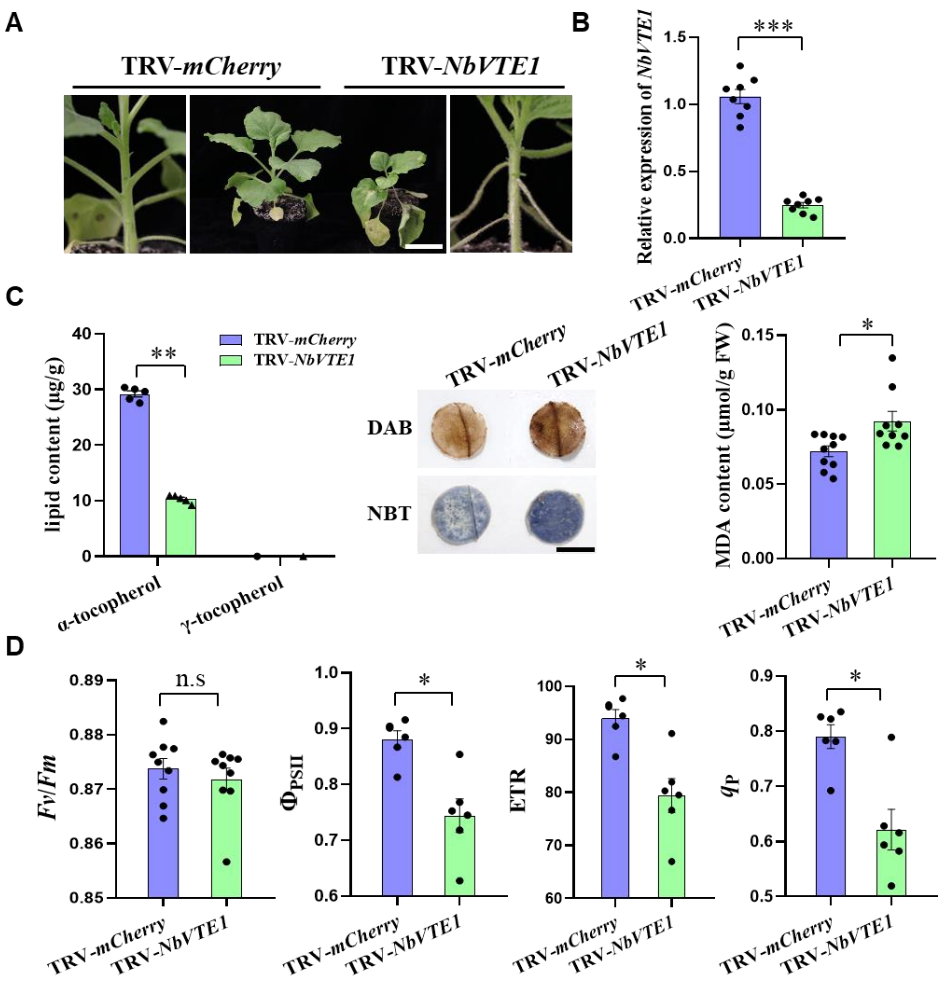

Figure S22. Silencing of *NbVTE1* expression in plant suppresses α-tocopherol biosynthesis while increases lipid oxidation.

(A) The expression of *NbVTE1* in *N. benthamiana* plants was silenced using a TRV-based vector. The *NbVTE1*-silenced plants are strongly stunted compared to the non-silenced control plants. Bar = 5 cm. (B) The result of RT-qPCR assay confirms that the expression level of *NbVTE1* in the TRV-*NbVTE1* plants decreases 70% compared to the TRV-*mCherry* control plants. (C) HPLC analysis of the accumulation level of α-tocopherol and γ-tocopherol in upper leaves from *NbVTE1*-silenced plants (TRV-*NbVTE1*) and non-silenced control (TRV-*mCherry*) plants. Histochemical staining of leaf discs from the *NbVTE1*-silenced and the non-silenced plants using DAB for H_2O_2 production and NBT for O_2^- production. Bar = 1 cm. Analysis of MDA content indicates that the lipid peroxidation in *NbVTE1*-silenced plants increases significantly compared to non-silenced control plants. (D) The results of chlorophyll fluorescence analyses show that, except *Fv*/*Fm*, the values of Φ_{PSII}, ETR, and q_P decrease in the *NbVTE1*-silenced plants. Asterisks in (B, C, D) indicate the statistically significant difference between treatments. n.s, not significant; *, $p < 0.05$; **, $p < 0.01$; ***, $p < 0.001$; determined

using the two-tailed Student's *t*-test. Error bars were SEM. These experiments were performed three times and had at least six biological replicates per treatment.

Figure S23

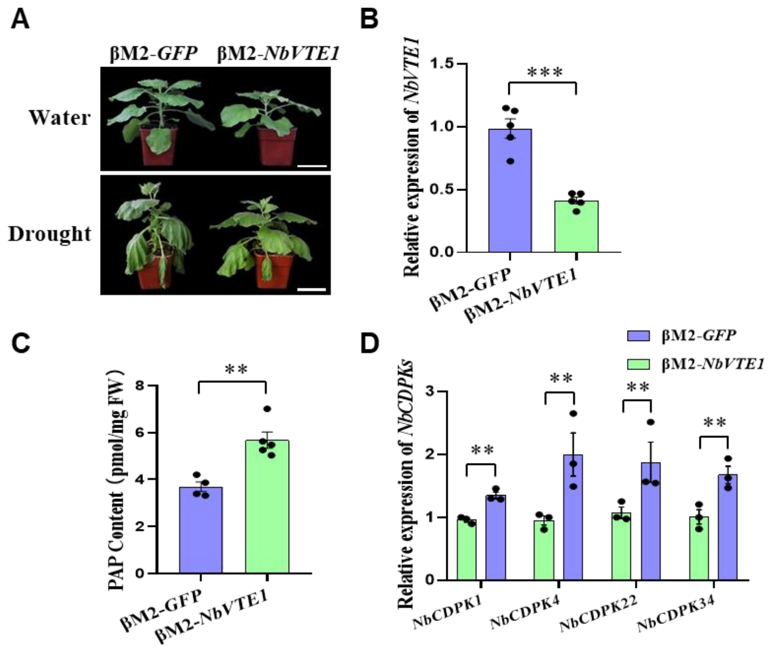

Figure S23. Silencing *NbVTE1* stimulates SAL1-PAP retrograde signal and improves plant drought tolerance.

(A) Phenotypes of *NbVTE1*-silenced plants (βM2-*NbVTE1*) and control plants (βM2-*GFP*) under water and drought conditions. Bars = 5 cm. (B) RT-qPCR confirmed that the expression level of *NbVTE1* was downregulated in βM2-*NbVTE1*-inoculated plants compared to the control (βM2-*GFP*). (C) PAP levels in *NbVTE1*-silenced and control plant leaves. (D) The transcriptional levels of *NbCDPKs* in *NbVTE1*-silencing plants.

Figure S24

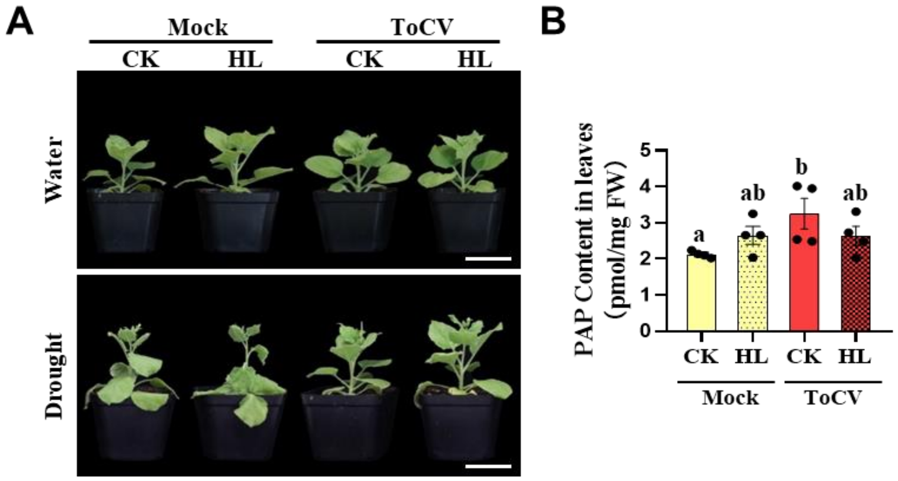

Figure S24. The drought tolerance of mock- and ToCV-infected wild type *N. benthamiana* plants under high-light stress.

(A) Phenotypes of high-light-treated mock- or ToCV-infected wild type *N. benthamiana* plants under water and drought conditions. Bars = 5 cm. (B) PAP levels in the high-light-treated mock- or ToCV-infected plant leaves.

Figure S25

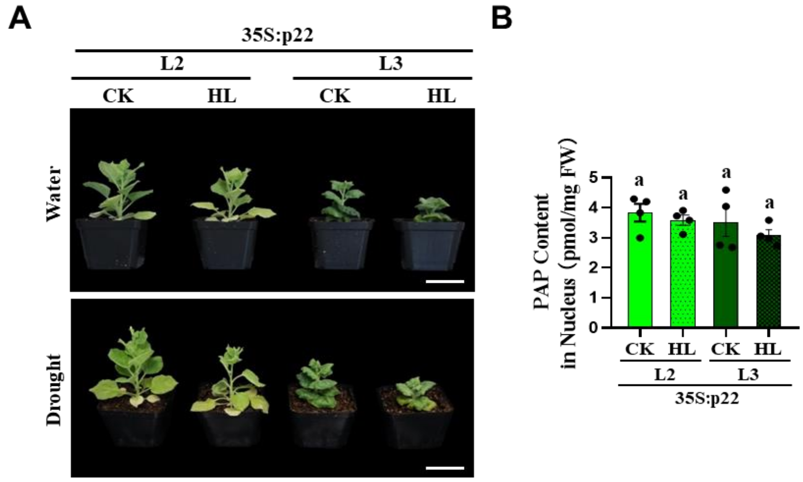

Figure S25. The drought tolerance of 35S:p22 *N. benthamiana* plants under high-light stress.

(A) Phenotypes of high-light-treated 35S:p22 *N. benthamiana* plants under water and drought conditions. Bars = 5 cm. (B) PAP levels in the high-light-treated 35S:p22 plant leaves.

Figure S26

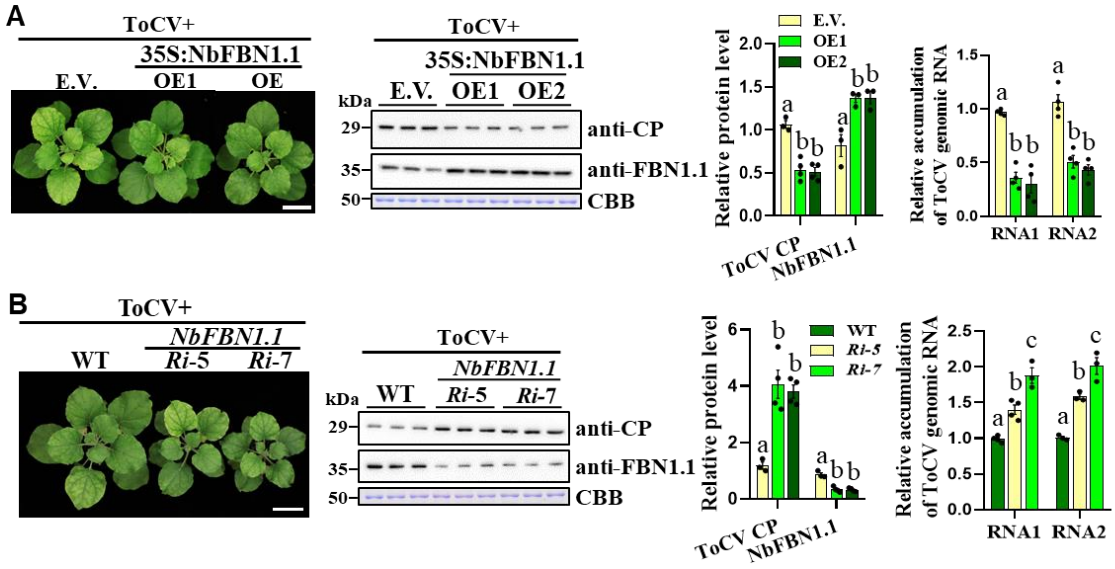

Figure S26. NbFBN1.1 plays an essential role in suppressing ToCV infection.

(A) Overexpression of *NbFBN1.1* in *N. benthamiana* plants (OE1 and OE2) suppressed ToCV infection, resulting in attenuated chlorosis symptoms and lower levels of ToCV coat protein (CP) and genomic RNA. The levels of ToCV CP and NbFBN1.1 in E.V. were taken to be 1.00. RT-qPCR analysis of the levels of ToCV RNA1 and RNA2 in NbFBN1.1 OE1 and OE2 transgenic and E.V.-transformed plants. (B) ToCV caused more severe symptoms and higher levels of CP and genomic RNA in *NbFBN1.1* RNAi (*Ri*-5 and *Ri*-7) plants than in WT plants. The levels of ToCV CP and NbFBN1.1 in WT were taken to be 1.00. The expression level of *NbActin* was used as an internal control for RT-qPCR analysis. The CBB-stained rubisco large subunit served as the loading control for Western blotting analysis. The relative densities of each band detected in Western blotting were analyzed using ImageJ software. Different letters above the bars indicate statistically significant differences between the treatments, determined using one-way ANOVA followed by Tukey's multiple test ($p < 0.05$). Error bars were SEM. These experiments were performed three times and had at least six biological replicates per treatment.

Figure S27

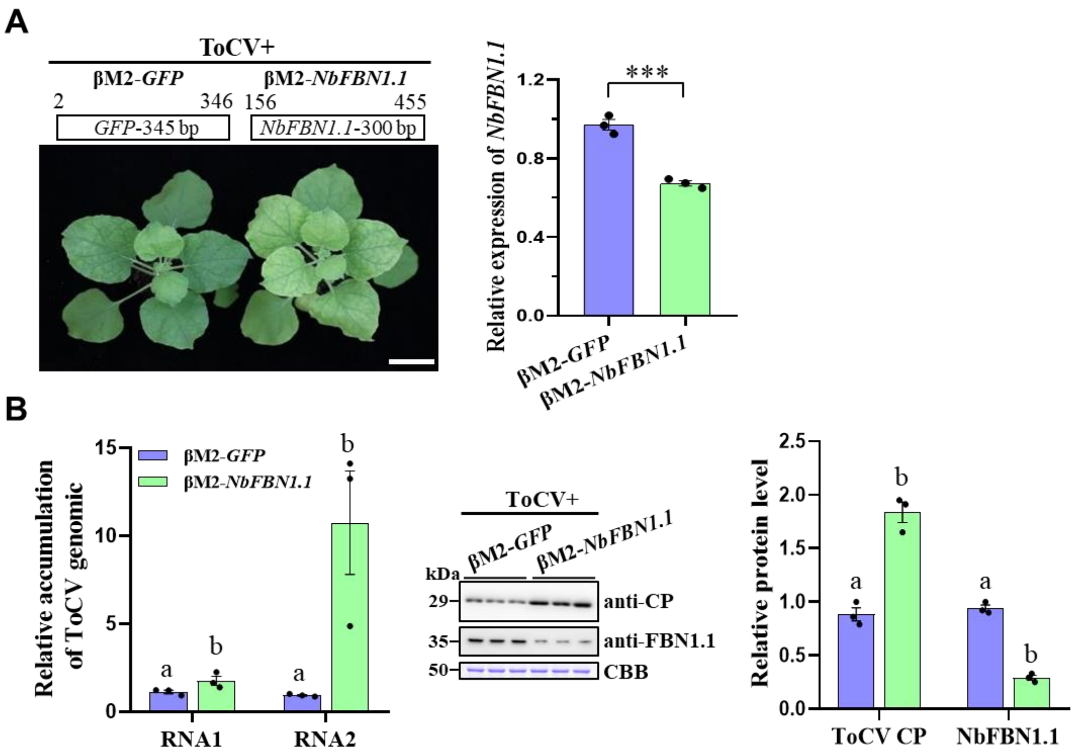

Figure S27. Silencing of *NbFBN1.1* expression in plant benefits ToCV infection.

(A) ToCV infection causes more severe chlorosis in the leaves of the βM2-*NbFBN1.1* plants compared to the βM2-*GFP* plants. The assayed plants were photographed at 21 dpi of ToCV. Bar = 5 cm. The result of RT-qPCR assay confirms that the expression of *NbFBN1.1* is significantly silenced in the βM2-*NbFBN1.1*-inoculated plants. Asterisks indicate the statistically significant differences between the treatments (*, $p < 0.05$; ***, $p < 0.001$), determined using the two-tailed Student's *t*-test, error bars were SEM. (B) The accumulation levels of ToCV genomic RNA1 and RNA2 increase significantly in the *NbFBN1.1*-silenced plants. The expression level of *NbActin* in these plants was used as an internal control. Immunoblotting assay result also shows that the accumulation level of ToCV CP increases significantly in the *NbFBN1.1*-silenced plants, while the expression level of NbFBN1.1 decrease in these *NbFBN1.1*-silenced plants. The CBB-stained rubisco large subunit gel was used to show sample loadings. The relative densities of each band detected in immunoblotting were analyzed using the ImageJ software and the level of ToCV CP and NbFBN1.1 in βM2-*GFP* plants were taken to be 1.00, respectively. Different letters above the bars indicate statistically significant differences between the treatments, determined using one-way ANOVA followed by Tukey's multiple test ($p < 0.05$). Error bars were SEM. These experiments were performed three times and had at least six biological replicates per treatment.
