# Supplementary material for: Chloroplast Fibrillin‐Mediated α‐Tocopherol Biosynthesis Impaired by a Virus to Enhance Infection and to Improve Drought Tolerance
Source: Adv Sci (Weinh). 2025 Oct 29;13(3):e03696. doi: 10.1002/advs.202503696 (PMC12806216; doi:10.1002/advs.202503696)
Supplement: Supplementary file 2 — Supporting Information [file ADVS-13-e03696-s001.pdf]

Supplemental Figure

Chloroplast fibrillin-mediated α-tocopherol biosynthesis impaired by a virus to enhance infection and to improve drought tolerance

*Sijia Liu[1,2], Xuedong Liu[1,5], Qin Yan[1], Xi Chen[1], Lianyi Zang[1], Jingang Hu[4], Xiaoping Zhu[3], Zaifeng Fan[1], and Tao Zhou[1]**

Figure 1

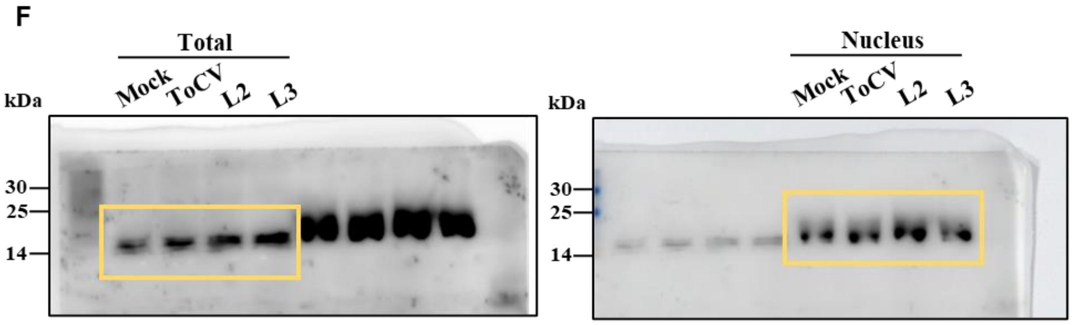

Primary antibody: Histone H3.3 Rabbit pAb (ABclonal, A13824), 1:5000
Secondary antibody: Goat anti-Rabbit (CWBio, CW0103S), 1:5000

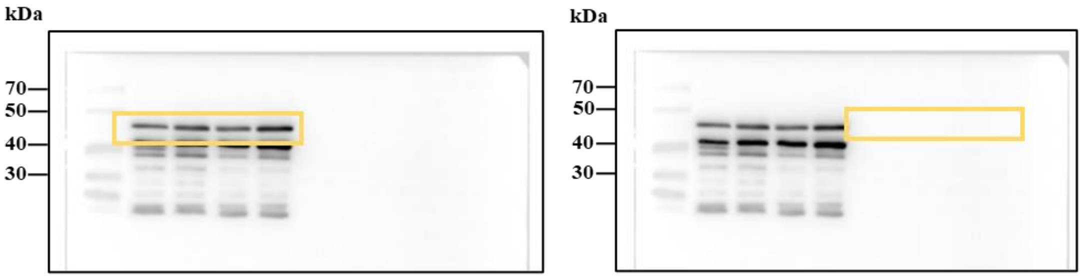

Primary antibody: Anti β-Actin Mouse Monoclonal Antibody (CWBio, CW0096M), 1:5000
Secondary antibody: Goat anti Mouse (CWBio, CW0102S), 1:5000

Figure 2

B

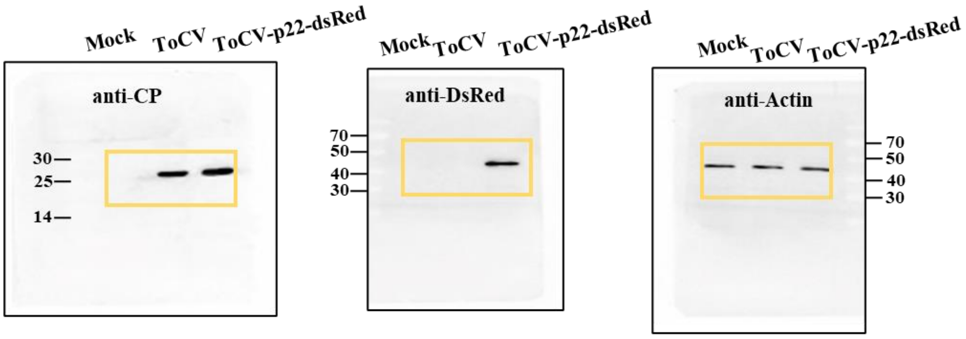

Primary antibody: Anti-CP, Rabbit Polypeptides Epitope (NGGDRNPLVRPLDDC), 1:1000
Anti-DsRed2 mouse monoclonal antibody (Sangon Biotech), 1:5000
Anti β-Actin Mouse Monoclonal Antibody (CWBio, CW0096M), 1:5000
Secondary antibody: Goat anti-Mouse (CWBio, CW0102S), 1:5000
Goat anti-Rabbit (CWBio, CW0103S), 1:5000

E

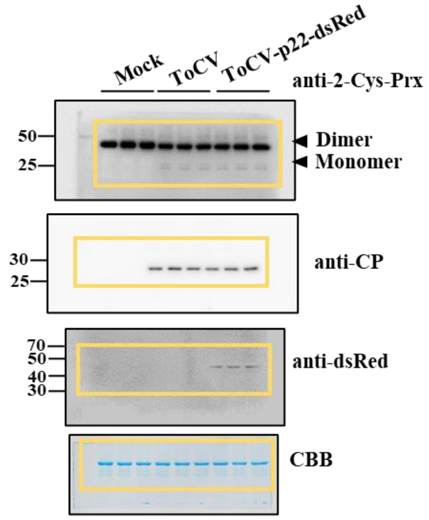

J

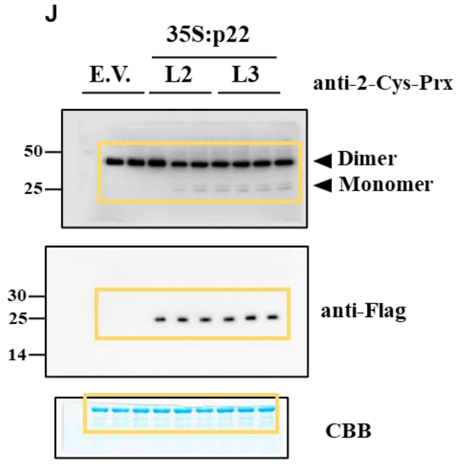

Primary antibody: Anti-2-Cys-Prx, Obtained from Prof. Dawei Li, China Agricultural University, 1:2000
Anti-CP, Rabbit Polypeptides Epitope (NGGDRNPLVRPLDDC), 1:1000
Anti-DsRed2 mouse monoclonal antibody (Sangon Biotech), 1:5000
Anti-Flag M2-Peroxidase (HRP) antibody (Sigma, A8592), 1:10000
Secondary antibody: Goat anti-Mouse (CWBio, CW0102S), 1:5000
Goat anti-Rabbit (CWBio, CW0103S), 1:5000

Figure 3

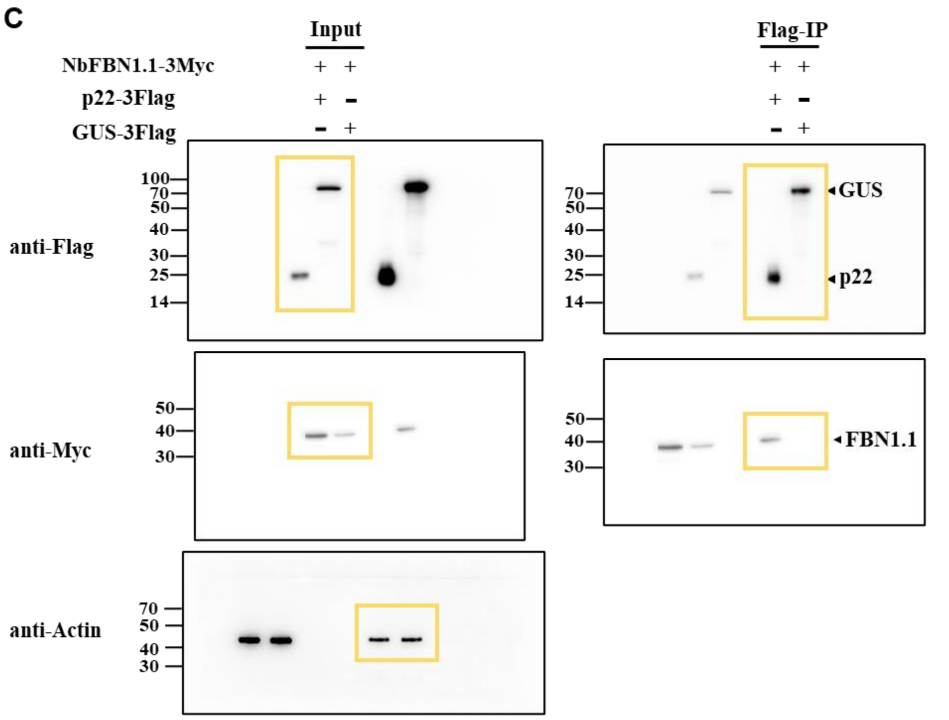

Primary antibody: Anti-Flag M2-Peroxidase (HRP) antibody (Sigma, A8592), 1:10000
Anti-c-Myc Peroxidase (HRP) antibody (Sigma, A5598), 1:5000
Anti β-Actin Mouse Monoclonal Antibody (CWBio, CW0096M), 1:5000
Secondary antibody: Goat anti-Mouse (CWBio, CW0102S), 1:5000

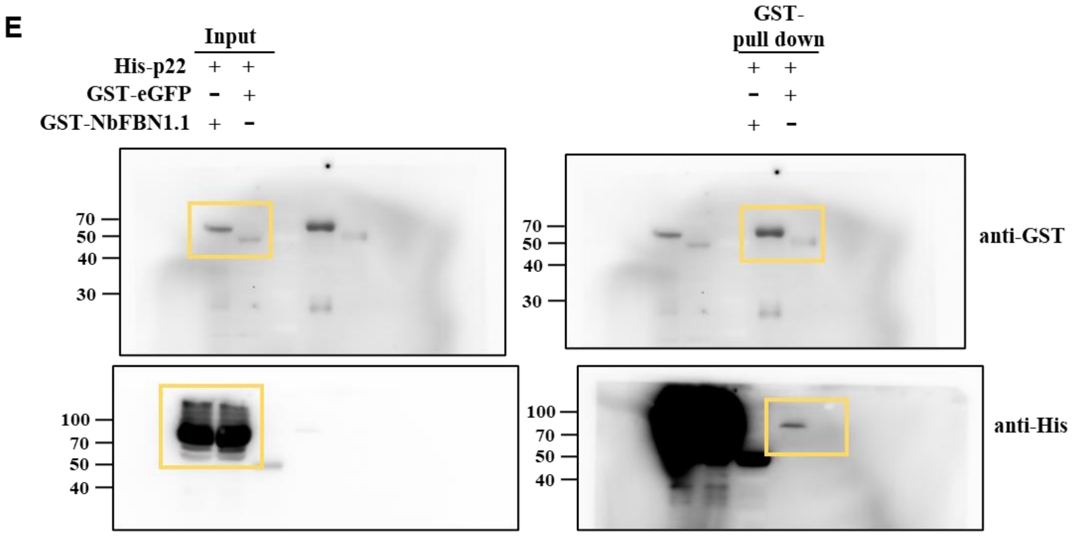

Primary antibody: Anti-GST-Tag Mouse Monoclonal Antibody (CWBio, CW0084M), 1:5000
Anti- His-Tag Mouse Monoclonal Antibody (CWBio, CW0286M), 1:5000
Secondary antibody: Goat anti-Mouse (CWBio, CW0102S), 1:5000

Figure 3

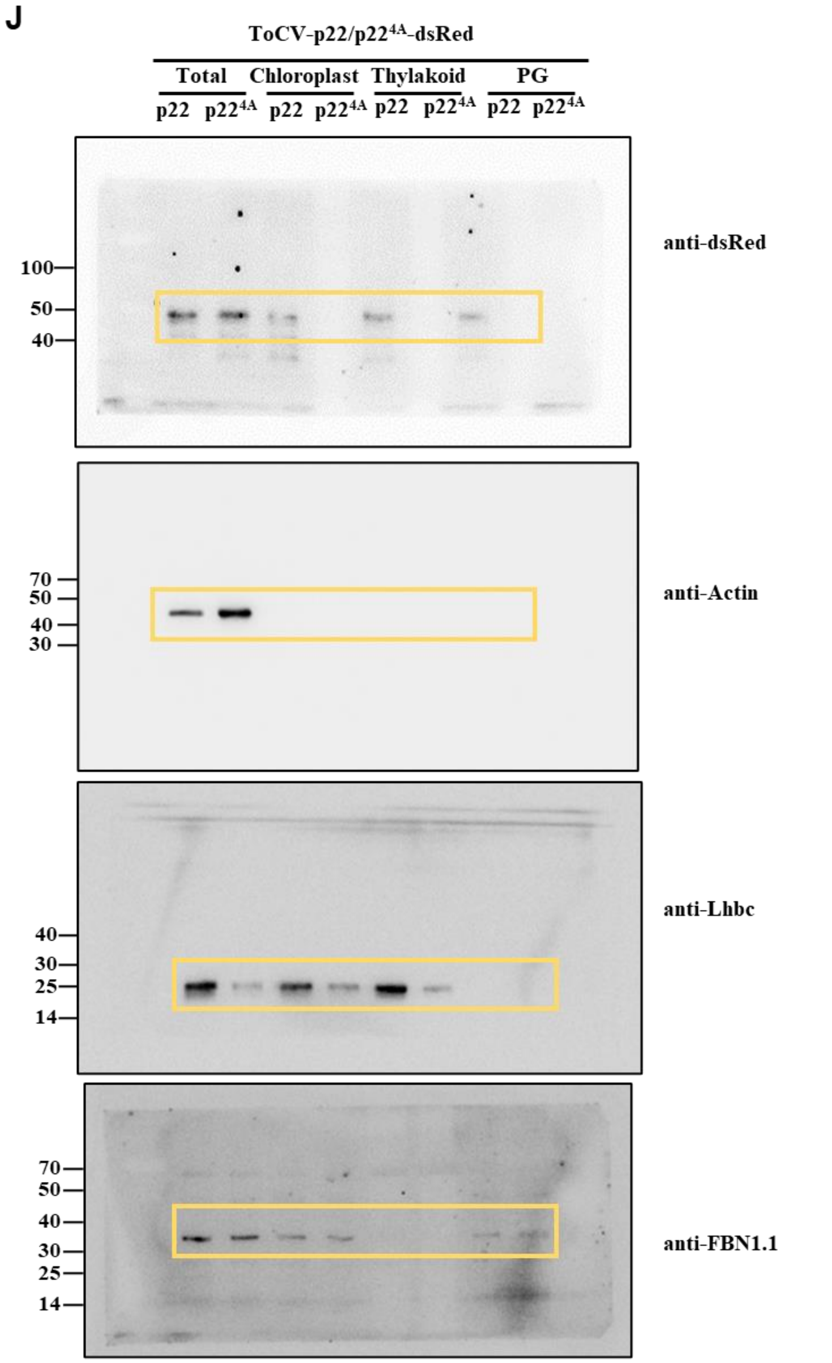

Primary antibody: Anti-DsRed2 mouse monoclonal antibody (Sangon Biotech), 1:5000
Anti β-Actin Mouse Monoclonal Antibody (CWBio, CW0096M), 1:5000
Anti-Lhcb1 Rabbit Polyclonal Antibody (Agrisera, AS09-522), 1:5000
Anti-FBN1.1, Rabbit Polypeptides Epitope (LITKLESMNPTPAPC), 1:1000
Secondary antibody: Goat anti-Mouse (CWBio, CW0102S), 1:5000
Goat anti-Rabbit (CWBio, CW0103S), 1:5000

Figure 4

B

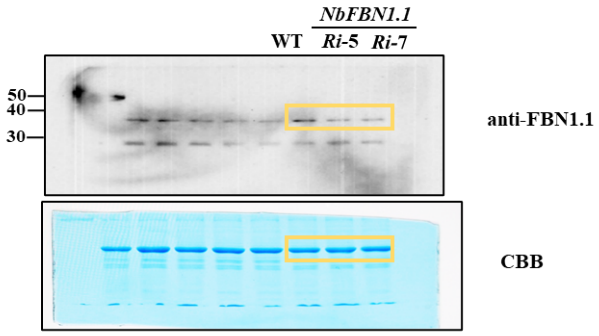

Primary antibody: Anti-FBN1.1, Rabbit Polypeptides Epitope (LITKLESMNPTPAPC), 1:1000
Secondary antibody: Goat anti-Rabbit (CWBio, CW0103S), 1:5000

E

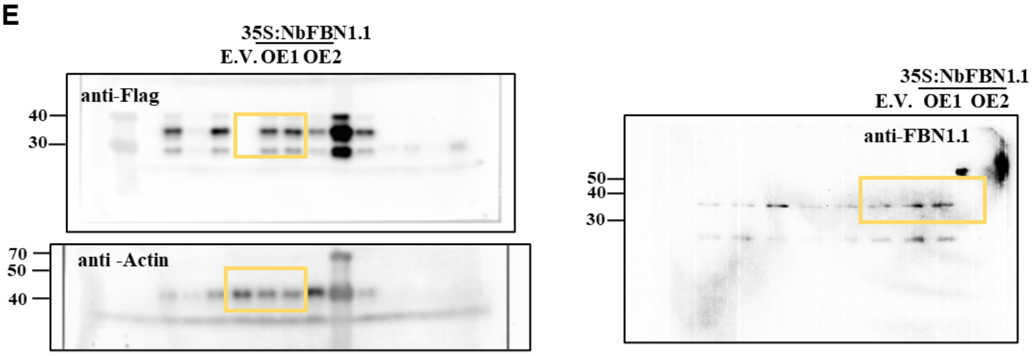

Primary antibody: Anti-Flag M2-Peroxidase (HRP) antibody (Sigma, A8592), 1:10000
Anti β-Actin Mouse Monoclonal Antibody (CWBio, CW0096M), 1:5000
Anti-FBN1.1, Rabbit Polypeptides Epitope (LITKLESMNPTPAPC), 1:1000
Secondary antibody: Goat anti-Mouse (CWBio, CW0102S), 1:5000
Goat anti-Rabbit (CWBio, CW0103S), 1:5000

J

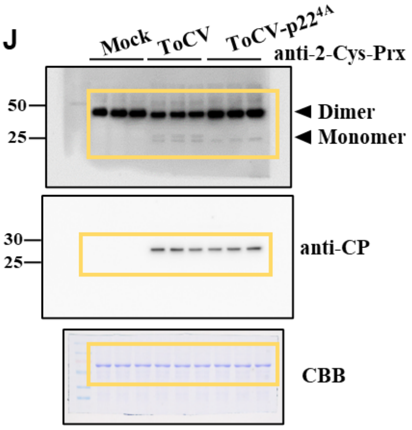

Primary antibody: Anti-2-Cys-Prx, Obtained from Prof. Dawei Li, China Agricultural University, 1:2000
Anti-CP, Rabbit Polypeptides Epitope (NGGDRNPLVRPLDDC), 1:1000
Secondary antibody: Goat anti-Rabbit (CWBio, CW0103S), 1:5000

Figure 5

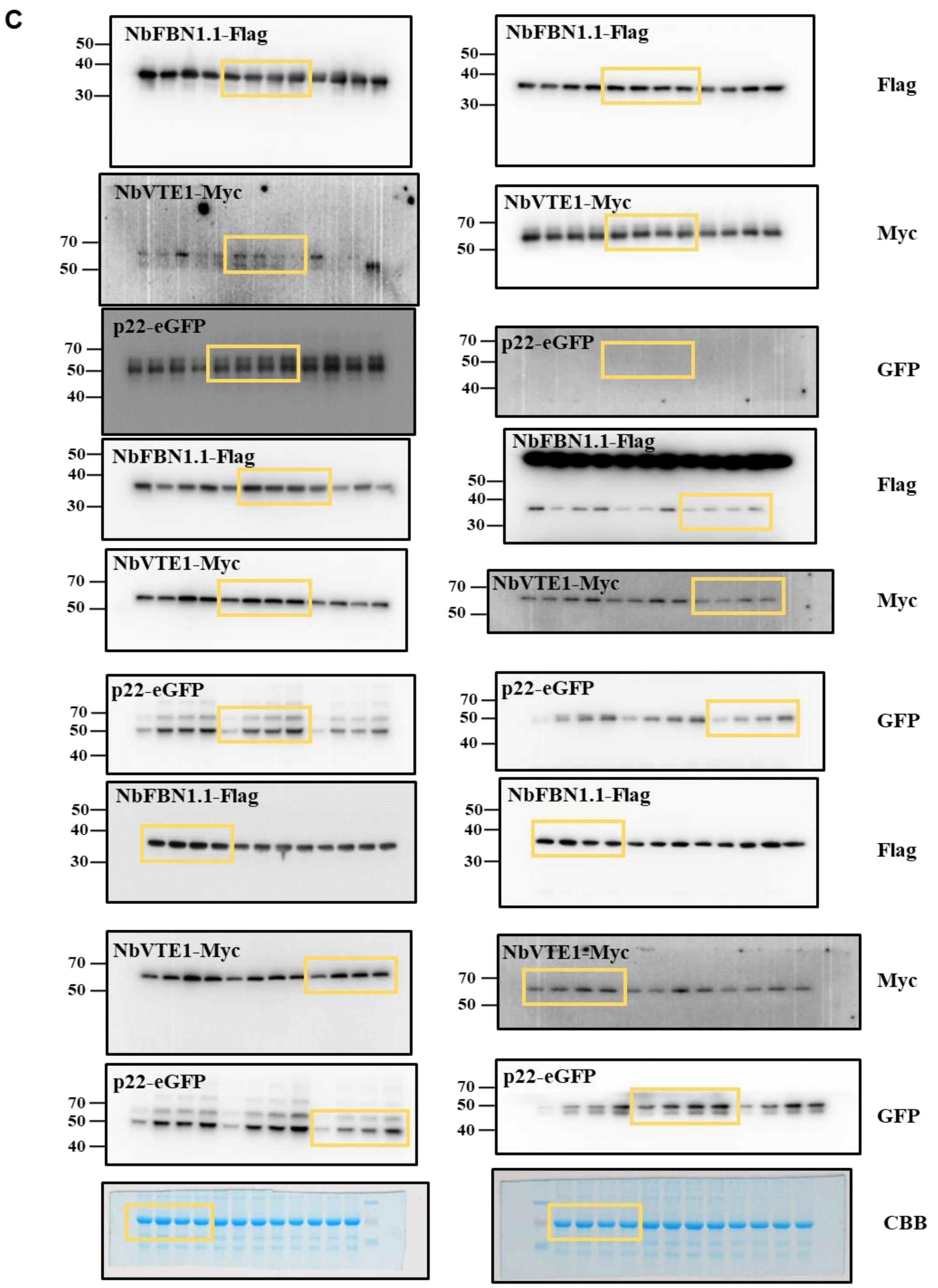

Primary antibody: Anti-Flag M2-Peroxidase (HRP) antibody (Sigma, A8592), 1:10000
Anti-c-Myc Peroxidase (HRP) antibody (Sigma, A5598), 1:5000
Anti-GFP Mouse Monoclonal Antibody (TransGene Biotech, HT801-01), 1:10000
Secondary antibody: Goat anti-Mouse (CWBio, CW0102S), 1:5000

Figure 5

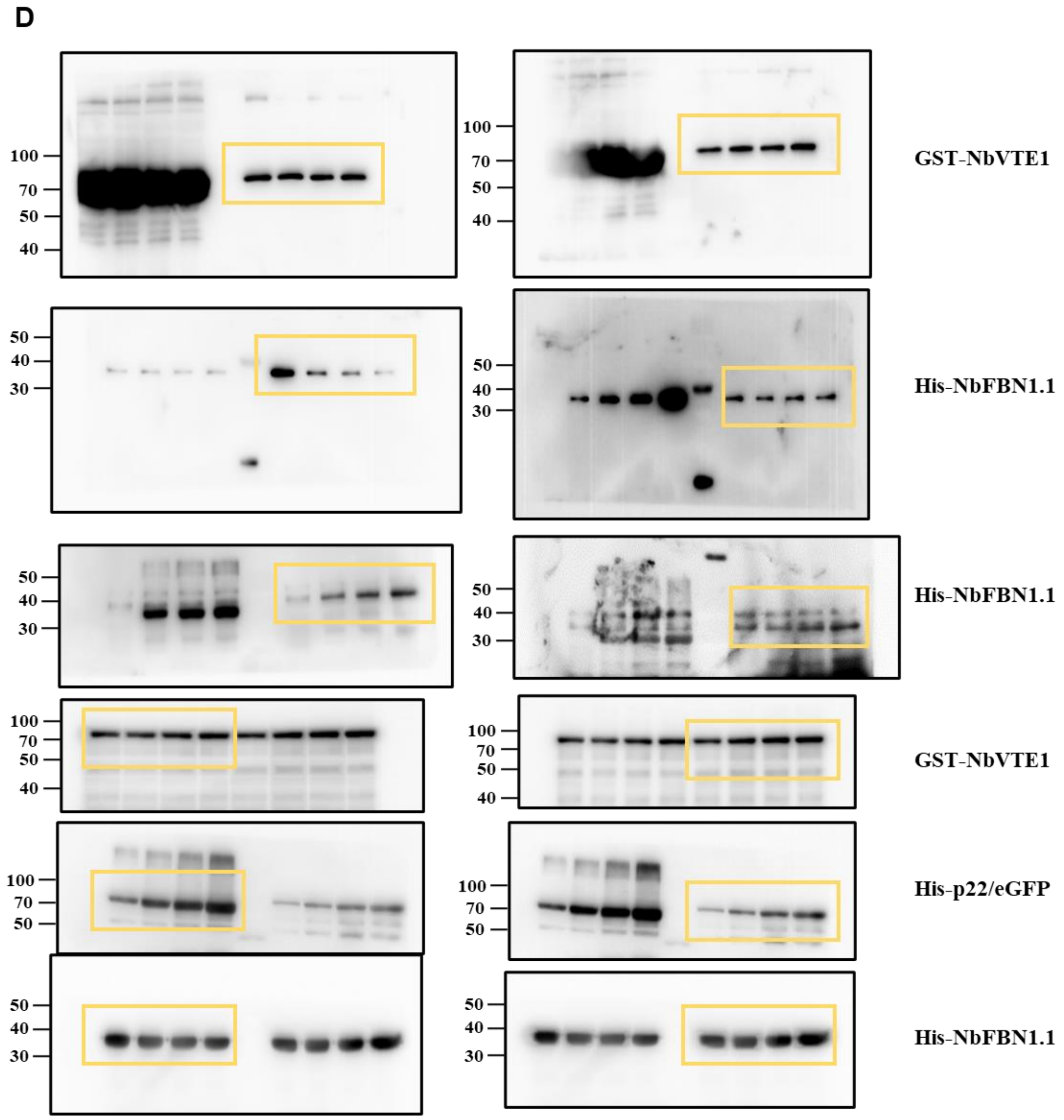

Primary antibody: Anti-GST-Tag Mouse Monoclonal Antibody (CWBio, CW0084M), 1:5000
Anti- His-Tag Mouse Monoclonal Antibody (CWBio, CW0286M), 1:5000

Secondary antibody: Goat anti-Mouse (CWBio, CW0102S), 1:5000

Figure 5

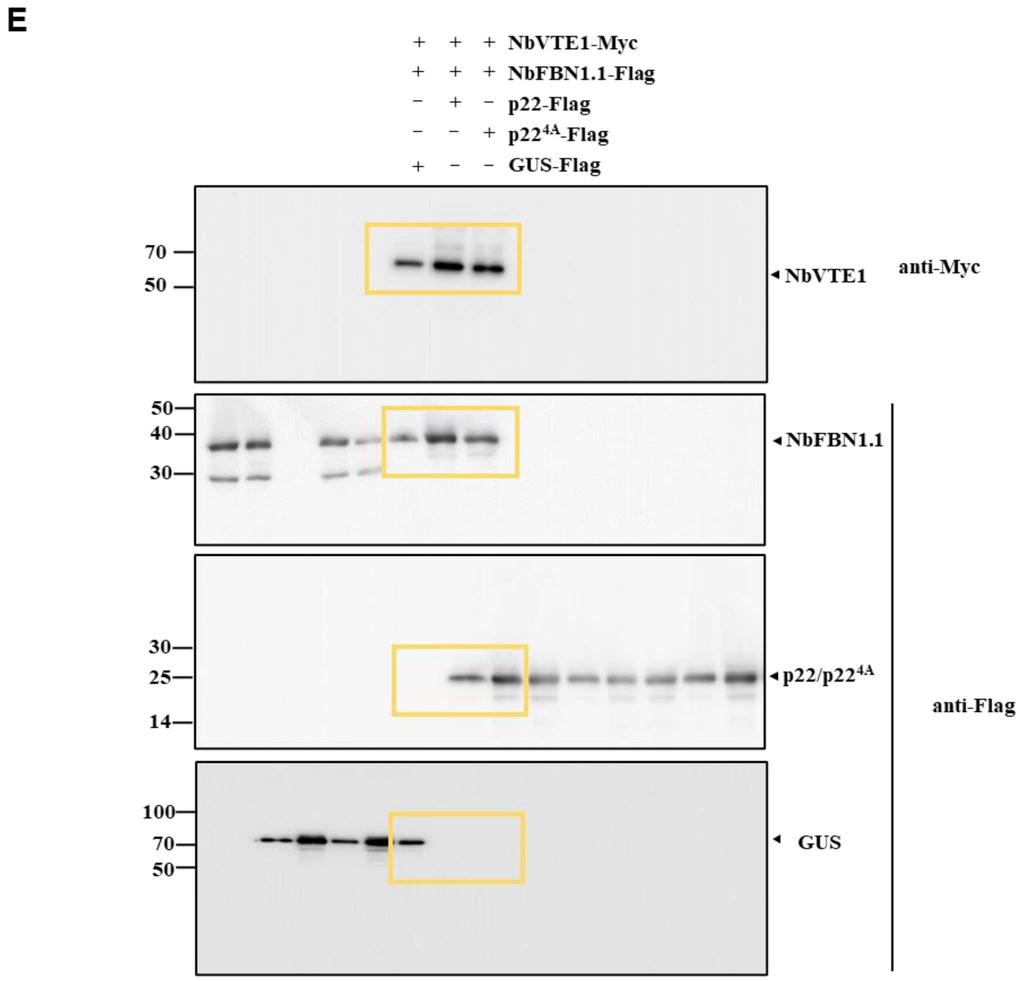

Primary antibody: Anti-Flag M2-Peroxidase (HRP) antibody (Sigma, A8592), 1:10000
Anti-c-Myc Peroxidase (HRP) antibody (Sigma, A5598), 1:5000

Figure 6

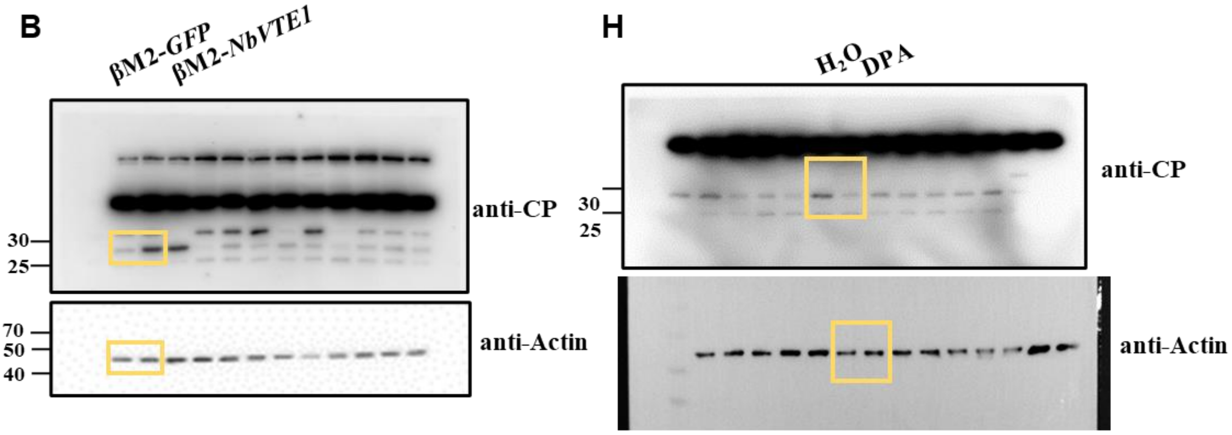

Primary antibody: Anti-CP, Rabbit Polypeptides Epitope (NGGDRNPLVRPLDDC), 1:1000
Anti β-Actin Mouse Monoclonal Antibody (CWBio, CW0096M), 1:5000
Secondary antibody: Goat anti-Rabbit (CWBio, CW0103S), 1:5000
Goat anti-Mouse (CWBio, CW0102S), 1:5000

Figure 7

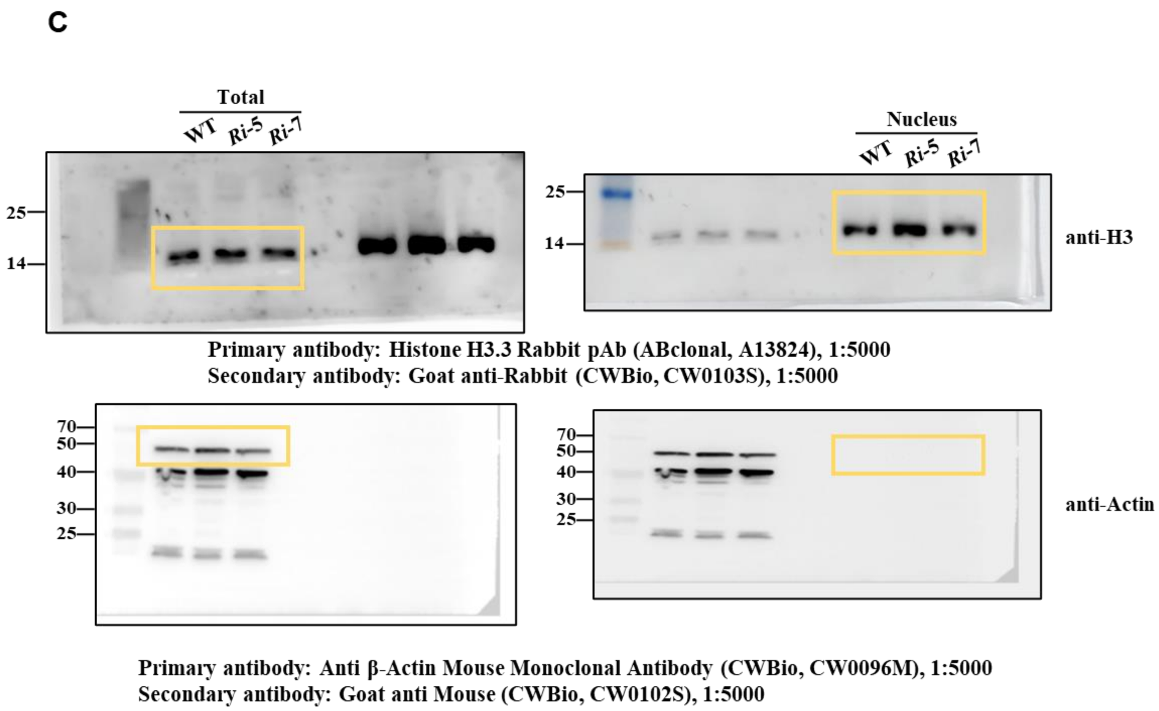
C
Total
WT
Ri-5
Ri-7
25
14
Nucleus
WT
Ri-5
Ri-7
25
14
anti-H3
Primary antibody: Histone H3.3 Rabbit pAb (ABclonal, A13824), 1:5000
Secondary antibody: Goat anti-Rabbit (CWBio, CW0103S), 1:5000
70
50
40
30
25
70
50
40
30
25
anti-Actin
Primary antibody: Anti β-Actin Mouse Monoclonal Antibody (CWBio, CW0096M), 1:5000
Secondary antibody: Goat anti Mouse (CWBio, CW0102S), 1:5000

Supplemental Figure S9

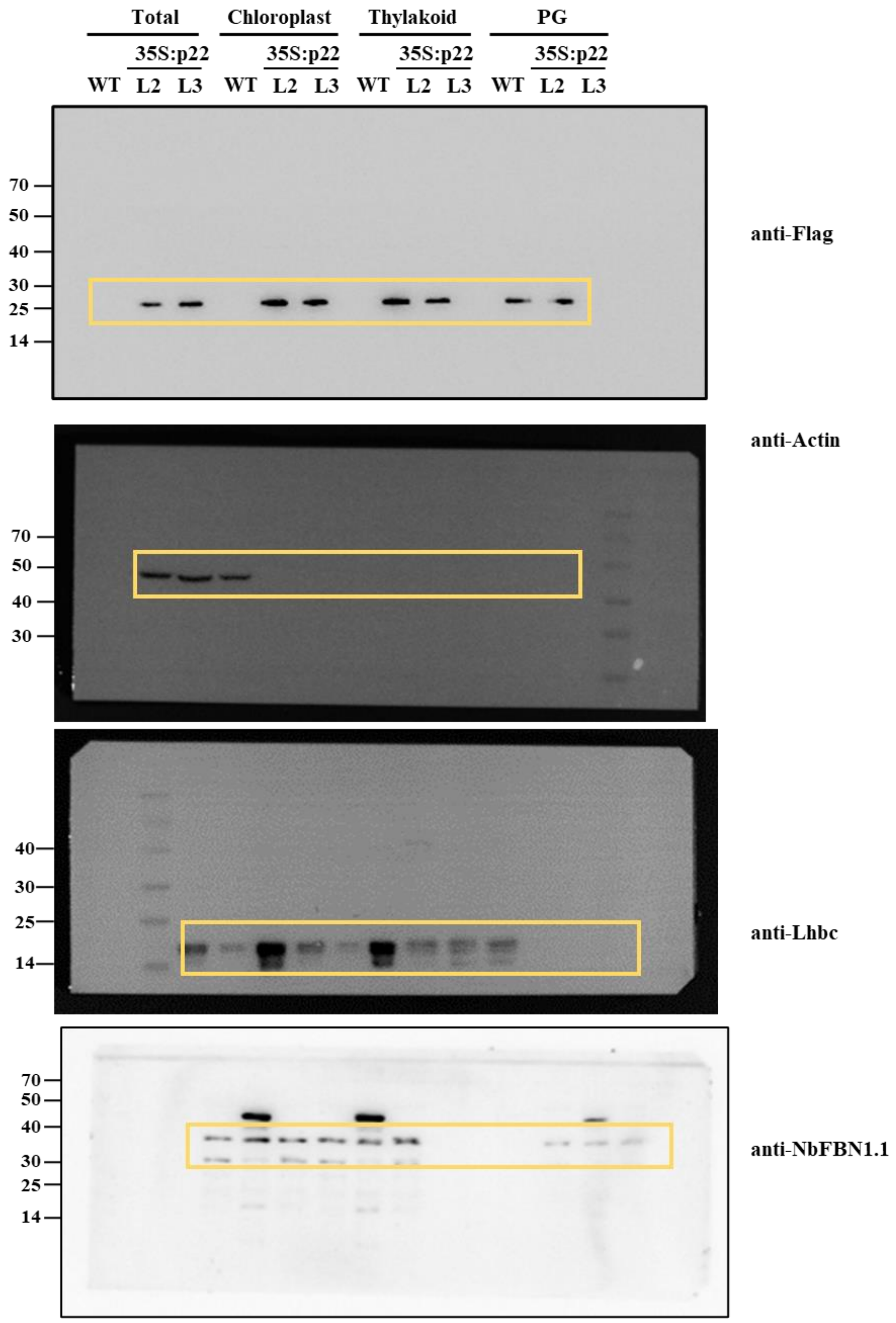

Primary antibody: Anti-Flag M2-Peroxidase (HRP) antibody (Sigma, A8592), 1:10000
Anti β-Actin Mouse Monoclonal Antibody (CWBio, CW0096M), 1:5000
Anti-Lhcb1 Rabbit Polyclonal Antibody (Agrisera, AS09-522), 1:5000
Anti-FBN1.1, Rabbit Polypeptides Epitope (LITKLESMNPTPAPC), 1:1000

Secondary antibody: Goat anti-Mouse (CWBio, CW0102S), 1:5000
Goat anti-Rabbit (CWBio, CW0103S), 1:5000

Supplemental Figure S10

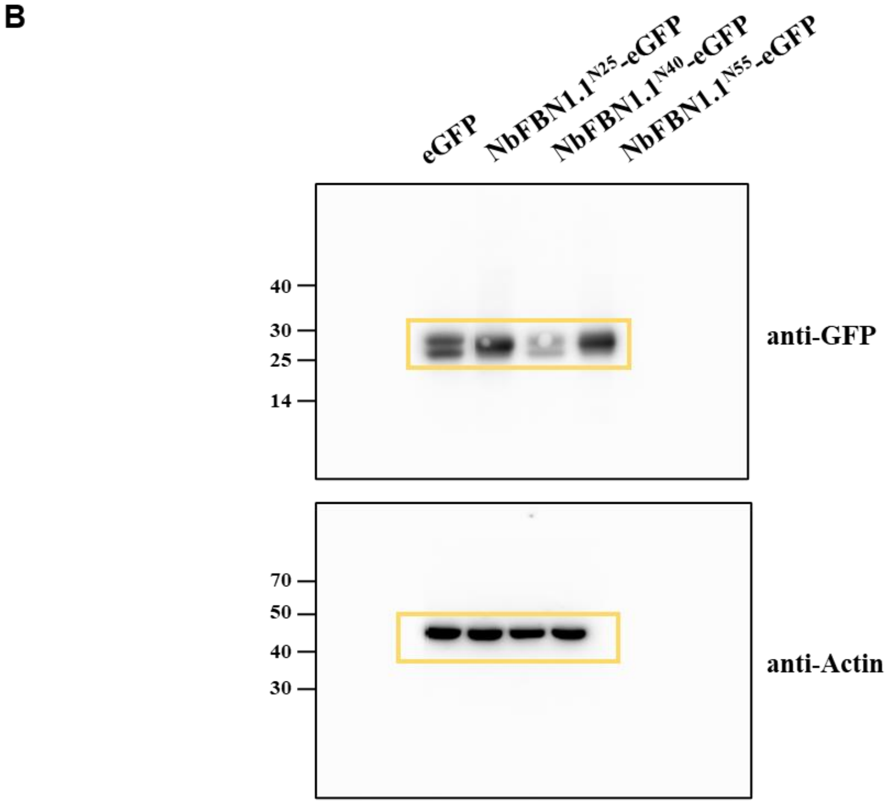

Primary antibody: Anti-GFP Mouse Monoclonal Antibody (TransGene Biotech, HT801-01), 1:10000
Anti β-Actin Mouse Monoclonal Antibody (CWBio, CW0096M), 1:5000
Secondary antibody: Goat anti-Mouse (CWBio, CW0102S), 1:5000

Supplemental Figure S11

C

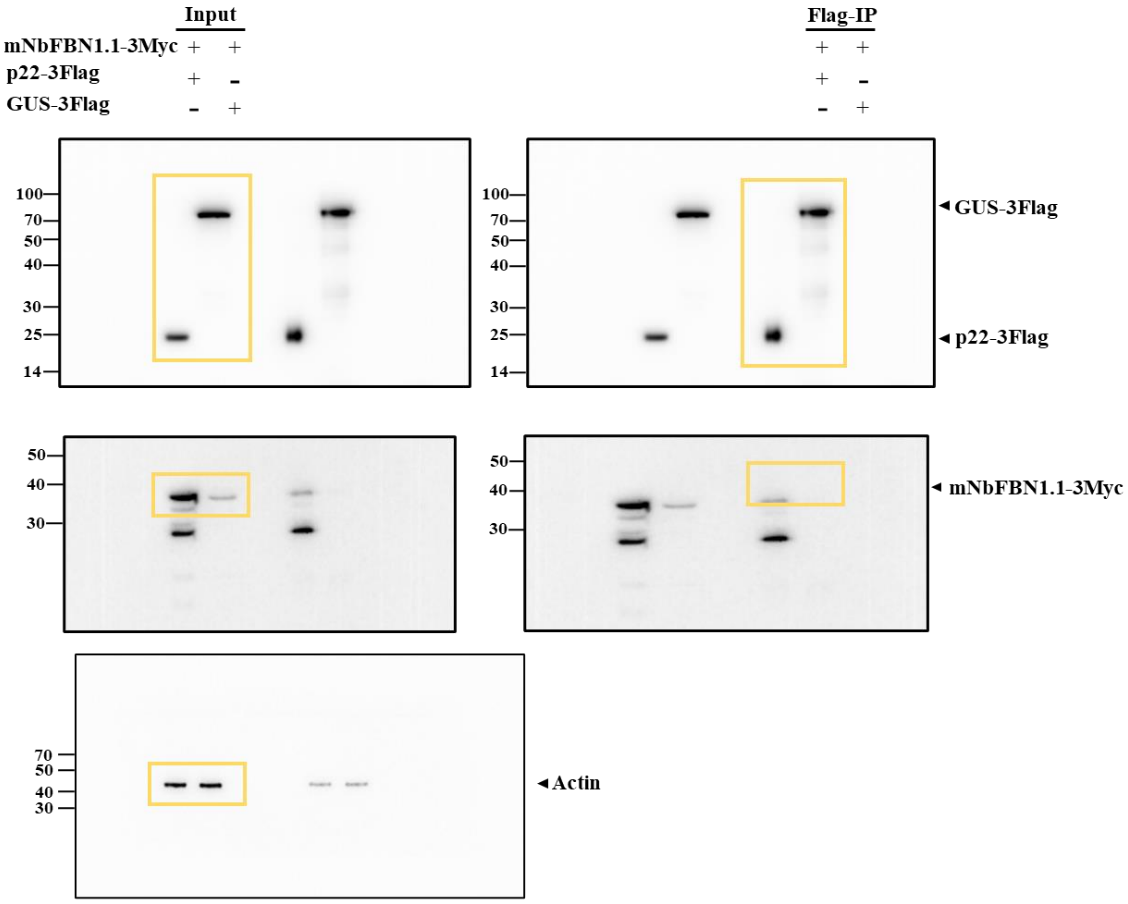

Primary antibody: Anti-Flag M2-Peroxidase (HRP) antibody (Sigma, A8592), 1:10000
Anti-c-Myc Peroxidase (HRP) antibody (Sigma, A5598), 1:5000
Anti β-Actin Mouse Monoclonal Antibody (CWBio, CW0096M), 1:5000
Secondary antibody: Goat anti-Mouse (CWBio, CW0102S), 1:5000

Supplemental Figure S18

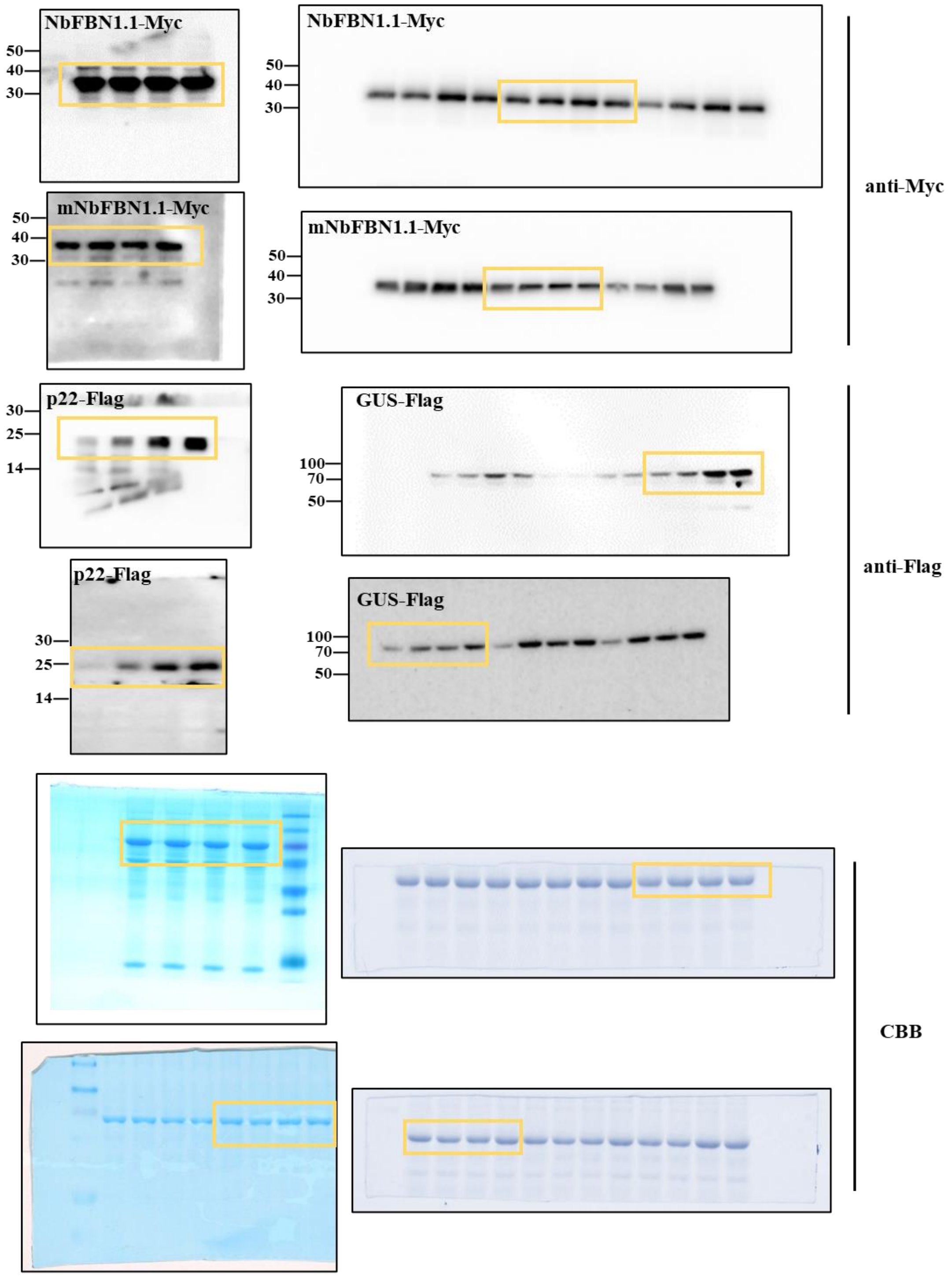

Primary antibody: **Anti-Flag M2-Peroxidase (HRP) antibody (Sigma, A8592), 1:10000**
Anti-c-Myc Peroxidase (HRP) antibody (Sigma, A5598), 1:5000

Supplemental Figure S19

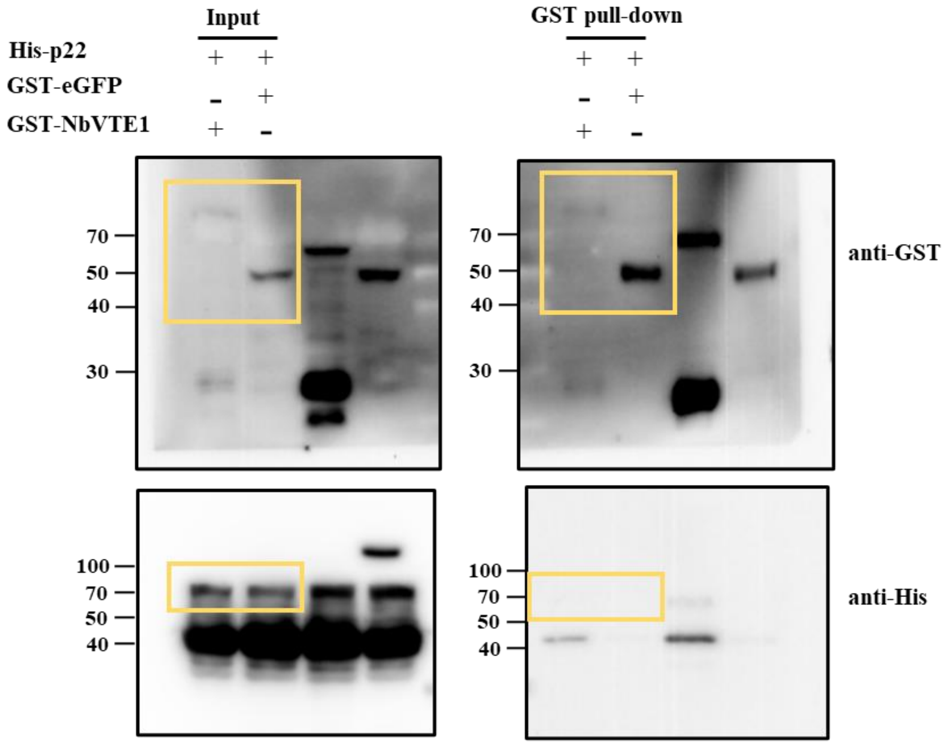

Primary antibody: Anti-GST-Tag Mouse Monoclonal Antibody (CWBio, CW0084M), 1:5000
Anti- His-Tag Mouse Monoclonal Antibody (CWBio, CW0286M), 1:5000

Secondary antibody: Goat anti-Mouse (CWBio, CW0102S), 1:5000

Supplemental Figure S26

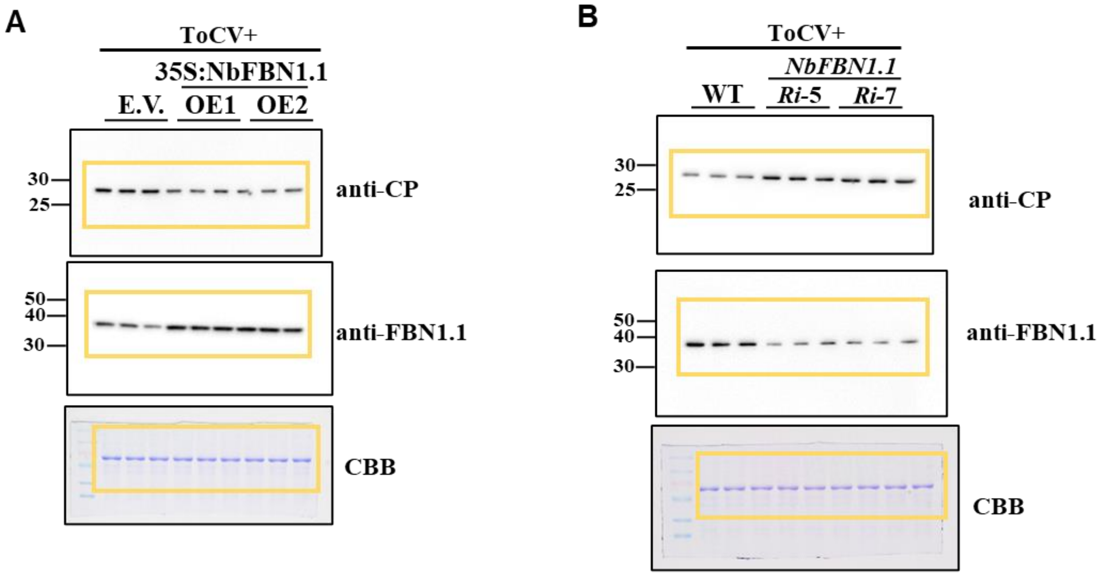

Primary antibody: Anti-CP, Rabbit Polypeptides Epitope (NGGDRNPLVRPLDDC), 1:1000
Anti-FBN1.1, Rabbit Polypeptides Epitope (LITKLESMNPTPAPC), 1:1000

Secondary antibody: Goat anti-Rabbit (CWBio, CW0103S), 1:5000

Supplemental Figure S27

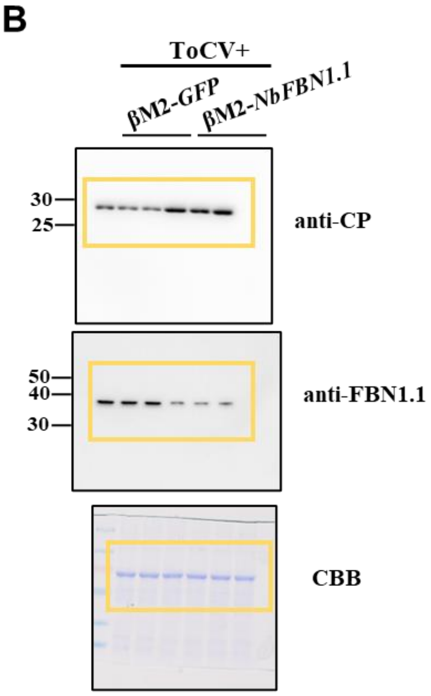

Primary antibody: Anti-CP, Rabbit Polypeptides Epitope (NGGDRNPLVRPLDDC), 1:1000
Anti-FBN1.1, Rabbit Polypeptides Epitope (LITKLESMNPTPAPC), 1:1000
Secondary antibody: Goat anti-Rabbit (CWBio, CW0103S), 1:5000
